# Evaluating genome-wide DNA methylation changes in mice by Methylation Specific Digital Karyotyping

**DOI:** 10.1186/1471-2164-9-598

**Published:** 2008-12-11

**Authors:** Kathy Boon, John K Tomfohr, Nathaniel W Bailey, Stavros Garantziotis, Zhuowei Li, David M Brass, Shuichiro Maruoka, John W Hollingsworth, David A Schwartz

**Affiliations:** 1 National Heart Lung and Blood Institute/National Institute of Environmental Health Sciences, Research Triangle Park, NC 27709, USA; 2 Duke University Medical Center, Durham, NC 27710, USA; 3 National Jewish Health, Denver, CO 80206, USA; 4 University of Colorado Health Sciences Center, Denver, CO 80206, USA

## Abstract

**Background:**

The study of genome-wide DNA methylation changes has become more accessible with the development of various array-based technologies though when studying species other than human the choice of applications are limited and not always within reach. In this study, we adapted and tested the applicability of Methylation Specific Digital Karyotyping (MSDK), a non-array based method, for the prospective analysis of epigenetic changes after perinatal nutritional modifications in a mouse model of allergic airway disease. MSDK is a sequenced based method that allows a comprehensive and unbiased methylation profiling. The method generates 21 base pairs long sequence tags derived from specific locations in the genome. The resulting tag frequencies determine in a quantitative manner the methylation level of the corresponding loci.

**Results:**

Genomic DNA from whole lung was isolated and subjected to MSDK analysis using the methylation-sensitive enzyme *Not *I as the mapping enzyme and *Nla *III as the fragmenting enzyme. In a pair wise comparison of the generated mouse MSDK libraries we identified 158 loci that are significantly differentially methylated (P-value = 0.05) after perinatal dietary changes in our mouse model. Quantitative methylation specific PCR and sequence analysis of bisulfate modified genomic DNA confirmed changes in methylation at specific loci. Differences in genomic MSDK tag counts for a selected set of genes, correlated well with changes in transcription levels as measured by real-time PCR. Furthermore serial analysis of gene expression profiling demonstrated a dramatic difference in expressed transcripts in mice exposed to perinatal nutritional changes.

**Conclusion:**

The genome-wide methylation survey applied in this study allowed for an unbiased methylation profiling revealing subtle changes in DNA methylation in mice maternally exposed to dietary changes in methyl-donor content. The MSDK method is applicable for mouse models of complex human diseases in a mixed cell population and might be a valuable technology to determine whether environmental exposures can lead to epigenetic changes.

## Background

In eukaryotes DNA methylation occurs on cytosine residues of CpG dinucleotides. CpG islands, short genomic regions with a high frequency of CpG dinucleotides, are typically common near transcription start sites, and may be associated with promoter regions. These regions are not generally methylated in contrast to highly repetitive elements in the genome. DNA methylation can directly affect transcription by inhibiting binding of specific transcription factors and/or enhancing necessary chromatin remodeling for gene silencing. This process is required for normal embryonic development, imprinting, and X chromosome inactivation, and plays an important role in the normal functioning of an organism. Increasing numbers of studies are emphasizing the role of DNA methylation in human diseases [1-3]. Only until recently it has become clear that nutritional components can also affect epigenetic mechanisms like DNA methylation and can lead to long term phenotypic changes [4,5].

The study of DNA Methylation has become more accessible by recent development of various technologies [6]. The choice of methodology is highly dependent of the goal of the study, genome of interest and available resources. Most commercially available micro-array platforms are developed for restriction enzyme and affinity based assays like the short oligonucleotide arrays (Affymetrix), tiling arrays (NimbleGen) and CpG Island/promoter arrays (Agilent) [6]. Like in other micro-array assays intensity changes are measured instead of actual levels of methylation and are subject for many sources of variation like array-lot variability and washing conditions. Whether the hybridization intensity will be proportional to the level of methylation is still uncertain and could be platform dependent. While these approaches are able to evaluate methylation changes throughout the entire genome, they remain expensive and are not generally accessible. Methylation Specific Digital Karyotyping (MSDK) is a non-micro array, genome-wide methylation analysis approach that relies on cleavage of genomic DNA with a methylation sensitive restriction enzyme. The concept of this approach is similar to serial analysis of gene expression (SAGE) and is based on the following principles: 21 bp sequence tags are derived from specific locations within the genome and can be directly matched to their corresponding loci, and concatenation of the sequence tags allows the serial analysis of multiple tags by high throughput sequencing [7,8]. The resulting genomic tag frequencies determine in a quantitative manner the methylation level of the corresponding loci. In this report, we present a modification and application of MSDK, for the study of methylated loci throughout the mouse genome prenatally exposed to nutritional variations.

## Results and discussion

### Genome-wide DNA methylation analysis

In an effort to uncover candidate genes directly affected by DNA methylation in a mouse model of perinatal nutritional modification and allergic airway disease, we applied MSDK, a comprehensive, quantitative and unbiased genome-wide method that offers accurate information on the genomic position of differentially methylated loci. The MSDK method has been previously developed for the analysis of the human genome using the restriction enzyme *Asc *I as the mapping enzyme. The *Asc *I recognition site is preferentially found in CpG islands associated with transcribed genes [7]. For our study of the mouse genome we considered the methylation sensitive enzymes *Asc *I, *Sac *II, *Not *I and *Hpa *II as the mapping enzyme in combination with *Nla *III as the fragmenting enzyme. There are over a million restriction sites for *Hpa *II in the mouse genome and 38,684 for *Sac *II. Although both enzymes are excellent cutters, we concluded that the sequencing costs would be too high. We found only 2,500 restriction sites for *Asc *I and 6,012 sites for *Not *I with a respective average fragment size of 280,000 bp and 130,000 bp. We selected *Not *I since the recognition site contains two CG dinucleotides and approximately 62% of these sites are associated with a CpG island in the mouse genome. Though not all the genes are associated with a CpG Island it has been estimated that at least 40% of the annotated genes have a CpG Island in their corresponding promoter region.

**Figure 1:**
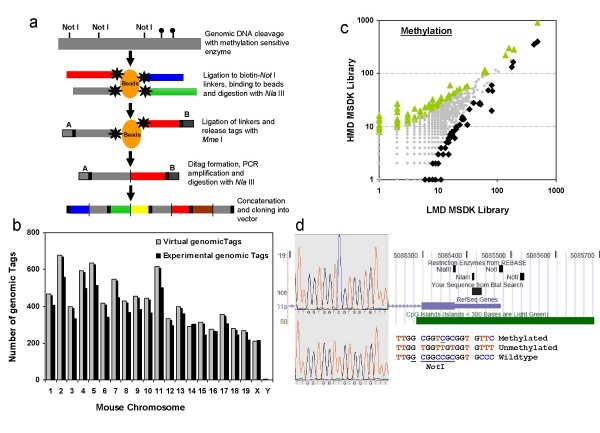
**a. Schematic view of the generation of a *Not *I MSDK library.** The black lollipops indicate methylated *Not *I sites that will not be digested by the enzyme. 1b. The number of virtual tags (lined bars) per chromosome compared to the number of experimental tags (black bars) shows an unbiased sampling of the mouse genome. On average > 80% of experimental tags could be matched to a virtual tag after removal of all singletons from the pool of *Not *I MSDK genomic tags. 1c. A log10 scale scatter plot comparison of genomic tag counts between the HMD (y-axis) and the LMD (x-axis) MSDK libraries. Significant differentially methylated genomic tags with a P-value = 0.05 with higher counts in HMD are indicated by green triangles and those with higher counts in LMD are indicated by black diamonds. Points colored in grey are considered non-significant (P-value > 0.05). 1d. Snapshot UCSC genome Browser displaying the location of the genomic tag found to be differentially methylated and associated with Tmem151a on chromosome 19 (Table 2). The *Not *I restriction sites are also indicated. MSP was performed and the PCR products were sub cloned into a TOPO TA sequencing vector. Up to 12 independent clones with insert were selected for sequencing. The Sequence analysis of bisulfate modified genomic DNA from 2 HMD lung tissue samples revealed a methylated *Not *I site in the proximity of the gene encoding hypothetical protein Tmem151a in 2 out of 12 sequenced clones.

For the generation of the mouse MSDK libraries we chose lung tissue from C57BL/6J adult mice that were prenatally exposed to diets with increased (high methylation diet; HMD) or decreased (low methylation diet; LMD) methyl-donor content and challenged with allergen in adulthood [9]. The mice gestated on both diets developed features characteristic of an allergic phenotype including airway hyper-reactivity, lung lavage eosinophilia, and increase serum IgE concentrations [9]. Genomic DNA was isolated from snap frozen lung tissue samples from two male mice for each group using a DNeasy kit (Qiagen, Valencia, CA) according to manufacturer instructions. Library construction was essentially performed as described for Digital Karyotyping with a few minor modifications [10]. The genomic DNA integrity was accessed by digestion with *Sac *I before use in library construction. For each library 2 μg of genomic DNA was digested with the mapping enzyme *Not *I, ligated to biotinylated *Not *I linkers (Figure 1a) and digested with the fragmenting enzyme *Nla *III. DNA fragments containing the biotinylated linkers were isolated using streptavidin-coated magnetic beads (Dynal Biotech, Brown Deer, WI) and ligated to linkers including recognition sites for *Mme *I. The 21 bp sequence tags were released by digestion with *Mme *I as has been described for Long SAGE [11]. The isolated tags are self-ligated, PCR amplified, concatenated, cloned in pZero (Invitrogen, Carlsbad, CA), and sequenced. DNA from plasmid inserts containing serial genomic tags were purified and sequenced at Agencourt Bioscience Corporation (Beverly, MA). The SAGE 2000 software package enables the extraction of the genomic tags from the sequence files. Tag sequences, tag counts and gene associations were stored in a Microsoft Access relational database for subsequent selection and matching to virtual tags.

We sequenced 66,758 and 69,498 tags for respectively the HMD and the LMD MSDK libraries. After removal of all singletons, we found 8,323 experimental genomic tags of which 6,310 were present in both libraries, 967 were specific for the HMD and 1046 for the LMD library. In order to match the experimental tags to their corresponding genomic loci, virtual genomic tags were extracted from the mouse genome sequence as obtained from Ensembl  using a python script . This identified a total of 10,577 virtual tags of which 8,414 are unique. Alignment of the experimental tags with the 10,577 virtual tags showed that 5,533 (67%) could be matched to a unique position within the genome. A remaining 1,614 (19%) matched to multiple regions in the genome and 1,176 (14%) were not found. It is expected that the percentage of unmatched tags will decrease in time with the new releases of updated public mouse genome sequences. Comparison of the experimental tags with the virtual genomic tags per chromosome revealed an unbiased distribution of the experimental tags over the mouse genome (Figure 1b).

We identify differentially methylated loci in the mouse genome between the two libraries by applying the following selection criteria: mapping to a unique position to the mouse genome, and a fold difference in tag counts ≥ 5. Furthermore, in order to find genomic tags with a significant difference in tag counts we used a z-score to quantify the strength of the observed difference in the proportions of individual tag sequences in the two libraries and generate a P-value. We considered a P-value ≤ 0.05 as significant. Similar results were obtained when analyzing the data according to the sequence odds ratio and significance test  or the significance test available as part of the DiscoverySpace application [12]. In this way, we identified 82 genomic tags with a higher count in the LMD library and 71 genomic tags with higher counts in the HMD [see Additional files 1 and 2] representing differentially methylated loci within the mouse genome (Figure 1c). The MSDK method screens for unmethylated restriction enzyme sites in the genome therefore higher genomic tag frequencies correspond with a decreased level of methylation. This implies that the 82 genomic tags with increased counts in the LMD library are likely to show an increased level of methylation in the HMD library. Despite the limited sequence depth (70,000 genomic tags per library) we were able to find novel differentially methylated loci in our mouse model. Since most of the *Not *I sites are correlated with a CpG Island in the genome it can be expected that the differences in tag counts as identified by MSDK correlate with changes in transcription levels, which ultimately can influence the phenotype seen in our mouse model.

**Figure 2:**
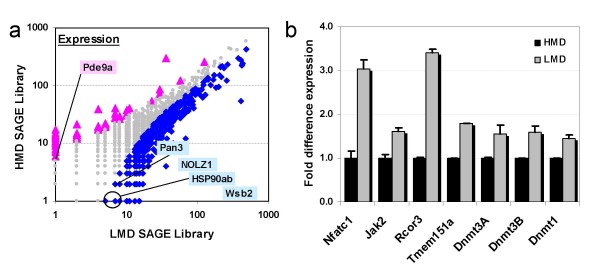
**a. A log10 scale scatter plot comparison of the transcript sequence tag counts as assessed by SAGE analysis between the HMD (y-axis) and the LMD (x-axis) libraries.** Significant differentially expressed transcripts with a P-value = 0.05 with higher expression in HMD are indicated by pink triangles and those with higher expression in LMD are indicated by blue triangles. Points colored in grey are considered non-significant (P-value > 0.05). A few representative genes are indicated by their corresponding gene symbol. Pde9a, phosphodiesterase 9A; Nolz1, zinc-finger protein Nolz1; Pan3, Pan3 polyA specific ribonuclease subunit homolog; HSP90ab, heat shock protein 90 kDa alpha, classB1; Wsb2, WD repeat and SOCS box-containing 2. 2b. Relative mRNA expression was measured by real-time PCR using SYBR-Green as described in Materials and Methods. Comparison between HMD/high responders (n = 6) and LMD/low responders (n = 6) in whole lung tissue obtained from challenged C57/BL6 mice. The values for the HMD samples were arbitrarily set to 1 in order to display the fold difference. P-values were calculated according to the Mann-Whitney U Test: Nfact1 = 0.004; Jak2 = 0.077; Rcor3 = 0.002; Tmem151a = 0.179; Dnmt3A = 0.051; Dnmt3B = 0.041; Dnmt1 = 0.002. All data are presented as the mean ± standard error of the mean.

To validate the MSDK results we isolated total RNA from the same samples used in the MSDK analysis and performed Serial Analysis of Gene Expression (SAGE) profiling of these samples. This generated 102,868 and 68,778 transcript tags for respectively the HMD and LMD SAGE library. After normalization to correct for the differences in total tag counts in each SAGE library, we selected for transcripts with a fold-difference ≥ 5 and a P-value ≤ 0.05 as previously described [13]. Surprisingly we found 139 transcripts to be over expressed in the HMD SAGE library and about 1254 in the LMD SAGE library [see Additional files 3 and 4]. This is an almost 9-fold increase of transcription in the LMD sample (Figure 2a). Most likely this is due to a combination of increased DNA methylation in the HMD samples and a reduced amount of methyl donors in the LMD resulting in hypomethylation and poorly regulated transcription. It is important to note that not all methylation changes will affect transcriptional levels. Still, we could identify genes predicted to be methylated by MSDK analysis and with increased mRNA levels in the LMD SAGE library (Table 1). We also selected four genes from the list of differentially methylated loci for which the *Not *I site was located within a CpG Island in close proximity of the predicted transcription start site (based on the February 2006 Mouse Genome Assembly, ), and which were not expressed in sufficiently high levels to be significantly differentially expressed in the SAGE analysis (Table 2). Real-time PCR demonstrated an increase of expression level for Nfatc1, Jak2, Rcor3, Tmem151a, and the methyl-tranferases Dnmt1, Dnmt3A and Dnmt3B, in lung tissue from an independent set of animals prenatally exposed to a diet low in methyldonor content (LMD, Figure 2b). Similar results were obtained in lymphocyte-mononuclear cells isolated from spleen of HMD and LMD animals [9]. An increased expression of the methyl-transferases, especially those involve in *de novo *methylation (Dnmt3A and 3B), might partially account for the unexpected methylated genomic loci in the LMD library when compared to the HMD library [see Additional file 2] although other mechanisms of transcription regulation can not be excluded. Taken together the SAGE profiling and the real-time PCR confirmed the predicted changes in expression level by MSDK analysis.

**Table 1:** Comparison between MSDK and SAGE

**Methylation Specific Digital Karyotyping**			**Gene Information**		**Serial Analysis of Gene Expression**		
MSDK Genomic Tag Sequence	MSDK LMD	MSDKHMD	Gene Symbol	Gene Description	SAGE Tag Sequence	SAGE LMD	SAGE HMD

ACCTACCCAGGCAGCCT	8	0	Zfp503	zinc-finger protein NOLZ1	TTTGTTACAA	5	0
GCAGCGTCCCGGGTCGG	6	0	Hsp90ab1	heat shock protein 90 kDa alpha, B1	GTGAGCCCAT	4	0
AACAGTGGCGGCGGCGG	5	0	Pan3	PAN3 polyA specific ribonuclease subunit	TCGCGTCGCT	7	2
GCGCAGGCGACCCGGGG	6	1	Wsb2	WD repeat and SOCS box-containing 2	GGATGTACCC	5	1
AACAAAAAGGGTCTGTG	2	10	Pde9a	phosphodiesterase 9A	TAAATTCCAC	0	6

Genomic tags are indicated as 17 bp sequences with the corresponding counts as found in each MSDK library. Transcript tags are indicated as 10 bp sequences with the corresponding expression level as found in each SAGE library. Genomic DNA and RNA for MSDK and SAGE analysis was isolated from lung tissue from C57BL/6J adult mice that were prenatally exposed to nutritional changes as described in [9].

**Table 2:** Selected candidate genes for real-time PCR analysis

***Genomic Tag***	***LMD***	***HMD***	***Chr***	***Position***	***Strand***	***Gene***
CGACAGAGGGCCGGGGG	6	0	18	80870439	-	Nfatc1 (nuclear factor of activated T-cells)
CAGAATGGGTGCTGCCT	6	0	19	29317014	+	Jak2 (Janus kinase 2)
GTAGAGGAGGGGGAGAG	8	0	1	193838624	+	Rcor3 (REST corepressor 3)
ATAAGCAGGGGTGCGGG	8	0	19	5085417	+	Tmem151a (Hypothetical protein LOC381199)

Genomic tags are indicated as 17 bp sequences with the corresponding counts as found in each MSDK library, location within the genome, strand orientation and associated gene. A complete list can be found in Additional file 1 and [9].

A quantitative MSP assay was developed for parts of a CpG Island in close proximity of the genomic tags matched to Runx3 and Nfatc1. Although this assay demonstrated an increased methylation level (10 to 50% ± 20%) in the genomic region analyzed in lung tissue and spleen, the results could be masked due to the use of a mixed cell population. Also the length of the CpG Island (over 1000 bp for Runx3) makes it difficult to design the assays. An extensive *in vitro *promoter analysis might be necessary to clearly identify the specific regulatory elements susceptible for methylation. Interestingly, the transcription factor Runx3 has been shown to be regulated by promoter methylation in human cancers [14,15] and it was also shown that Runx3 can be down regulated by an increase of Histone H3 methylation in human cancer cell lines [16]. More recently we have shown [9], that a demethylating agent can restore the transcription levels of Runx3 in spleen cells from HMD animals, implying that the transcriptional changes are caused by methylation changes. The expression of nuclear factor of activated T cell 1 (Nfatc1) has also been described to be regulated by hypermethylation in Hodgkin's lymphoma as well as by histone H3 acetylation and H3-K4 trimethylation [17]. It is not known whether Rcor3 (Rest corepressor 3) it self is regulated by methylation though depletion of Corest can result in derepression of REST responsive gene expression and increased methylation of Histone H3-K4 [18,19]. Other examples of genes regulated by methylation that have been identified in our study as differentially methylated candidate genes [see Additional files 1 and 2] can be found in the literature like Cited4, which is epigenetically silenced in the vast majority of human oligodendroglial tumors [20]. Taken together these findings indicate that it is very likely that our list of differentially methylated loci is actually regulated by DNA methylation but it also indicates that the contribution of other epigenetic mechanisms like histone modifications affecting the accessibility of chromatin can not be excluded.

### Differential gene expression and functional analysis

**Figure 3:**
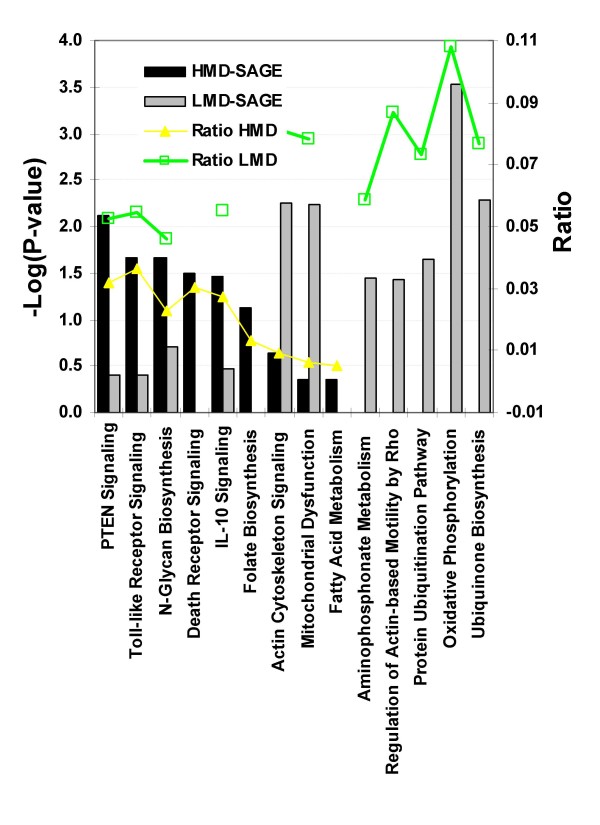
**Pathway analysis.** Most significant canonical pathways associated with the HMD and LMD SAGE expression profiles according to the IPA pathway analysis tool. The significance of the association between the dataset and the canonical pathway was measured as a ratio (number of genes from the dataset that map to the pathway divided by the total number of molecules that exist in the canonical pathway). A Fischer's Exact Test was used to calculate a P-value.

We applied the Ingenuity Pathway Analysis program to explore the list of differentially expressed genes. The top canonical pathways that are significantly associated with the differentially expressed genes in the lungs of HMD or LMD animals are represented in Figure 3. The significance is determined by a high ratio (or percentage of genes in pathway found in the gene list) and by a high negative logarithm of the P-value; indicating that the pathway is significantly associated with the data and that a large portion of the corresponding canonical pathway may be affected. Interestingly, we see an association of the folate biosynthesis pathway in the HMD animals. This pathway plays a role in the synthesis of S-adenosyl methione, the main methyl donor group in the process of DNA methylation [21] implying an association between the maternal nutritional intake and increase of DNA methylation in this group. Dietary changes can clearly have an effect on the transcriptional profiles thereby adding another variably that can affect the phenotypic responses to exposure.

## Conclusion

Perinatal dietary changes in methyl donor content can lead to alterations in expression profiles as well as subtle changes in DNA methylation. Using MSDK, a method that allows a comprehensive and unbiased genome-wide methylation profiling, we uncovered differentially methylated loci in the progeny of mice maternally subjected to diets high and low in methyl donors. The depth and coverage of the approach is dependent on the restriction enzyme used and can be adjusted as needed. The MSDK technology is sequence based, quantitative and unbiased without the requirement of special equipment other than a DNA sequencer. An increase in throughput and large reduction of the sequencing costs is expected with the recent development of multiplex paired-end ditag sequencing technologies [22,23]. Most importantly; the data is digitally archived, relative easy to analyze and independent of an arbitrarily chosen reference. MSDK is applicable for mouse models in a mixed cell population, and may be a useful approach to determine whether environmental exposures can lead to epigenetic changes in complex heritable human diseases.

## Methods

### Primers and linkers

**Not-MSDK linkers: **5'-/5Phos/GGCCGCACCCAACTCGATTACGAACCTCTGC-3' and 5'-/5Bio/TTTGCAGAGGTTCGTAATCGAGTTGGGTGC-3'. These linkers were obtained PAGE purified and modified from Integrated DNA Technologies, Coralville, IA.

**RT-PCR primers: **Jak2 Forward 5'-TCTGTGGGAGATCTGCAGTG-3', Jak2 Reverse 5'-CACGGATGACAGCTCTGAAA-3'; Nfatc1 Forward 5'-TCATCCTGTCCAACACCAAA-3', Nfatc1 Reverse 5'-TCACCCTGGTGTTCTTCCTC-3'; Rcor3 Forward 5'-CATGGATGGAAACGACAGTG-3', Rcor3 Reverse 5'-AGTTGCCTCAGGATGGTGTT-3'; B2M Forward 5'-ATTCACCCCCACTGAGACTG-3', B2M Reverse 5'-GCTATTTCTTTCTGCGTGCAT-3'; PGK1 Forward 5'-CAAGGCTTTGGAGAGTCCAG-3', PGK1 Reverse 5'-TGTGCCAATCTCCATGTTGT-3'; Actb Forward 5'-TCCGTAAAGACCTCTATGCC-3'; Actb Reverse 5'-TACTCCTGCTTGCTGATCC-3'

Tmem151a Forward 5'-AGGGCGAAGGTGGAGACT-3', Tmem151 Reverse 5'-GGCATGGATGAGCAGTGTAA-3'; mDnmt3a Forward 5'-TGACGCCAAAGAAGTGTCTG-3, mDnmt3a Reverse 5'-TTCAGTGCACCACAGGATGT-3'; mDnmt3b Forward 5'-ACTTGGTGATTGGTGGAAGC-3', mDnmt3b Reverse 5'-CCAGAAGAATGGACGGTTGT-3'; mDnmt1 Forward 5'-TCCTCAGGGACCATATCTGC-3', mDnmt1 Reverse 5'-CTGCACAGGAACAGACTCCA-3'. **MSP primers**: Runx3 methylated forward 5'-AGAAAATCGTTTTGGGTGTTATC-3', reverse 5'-AATTTCCAACCTCCTAACTACGAC-3'; Nfatc1 methylated forward 5'-GTAGTTTAGTTAGGGAGGAGGATTC-3', reverse 5'-TACGAAAACGAAAAAACTTTACGAC-3'; Actb methylated forward 5'-TGTGATTGATAGTAGGAAGGTGTGA-3'; reverse 5'-ACCCAAATCCAAAAATCACG-3'.

### SAGE library construction and statistical analysis of tag counts differences

SAGE libraries from the same samples used for MSDK analysis were constructed using *Nla *III as the anchoring enzyme and *BsmF *I as the tagging enzyme according to a micro-SAGE protocol [13]. The SAGE library clones were arrayed and inserts were purified and sequenced at Agencourt Bioscience Corporation. The SAGE 2000 software version 4.12 (available at ) was used to extract SAGE tags from the original sequence files, remove duplicate ditags, remove linker sequences, remove one base pair variations of linker sequences and tabulate the occurrence of each tag. Tag sequences, tag counts and gene associations were stored in a Microsoft Access relational database for subsequent analysis. P-values for differentially expressed transcripts were calculated according to the sequence odds ratio and significant test ). Similar results were obtained when using the SAGE software Monte Carlo approach or the significance test available as part of the DiscoverySpace application [12]. The generated mouse SAGE libraries have been deposit at the Geo Website (GSE11676).

### Gene Ontology and Canonical Pathway analysis

Ingenuity Pathway Analysis (IPA, Ingenuity Systems^®^, ) is a web-based application that enables the visualization, discovery and analysis of molecular interaction networks within gene expression profiles. The generated SAGE datasets and the corresponding expression levels, represented as the log_2 _ratios, were uploaded within the IPA database for further analysis. Both gene symbols and gene bank accession numbers were used with no apparent differences in results. These genes, called focus genes, were overlaid onto a global molecular network developed from information contained in the Ingenuity knowledge base. The IPA knowledge base represents a proprietary ontology of over 600,000 classes of biologic objects spanning genes, proteins, cells and cell components, anatomy, molecular and cellular processes, and small molecules. **Networks **of the focus genes were then algorithmically generated based on their connectivity. The **Functional Analysis **of a network identified the biological functions and/or diseases that were most significant to the genes in the network. The network genes associated with biological functions and/or diseases in the Ingenuity knowledge base were considered for the analysis. Fischer's exact test was used to calculate a P-value determining the probability that each biological function and/or disease assigned to that network is due to chance alone. **Canonical Pathways **Analysis identified the pathways from the Ingenuity Pathways Analysis library of canonical pathways that were most significant to the dataset. The significance of the association between the dataset and the canonical pathway was measured in 2 ways: 1) a ratio of the number of genes from the dataset that map to the pathway divided by the total number of molecules that exist in the canonical pathway is displayed. 2) Fischer's Exact Test was used to calculate a P-value.

### RNA isolation and real-time PCR

Total RNA was extracted from frozen lung tissue using the RNAgents total RNA isolation system (Promega, Madison, WI, USA). Equal amounts of total RNA (5 μg), as determined by the Ribo-Green RNA Quantification kit (Molecular Probes, Eugene, OR, USA), were used in a 20 μl cDNA synthesis reaction primed with oligo-dT (Superscript II; Invitrogen, Carlsbad, CA, USA). Control reactions were prepared in parallel without reverse transcriptase. Prior to cDNA synthesis, residual genomic DNA was removed from total RNA with a DNase I treatment (DNA-free; Ambion, Austin, TX, USA). Quantitative PCR was performed with a 7900TH Fast Real-Time PCR system (Applied Biosystems, Foster City, CA, USA) using SYBR-Green. PCR reactions were performed in triplicate, and the threshold cycle numbers were averaged. Gene expression levels were normalized to three genes: ACTB (actin, beta), B2M (beta-2-microglobulin and PGK1 (phosphoglycerate kinase 1). The relative expression levels were calculated in comparison to the levels in total RNA from naïve mouse brain (Ambion, Austin, TX) according to the Comparative C_t _method in which the relative expression equals 2^-ΔΔCt^. PCR primers were designed using the Primer 3 interface . Data is presented as the mean ± standard error of the mean. Significant differences between groups were identified by analysis of variance. Individual comparisons between groups were confirmed by the two-tailed Student's t test or the Mann-Whitney U Test. A P-value ≤ to 0.05 was considered significant.

### Methylation Specific PCR

Sodium bisulfite modification of 500 ng genomic mouse lung DNA was performed using the EZ DNA Methylation-Gold Kit (Zymo Research, Orange, CA) according to manufacture's instructions. Modified DNA was eluted from the column with 20 ul elution buffer, aliquoted and stored at -20°C. PCR reactions were performed essentially as described using 2 ul modified genomic DNA [24]. CpG islands at the 5' region and up to the first exon of each gene were identified using online tools . The corresponding sequences were downloaded for primer design using MethPrimer [25] and Primer3 . Primers were designed to contain at least one CpG dinucleotide. Amplified products were analyzed by gel electrophoresis. Data was represented as mean ± standard error of the mean. Significant differences between groups were identified by analysis of variance. Individual comparisons between groups were confirmed by the two-tailed Student's t test or the Mann-Whitney U test. A P-value = to 0.05 was considered significant.

### TopoA cloning and sequencing

Amplified MSP products were analyzed by gel electrophoresis prior to further processing and purified by phenol extraction and ethanol precipitation. Approximately 70 ng of PCR product was used in a ligation reaction according to manufactures instructions using a TOPO TA cloning kit for Sequencing (Invitrogen, Carlsbad, CA). Transformants were analyzed by colony PCR with universal M13 primers. These PCR products were subsequently purified using a QIAquick PCR Purification Kit (Qiagen USA) and eluded from the columns in a 30 ul volume. For the sequencing reaction 4 ul purified PCR product with 5 pmole T7 universal primer was used in combination with a Dynamic ET Terminator Cycle Sequencing Kit (Amersham Biosciences, Piscataway, NJ). Post-sequencing cleanup was performed according to manufactures instructions.

## Authors' contributions

KB designed, performed computational analyses and generated most of experimental data. NWB generated experimental data. JKT provided bioinformatics and statistical support. SM provided samples. SM, JHW, SG, DMB and ZL all contributed in the collection of mouse tissue samples. KB, JWH and DAS contributed to the overall design of this project, provided critique of the results, and participated in manuscript conceptual development and editing. All authors read and approved the final manuscript.

## Supplementary Material

Additional file 1 **Differentially methylated genomic tags and nearest genes.** Files 1 and 2 catalogs respectively the 82 genomic sequence tags with higher counts in the LMD library. Also indicated are the nearest genes. Click here for file

Additional file 2 **Differentially methylated genomic tags and nearest genes.** Catalogs the 71 genomic sequence tags with higher counts in the HMD MSDK library representing differentially methylated loci within the mouse genome. Also indicated are the nearest genes. Click here for file

Additional file 3 **Differentially expressed SAGE transcripts.** Represents the transcript tags significantly over expressed in the HMD SAGE library. Click here for file

Additional file 4 **Differentially expressed SAGE transcripts.** Represents the transcript tags significantly over expressed in the LMD SAGE library. Click here for file
