# Supplementary material for: Evaluating genome-wide DNA methylation changes in mice by Methylation Specific Digital Karyotyping
Source: BMC Genomics. 2008 Dec 11;9:598. doi: 10.1186/1471-2164-9-598 (PMC2621211; doi:10.1186/1471-2164-9-598)
Supplement: Additional file 1 — Differentially methylated genomic tags and nearest genes. Files 1 and 2 catalogs respectively the 82 genomic sequence tags with higher counts in the LMD library. Also indicated are the nearest genes. [file 1471-2164-9-598-S1.doc]

| Genomic Tag Sequence | LMD | HMD | Ratio | Chr | Position | Orientation | p value | location of tag relative to nearest gene | Symbol | Description |
| --- | --- | --- | --- | --- | --- | --- | --- | --- | --- | --- |
| GAGCACCTGGCTGGTGG | 15 | 3 | 5 | 2 | 84515311 | + | 0.001603 | 8k bp downstream of stop | Zdhhc5 | Zinc finger DHHC domain containing 5 |
| GAATCTGAAATCTGTCA | 11 | 1 | 11 | 19 | 27283105 | + | 0.001603 | 1k bp upstream of start | Vldlr | very low density lipoprotein receptor |
| AATCATTGTTTACCGGA | 8 | 0 | 8 | 4 | 128308015 | + | 0.002296 | 32k bp upstream of start | A3galt2 | alpha 1 3-galactosyltransferase 2 |
| ACCTACCCAGGCAGCCT | 8 | 0 | 8 | 14 | 20774293 | - | 0.002296 | 853 bp upstream of stop | Zfp503 | zinc-finger protein NOLZ1 |
| ATAAGCAGGGGTGCGGG | 8 | 0 | 8 | 19 | 5085417 | + | 0.002296 | 3k bp upstream of start | Tmem151 | Hypothetical protein LOC381199 |
| GTAGAGGAGGGGGAGAG | 8 | 0 | 8 | 1 | 193838624 | + | 0.002296 | 927 bp downstream of start | Rcor3 | REST corepressor 3 |
| CCAAGCAGGATCCCTCT | 7 | 0 | 7 | 3 | 88110325 | - | 0.004344 | 2k bp upstream of stop | Hapln2 | hyaluronan and proteoglycan link protein 2 |
| CTCGCCCTGCAACCCGG | 9 | 1 | 9 | 2 | 68271869 | - | 0.005526 | 83k bp upstream of start | Stk39 | Serine/threonine kinase 39 STE20/SPS1 homolog |
| CTGTCCCTGCCCATCTC | 9 | 1 | 9 | 4 | 120147333 | + | 0.005526 | 16k bp upstream of start | Cited4 | "Cbp/p300-interacting transactivator, with Glu/Asp-rich carboxy-terminal domain, 4 |
| TGCTCTGCCTTCCCCGT | 9 | 1 | 9 | X | 17133328 | + | 0.005526 | 63k bp upstream of start |  | Tag sequence surrounded by repeat elements |
| CAGAATGGGTGCTGCCT | 6 | 0 | 6 | 19 | 29317014 | + | 0.008280 | 1k bp upstream of start | Jak2 | Janus kinase 2 |
| AGCGCGTCGCCTTCGGG | 6 | 0 | 6 | 8 | 12397131 | + | 0.008280 |  | Sox1 | SRY (sex determining region Y)-box 1 |
| GCACTGCCCCCGCTATT | 6 | 0 | 6 | 14 | 30324020 | + | 0.008280 | 132 bp upstream of start | Eaf1 | ELL associated factor 1 |
| GCACTGCCCCCGCTATT | 6 | 0 | 6 | 14 | 30324020 | + | 0.008280 |  | Mett 16 | methyltransferase like 6 |
| TGTTCTGTCTTGCCTTC | 6 | 0 | 6 | 7 | 142077009 | + | 0.008280 | 12k bp downstream of stop | Krtap5-1 | keratin associated protein 5-1 |
| GCAGCGTCCCGGGTCGG | 6 | 0 | 6 | 17 | 45036480 | + | 0.008280 | 4k bp upstream of start | Hsp90ab1 | "heat shock protein 90kDa alpha (cytosolic), class B member 1 |
| CATTTCGGAGTGGCGTC | 6 | 0 | 6 | 13 | 31818630 | + | 0.008280 | 4k bp downstream of stop | Foxc1 | forkhead box C1 |
| GAGTTGGCACAAGTAGG | 6 | 0 | 6 | 5 | 143783929 | - | 0.008280 |  | Rac1 | RAS-re;lated C3 botulinum substrate 1 |
| CTTAGGTTTGGGTCCTG | 6 | 0 | 6 | 11 | 11737408 | - | 0.008280 | 23k bp upstream of stop | Ddc | dopa decarboxylase (CpG island in middle of gene) |
| AAAAATCTAAGTTCAGA | 6 | 0 | 6 | 1 | 121663976 | + | 0.008280 |  | Ptpn4 | RIKEN full-length enriched library, clone:B430311G24 |
| CTGGCGGCGACCTGCAA | 6 | 0 | 6 | 2 | 73074000 | + | 0.008280 | 1k bp downstream of start | Sp9 | trans-acting transcription factor 9 |
| CGACAGAGGGCCGGGGG | 6 | 0 | 6 | 18 | 80870439 | - | 0.008280 |  | Nfatc1 | nuclear factor of activated T-cells |
| CCGACGTCATCCTCCAG | 6 | 0 | 6 | 8 | 73014094 | - | 0.008280 | 685 bp downstream of start | Hapln4 | hyaluronan and proteoglycan link protein 4 (brain link protein 2) |
| CTCTAGGGGCGCACGCT | 10 | 2 | 5 | 10 | 59502264 | + | 0.009987 | 439 bp downstream of start | Spock2 | "sparc/osteonectin, cwcv and kazal-like domains proteoglycan 2 |
| CTCCAAACCCAGCCTTC | 8 | 1 | 8 | 19 | 43493500 | + | 0.010307 | 1k bp upstream of start | Cnnm1 | cyclin M1 |
| GAAAACTTTTCGGCCGA | 8 | 1 | 8 | 12 | 79668652 | - | 0.010307 | 888 bp downstream of start | Mpp5 | "membrane protein, palmitoylated 5 (MAGUK p55 subfamily member 5) |
| GCCGTGCCACGGTCCCC | 8 | 1 | 8 | 6 | 83421861 | + | 0.010307 | 14k bp downstream of stop | AK218083 | Mus musculus cDNA, clone:Y2G0143P06. Alternative Splicing Library L13 |
| GCCGTGCCACGGTCCCC | 8 | 1 | 8 | 6 | 83421861 | + | 0.010307 |  | Dguok | Deoxyguanosine kinase |
| AACAGTGGCGGCGGCGG | 5 | 0 | 5 | 5 | 147740932 | + | 0.015936 | 306 bp upstream of start | Pan3 | PAN3 polyA specific ribonuclease subunit homolog (S. cerevisiae) |
| AGCGCAGCCGACTGCAC | 5 | 0 | 5 | 4 | 99149882 | - | 0.015936 | 1k bp upstream of stop | Foxd3 | forkhead box D3 |
| ATCCGCGGTGGGGGCCG | 5 | 0 | 5 | 5 | 32413267 | - | 0.015936 | 152 bp downstream of start | Fosl2 | fos-like antigen 2 |
| TTTATATTTTGGGAAGA | 5 | 0 | 5 | 17 | 26572054 | + | 0.015936 | 5k bp upstream of start | Nkx2-5 | "NK2 transcription factor related, locus 5 (Drosophila) |
| TTGGTCTCATTGTGCCT | 5 | 0 | 5 | 9 | 16253054 | - | 0.015936 | 127k bp upstream of start | XP_979253.1 | FAT tumor suppressor homolog 3 isoform 2 |
| TTCGCCACTTGGGCCCC | 5 | 0 | 5 | 10 | 79519355 | - | 0.015936 | 24 bp downstream of start | Stk11 | serine/threonine kinase 11 |
| TTCAGGCTGAGGCTTAA | 5 | 0 | 5 | 15 | 79632365 | - | 0.015936 | 18k bp upstream of stop | Cbx6 | neuronal pentraxin receptor |
| TCAGCCGCTCACCCAGC | 5 | 0 | 5 | 12 | 71371756 | - | 0.015936 | 15 bp downstream of start | Txndc1 | thioredoxin domain containing 1 |
| GCTCGGCGCTCAGCCCG | 5 | 0 | 5 | 17 | 25841032 | - | 0.015936 | 115 bp downstream of start | Tmem8 | transmembrane protein 8 (five membrane-spanning |
| GCGGCGGCGGCGGGGCG | 5 | 0 | 5 | 13 | 48979767 | + | 0.015936 | 68k bp downstream of stop | Wnk2 | WNK lysine deficient protein kinase 2 |
| GCGGCGGCGGCGACGAC | 5 | 0 | 5 | 11 | 87864016 | - | 0.015936 | 136 bp downstream of start | Sfrs1 | "splicing factor, arginine/serine-rich 1 (ASF/SF2) |
| GCCGCTCGGCCTCCCTC | 5 | 0 | 5 | 9 | 8134757 | + | 0.015936 | 8k bp upstream of start | AK129341 | Hypothetical protein LOC234915 |
| CTGTTTGCAGAGCCTGA | 5 | 0 | 5 | 1 | 193891215 | + | 0.015936 | 381 bp upstream of start | Kcnh1 | "potassium voltage-gated channel, subfamily H (eag-related), member 1 |
| GAGGCGCCAAGGACAGT | 5 | 0 | 5 | 17 | 4951893 | + | 0.015936 | 53k bp downstream of stop | Q3U196_MOUSE | "Activated spleen cDNA, RIKEN full-length enriched library, clone:F830009H16 product:hypothetical protein, full insert sequence. |
| CGGGCGCACGGGTAGTA | 5 | 0 | 5 | 6 | 52189815 | + | 0.015936 | 1k bp upstream of start | Hoxa13 | homeo box A13 |
| CGGCAGCTGTAGTAACC | 5 | 0 | 5 | 2 | 74469536 | - | 0.015936 | 387 bp downstream of start | Hoxd13 | homeo box D13 |
| CGCCAGGCTGTAGTCGC | 5 | 0 | 5 | 12 | 51570762 | + | 0.015936 | 22k bp downstream of stop | C130009A20Rik | RIKEN cDNA C130009A20 gene |
| CCGCGTCTCTGCTGCCC | 5 | 0 | 5 | 8 | 9771455 | + | 0.015936 | 92k bp upstream of start | Tmem28 | transmembrane protein 28 |
| GCAGGGACTCAAGGGGG | 5 | 0 | 5 | 12 | 77741159 | + | 0.015936 | 15k bp upstream of start | Fntb | "farnesyltransferase, CAAX box, beta |
| CTGCCGCTGTAGGAGGA | 7 | 1 | 7 | 2 | 167922644 | - | 0.019292 | 798 bp upstream of stop | Dpm1 | Dolichol-phosphate(beta-D)mannosyltransferase |
| TTTTGACCTTTTCAGTC | 7 | 1 | 7 | 4 | 140819726 | - | 0.019292 | 1k bp upstream of stop | B30016D10Rik | hypothetical protein LOC320456 |
| CAATCTTGAGCTGGGAA | 7 | 1 | 7 | 14 | 54047354 | - | 0.019292 | 1k bp upstream of start | Zfhx2 | zinc finger homeobox 2 |
| CCTAGCCACACTTCCCT | 7 | 1 | 7 | 2 | 167970651 | + | 0.019292 | 17k bp upstream of start | Dpm1 | dolichol-phosphate (beta-D) mannosyltransferase |
| CTCAGACCGCGCAGGTC | 7 | 1 | 7 | 1 | 84163695 | - | 0.019292 | 85k bp downstream of stop | Dner | delta/notch-like EGF-related receptor |
| CTCAGACCGCGCAGGTC | 7 | 1 | 7 | 1 | 84163695 | - | 0.019292 |  | 5033414K04Rik | hypothetical protein LOC98496 |
| CGTGCCGCGGGGGATGC | 7 | 1 | 7 | 10 | 7353490 | - | 0.019292 | 4k bp upstream of start | Pcmt1 | protein-L-isoaspartate (D-aspartate) |
| CAAAGCAGCCCGGACGC | 7 | 1 | 7 | 8 | 111593479 | - | 0.019292 | 10k bp upstream of start | Atbf1 | AT motif binding factor 1 |
| CGCGCTCCCGCCCCTCC | 7 | 1 | 7 | 2 | 181522175 | + | 0.019292 | 2k bp downstream of stop | Samd10 | sterile alpha motif domain containing 10 |
| CGCGCTCCCGCCCCTCC | 7 | 1 | 7 | 2 | 181522175 | + | 0.019292 |  | Prpf6 | U5 snRNP-associated 102 kDa protein |
| CGCGCTCCCGCCCCTCC | 7 | 1 | 7 | 2 | 181522175 | + | 0.019292 |  | Uck11 | uridine-cytidine kinase 1-like 1 |
| TCCCTATGTGGGTACCT | 7 | 1 | 7 | 2 | 118594615 | - | 0.019292 | 1k bp downstream of start | Bahd1 | bromo adjacent homology domain containing 1 |
| TCTCCCCAAGACCTCGC | 7 | 1 | 7 | 7 | 114205995 | + | 0.019292 | 467 bp upstream of start | Pde3b | "phosphodiesterase 3B, cGMP-inhibited |
| GTAGATGATCTGTTCCC | 7 | 1 | 7 | 9 | 20726841 | + | 0.019292 | 10k bp upstream of start | Edg5 | "endothelial differentiation, sphingolipid G-protein-coupled receptor, 5 |
| GGTGTCACCAGCCATCT | 7 | 1 | 7 | 2 | 50969874 | - | 0.019292 | 17k bp upstream of start | Rnd3 | Rho family GTPase 3 |
| ATCACACAGACCTTTTG | 6 | 1 | 6 | 13 | 46739837 | - | 0.036266 | 23k bp downstream of stop | Nup153 | nucleoporin1 53 |
| ATCACACAGACCTTTTG | 6 | 1 | 6 | 13 | 46739837 | - | 0.036266 | 23k bp downstream of stop | Kif13a | kinesin family member 13A |
| CGTATTCCCGTAGACCC | 6 | 1 | 6 | 4 | 134424851 | + | 0.036266 | 32k bp upstream of stop | Runx3 | runt related transcription factor 3 (Cpg island in the center of the gene) |
| GAGTACGCTGCGAAGGC | 6 | 1 | 6 | 5 | 123275857 | - | 0.036266 | 166 bp downstream of start | Tmem142a | transmembrane protein 142A |
| AGTGGCCACAGTGAGGC | 6 | 1 | 6 | 15 | 78671291 | - | 0.036266 | 1k bp downstream of start | Cdc42ep1 | CDC42 effector protein (Rho GTPase binding) 1 |
| CATCCTATCAGATCACC | 6 | 1 | 6 | X | 13467876 | - | 0.036266 |  |  | tag sequence surrounded by repeat elements |
| GCAGCTGCGGTGCAGCT | 6 | 1 | 6 | 6 | 47978066 | - | 0.036266 |  | BC066046 | Mus musculus RIKEN cDNA 2500002G23 gene, mRNA (cDNA clone IMAGE:6813195). |
| CAGCTGGCCTCGGCGCA | 6 | 1 | 6 | 15 | 93423692 | + | 0.036266 | 96k bp upstream of start | Prickle1 | prickle like 1 (Drosophila) |
| CCGGCCGCCGCGCCGTT | 6 | 1 | 6 | 19 | 47307020 | - | 0.036266 | 13k bp downstream of stop | Sh3pxd2a | SH3 and PX domains 2A |
| CCGGCCGCCGCGCCGTT | 6 | 1 | 6 | 19 | 47307020 | - | 0.036266 |  | NeurL | neuralized homolog |
| CGGAGTGGACAGGGAGG | 6 | 1 | 6 | 11 | 49745130 | + | 0.036266 | 126 bp upstream of start | Rasgef1c | "RasGEF domain family, member 1C |
| CGGCGTCGCGCGCGGCC | 6 | 1 | 6 | 5 | 118430960 | + | 0.036266 | 166 bp downstream of start | Hrk | "harakiri, BCL2 interacting protein (contains only BH3 domain) |
| ATCTCTGAGAAACAGCC | 6 | 1 | 6 | 5 | 28401520 | + | 0.036266 | 634 bp upstream of start | Insig1 | insulin induced gene 1 |
| GTTAATTCCCAAGTGCA | 6 | 1 | 6 | 4 | 33360710 | - | 0.036266 | 672 bp downstream of start | Ube2j1 | "ubiquitin-conjugating enzyme E2, J1 |
| TGGCTGATTGCCTGTTC | 6 | 1 | 6 | 13 | 84696543 | - | 0.036266 | 1k bp downstream of start | Tmem161b | Hypothetical protein LOC72745 |
| TGTAGGGACACTTTGTG | 6 | 1 | 6 | 7 | 65249206 | + | 0.036266 | 72k bp upstream of start | Tjp1 | tight junction protein 1 |
| AACCGAAGAGGTGAGCC | 6 | 1 | 6 | 13 | 46940532 | - | 0.036266 | 51k bp upstream of start | Kif13a | kinesin family member 13A |
| GGACAAGCTGCACTCCC | 6 | 1 | 6 | 1 | 158078808 | - | 0.036266 | 17k bp downstream of stop | Tdrd5 | tudor domain containing 5 (no CpG island) |
| GCGCAGGCGACCCGGGG | 6 | 1 | 6 | 5 | 117618653 | - | 0.036266 | 318 bp downstream of start | Wsb2 | WD repeat and SOCS box-containing 2 |
| GCGCGCCGGCTGGGGGC | 6 | 1 | 6 | 11 | 102986958 | - | 0.036266 | 445 bp upstream of start | Fmnl1 | lymphocyte specific formin related protein |
| GCGGATGGCCCCAGGTG | 6 | 1 | 6 | 18 | 5591911 | + | 0.036266 | 39 bp downstream of start | Zfhx1a | zinc finger homeobox 1a |
| GCGGCGCCTGCTGCTCC | 6 | 1 | 6 | 6 | 140586083 | - | 0.036266 | 1k bp downstream of start | Aebp2 | AE binding protein 2 isoform 1 |
| GGACAGAGGGGCGTGGA | 6 | 1 | 6 | 15 | 73576164 | - | 0.036266 | 11k bp downstream of stop | Ptp4a3 | protein tyrosine phosphatase 4a3 |
| GGCCGGGCCCGGCGCTT | 6 | 1 | 6 | 7 | 24190862 | + | 0.036266 | 3 bp downstream of start | Igsf4c | "immunoglobulin superfamily, member 4C |
| GGGAGGGGCAGTAGGCG | 6 | 1 | 6 | 4 | 141511597 | - | 0.036266 | 137k bp upstream of start | 9030409G11Rik | "RIKEN cDNA 9030409G11 gene (9030409G11Rik), mRNA |
| GGGCCACCCGCAGTGCA | 6 | 1 | 6 | 2 | 149521559 | + | 0.036266 |  |  | no gene |
| GTAACCGGCACAGAAGA | 6 | 1 | 6 | 1 | 36491167 | + | 0.036266 |  | Ankrd39 | ankyrin repeat domain 39 |
