# Supplementary material for: Evaluating genome-wide DNA methylation changes in mice by Methylation Specific Digital Karyotyping
Source: BMC Genomics. 2008 Dec 11;9:598. doi: 10.1186/1471-2164-9-598 (PMC2621211; doi:10.1186/1471-2164-9-598)
Supplement: Additional file 2 — Differentially methylated genomic tags and nearest genes. Catalogs the 71 genomic sequence tags with higher counts in the HMD MSDK library representing differentially methylated loci within the mouse genome. Also indicated are the nearest genes. [file 1471-2164-9-598-S2.doc]

| Genomic Tag Sequence | LMD | HMD | Ratio | Chr | Position | Orientation | p value | location of tag relative to nearest gene | Gene symbol | Gene description |
| --- | --- | --- | --- | --- | --- | --- | --- | --- | --- | --- |
| TAGACCACTTGCCCCCC | 2 | 27 | 13.5 | 17 | 87975115 | - | 0.000021 |  | unknown |  |
| GACCCACCATTGCAGCA | 2 | 22 | 11.0 | 17 | 29616993 | - | 0.000194 | 94k bp downstream of stop (same strand) | Mdga1 | Synthetic construct Mus musculus MGC:190999 MAM (Mdga1) |
| TAACACAAACCTTTTTG | 2 | 19 | 9.5 | 19 | 60946316 | - | 0.000734 | 1k bp downstream of start (opposite strand) | Gprk5 | G protein-coupled receptor kinase 5 |
| GTCCAAAAAACAGCTAG | 2 | 19 | 9.5 | 10 | 4521324 | + | 0.000734 | 1k bp upstream of start (same strand) | Mtrf1l | mitochondrial translational release factor 1-like |
| CTCTTGTAAGACAAGAG | 1 | 16 | 16.0 | 10 | 39993001 | - | 0.000840 | 14k bp upstream of start (same strand) | Amd1 | S-adenosylmethionine decarboxylase 1 |
| GTGATGCAGGAACTTGG | 4 | 22 | 5.5 | 2 | 91256947 | + | 0.001589 | 1k bp upstream of start (same strand) | Lrp4 | low density lipoprotein receptor-related protein 4 |
| GAAAAAAGATAAATCCC | 1 | 14 | 14.0 | 2 | 70365615 | - | 0.002101 | 2k bp downstream of start (opposite strand) | Gad1 | glutamic acid decarboxylase 1 |
| AGATCCTGTCTCCGGGG | 1 | 14 | 14.0 | 5 | 135678111 | + | 0.002101 | 14k bp downstream of stop (opposite strand) | Pom121 | nuclear pore membrane protein 121 |
| CATAGCTGGAGGACTTG | 1 | 13 | 13.0 | 2 | 27336522 | + | 0.003330 | 66k bp upstream of start (opposite strand) | Brd3 | bromodomain containing 3 |
| TATTAAACAGATGGGCA | 1 | 13 | 13.0 | 2 | 119492229 | - | 0.003330 | 3k bp downstream of start (opposite strand) | Tyro3 | TYRO3 protein tyrosine kinase 3 |
| AACTCACTGCGCTGGGT | 0 | 10 | 10.0 | 12 | 86967165 | + | 0.003347 |  | Tgfb3 | transforming growth factor beta 3 |
| CCCCCCAACACACACAC | 3 | 17 | 5.7 | 11 | 69186327 | - | 0.005048 | 6k bp downstream of stop (opposite strand) | Chd3 | chromodomain helicase DNA binding protein 3 |
| AAACCACCACCTCCTCC | 2 | 14 | 7.0 | 11 | 97516049 | + | 0.006760 | 3k bp upstream of start (same strand) | Pcgf2 | polycomb group ring finger 2 |
| GAGGGGCATAGCCGGTG | 1 | 11 | 11.0 | 15 | 100882640 | - | 0.008413 |  | Scn8a | sodium channel voltage-gated type VIII alpha |
| CGTATTCTGGCCTGTCC | 0 | 8 | 8.0 | 16 | 84717714 | + | 0.008686 | 2 bp upstream of start (same strand) | Gabpa | GA repeat binding protein, alpha |
| GCCCCTTAGGAAGAGAC | 0 | 8 | 8.0 | 2 | 148368284 | + | 0.008686 | 4k bp downstream of stop (same strand) | Nxt1 | NTF2-related export protein 1 |
| TAATATGTACAGACAAG | 0 | 8 | 8.0 | 13 | 99992399 | - | 0.008686 |  | Tnpo1 | transportin 1 isoform 2 |
| CCCTTGATCCCAGCACT | 3 | 15 | 5.0 | 7 | 128313554 | + | 0.011796 | 1k bp upstream of start (same strand) | Bag3 | Bcl2-associated athanogene 3 |
| AAAGCTTTTCCAAAGAG | 1 | 10 | 10.0 | 12 | 112023790 | - | 0.013420 | 1k bp downstream of start (opposite strand) | Mark3 | MAP/microtubule affinity-regulating kinase 3 |
| GTGTTGTGGCCGAAGGT | 1 | 10 | 10.0 | 18 | 58004791 | - | 0.013420 | 744 bp downstream of start (opposite strand) | Slc12a2 | solute carrier family 12, member 2 |
| CCAGCACACCTTCTGCC | 0 | 7 | 7.0 | 10 | 58209135 | + | 0.014101 |  | Sh3md4 | Sh3md4 SH3 multiple domains 4 |
| ATATTCTGTTGTGGGGC | 0 | 7 | 7.0 | 1 | 89438632 | + | 0.014101 |  | Neu2 | neuroaminidase 2 |
| TTTGTTGGAGCTGCCGC | 0 | 7 | 7.0 | 9 | 25002918 | - | 0.014101 | 717 bp downstream of start (opposite strand) | 7-Sep | septin 7 |
| GCACCTTTCGGGACGAG | 0 | 7 | 7.0 | 17 | 25865997 | + | 0.014101 | 337 bp upstream of start (same strand) | Axin1 | axin 1 |
| AATCCCATCGGGCCGGG | 0 | 7 | 7.0 | 8 | 73527707 | - | 0.014101 | 1k bp upstream of stop (same strand) | Ssbp4 | single stranded DNA binding protein 4 |
| AGGGGTCAGAAGGTCCG | 0 | 7 | 7.0 | 8 | 27220122 | - | 0.014101 |  | Ddhd2 | partial Ddhd2 protein |
| AAGGTCTCTACCGCGCC | 0 | 7 | 7.0 | 14 | 61714665 | + | 0.014101 |  | Inst6 | intergrator complex subunit 6 |
| GTCTTTGGTTTGGTCCG | 2 | 12 | 6.0 | 11 | 84340372 | + | 0.016419 | 6k bp upstream of start (opposite strand) | Lhx1 | LIM homeobox protein 1 |
| AATGACTAGGGACTTGC | 0 | 6 | 6.0 | 5 | 75859017 | - | 0.023052 | 2k bp downstream of start (opposite strand) | Kit | kit oncogene |
| ACAGAAATGACAGTGAC | 0 | 6 | 6.0 | 12 | 45140053 | - | 0.023052 |  | Pnpla8 | intracellular membrane-associated |
| AGCGGCGCGGAGAGCGG | 0 | 6 | 6.0 | 16 | 31583992 | + | 0.023052 | 802 bp downstream of start (same strand) | Dlg1 | discs, large homolog 1 (Drosophila) |
| CAGAAGGGAGGGTAGAA | 0 | 6 | 6.0 | 18 | 77064418 | - | 0.023052 |  | Corl2 | Hypothetical protein LOC639628 (CpG Island in middel of gene) |
| GGGAGGGAGGGAAGAGG | 0 | 6 | 6.0 | 2 | 91985078 | - | 0.023052 | 37k bp upstream of start (opposite strand) | Phf21a | PHD finger protein 21A |
| GTGTCGGGCCCCTCCGC | 0 | 6 | 6.0 | X | 96023440 | - | 0.023052 |  | EG620592 | predicted gene EG620592 |
| GAGGATAGAAGGCAGAT | 0 | 6 | 6.0 | 16 | 90272460 | + | 0.023052 | 2k bp upstream of start (same strand) | Hunk | hormonally upregulated Neu-associated kinase |
| GCATCCCTCAGGGAGCA | 0 | 6 | 6.0 | 17 | 83540819 | - | 0.023052 | 46k bp upstream of start (same strand) | Kcng3 | potassium voltage-gated channel, subfamily G, member 3 |
| CCTGCGACGTCCGAGGG | 2 | 11 | 5.5 | 9 | 108549843 | - | 0.025570 | 296 bp downstream of start (opposite strand) | Prkar2a | protein kinase, cAMP dependent regulatory, type II alpha |
| CAGAGACTTTTTTTTGG | 1 | 8 | 8.0 | 11 | 72613105 | - | 0.034448 | 613 bp downstream of start (opposite strand) | Zzef1 | zinc finger, ZZ-type with EF hand domain 1 |
| CGGATCTTTTGCACAGC | 1 | 8 | 8.0 | 3 | 121255311 | - | 0.034448 |  | Tmem56 | transmembrane protein 56 |
| GTGGCCCGGGGTCGTCT | 1 | 8 | 8.0 | 6 | 23789653 | - | 0.034448 | 129k bp downstream of stop (same strand) | Cadps2 | Ca2+ dependent activator for secretion protein |
| TCTAGCAACTACAACAG | 1 | 8 | 8.0 | 3 | 152149000 | - | 0.034448 | 1k bp downstream of start (opposite strand) | Fubp1 | far upstream element (FUSE) binding protein 1 |
| GATGGGTAAGTGCCGGG | 1 | 8 | 8.0 | 4 | 59643190 | - | 0.034448 |  | Rod1 | ROD1 regulator of differentiation 1 isoform 2 |
| AGAAAACCAGAAAAAGG | 1 | 8 | 8.0 | 13 | 23563296 | - | 0.034448 | 797 bp downstream of start (opposite strand) | Hist1h1d | histone 1, H1d |
| CCCGGTGTTTATTTTTG | 1 | 8 | 8.0 | 2 | 103829424 | - | 0.034448 | 786 bp downstream of start (opposite strand) | Fbxo3 | F-box only protein 3 |
| AGCAGCGAGGCCGAGAC | 0 | 5 | 5.0 | 4 | 118792276 | - | 0.038029 |  | Ybx1 | nuclease sensitive element binding protein1 |
| ATGTTCCCGGCACCGCC | 0 | 5 | 5.0 | 3 | 67061733 | - | 0.038029 | 545 bp upstream of stop (same strand) | Shox2 | short stature homeobox 2 |
| AAGTGTCATTGAAATTC | 0 | 5 | 5.0 | 19 | 45728784 | + | 0.038029 | 61k bp downstream of stop (opposite strand) | Fgf8 | fibroblast growth factor 8 |
| TGCGGGACCCTAGAGGT | 0 | 5 | 5.0 | 7 | 82742634 | + | 0.038029 | 899 bp upstream of start (same strand) | Rkhd3 | Rkhd3 protein fragment |
| TTTCTCGTTTGGTACCA | 0 | 5 | 5.0 | 15 | 76025895 | - | 0.038029 | 27k bp upstream of stop (same strand) | Plec1 | plectin 1 |
| TCTGGGTTTGCACCCCA | 0 | 5 | 5.0 | 6 | 37228788 | + | 0.038029 |  | Dgki | diacylglycerol kinase iota |
| TTCCCTGTTCACTGTTC | 0 | 5 | 5.0 | 18 | 4636819 | - | 0.038029 |  | 9430020K01Rik | Hypothetical protein LOC240185 |
| TTCTGTTTATAAAGTTT | 0 | 5 | 5.0 | 9 | 78235279 | - | 0.038029 | 1k bp downstream of start (opposite strand) | Mto1 | mitochondrial translation optimization 1 homolog (S. cerevisiae) |
| AAGTTTCTGATCCCCAC | 0 | 5 | 5.0 | 14 | 69371660 | - | 0.038029 | 375 bp upstream of stop (same strand) | Fgf17 | fibroblast growth factor 17 |
| ACCCCCTCACCTGGCCC | 0 | 5 | 5.0 | 18 | 81149843 | - | 0.038029 | 21k bp upstream of start (same strand) | Sall3 | sal-like 3 (Drosophila) |
| GCATCTAAGGCTAAGAA | 0 | 5 | 5.0 | 7 | 133547704 | + | 0.038029 | 24 bp downstream of start (same strand) | Bccip | BRCA2 and CDKN1A interacting protein |
| GCCACCTCCAGCGCGCC | 0 | 5 | 5.0 | 2 | 32534667 | + | 0.038029 | 5k bp upstream of start (opposite strand) | Cdk9 | cyclin-dependent kinase 9 (CDC2-related kinase) |
| GAGCTGGAAGGAGATCA | 0 | 5 | 5.0 | X | 147484477 | + | 0.038029 | 35k bp upstream of start (same strand) | Iqsec2 | IQ motif and Sec7 domain 2 |
| GAAGAACAGCAGCCGGA | 0 | 5 | 5.0 | 1 | 122430582 | + | 0.038029 | 355 bp downstream of start (same strand) | En1 | engrailed 1 |
| CCACAACACAGACAGAC | 0 | 5 | 5.0 | 10 | 69491907 | + | 0.038029 |  | Ccdc6 | coiled-coil domain containing 6 |
| CAAATCTCTGCATCGTT | 0 | 5 | 5.0 | 5 | 118237539 | + | 0.038029 | 261 bp upstream of start (same strand) | Fbxo21 | F-box only protein 21 |
| GGGAAACGGGATAATCG | 0 | 5 | 5.0 | 5 | 3549933 | + | 0.038029 | 3 bp downstream of start (same strand) | 5830415L20Rik | hypothetical protein LOC68152 |
| GGCACGCCTGGGCGCTG | 0 | 5 | 5.0 | 14 | 67079068 | - | 0.038029 | 6k bp upstream of start (same strand) | Nef3 | neurofilament 3, medium |
| GGAAAGGTGGTTTTATG | 0 | 5 | 5.0 | 14 | 69370646 | + | 0.038029 | 639 bp downstream of stop (opposite strand) | Fgf17 | fibroblast growth factor 17 |
| CCAGCTCTCGCCATAAA | 0 | 5 | 5.0 | 10 | 82766871 | + | 0.038029 |  | Slc41a2 | solute carrier family 41 member2 |
| CCAGGGCACGCCCGCCG | 0 | 5 | 5.0 | 15 | 98924572 | + | 0.038029 | 2k bp upstream of start (same strand) | 2810451A06Rik | RIKEN cDNA 2810451A06 |
| GCTACCTTTGCTAGCCC | 0 | 5 | 5.0 | 9 | 106084174 | + | 0.038029 | 2k bp upstream of stop (same strand) | Tlr9 | toll-like receptor 9 |
| GCGACGCGGGCCTCGAG | 0 | 5 | 5.0 | 2 | 143961870 | + | 0.038029 |  | Snx5 | sorting nexin 5 |
| CACCCGGAGGGCTGCAG | 2 | 10 | 5.0 | 10 | 28836156 | + | 0.039783 | 81 bp downstream of start (same strand) | 6330407J23Rik | hypothetical protein LOC67412 |
| AACAAAAAGGGTCTGTG | 2 | 10 | 5.0 | 17 | 31115354 | - | 0.039783 | 393 bp downstream of start (opposite strand) | Pde9a | phosphodiesterase 9A |
| CGGTCAAGAGGGAGGGA | 2 | 10 | 5.0 | 12 | 81031989 | + | 0.039783 | 5k bp upstream of start (opposite strand) | Zfp36l1 | zinc finger protein 36, C3H type-like 1 |
| TGGGATGCTGGAAGTAC | 2 | 10 | 5.0 | 5 | 37156069 | + | 0.039783 | 750 bp upstream of start (same strand) | Ppp2r2c | protein phosphatase 2 (formerly 2A), regulatory subunit B (PR 52) |
|  |  |  |  |  |  |  |  |  |  |  |
