# Supplementary material for: Evaluating genome-wide DNA methylation changes in mice by Methylation Specific Digital Karyotyping
Source: BMC Genomics. 2008 Dec 11;9:598. doi: 10.1186/1471-2164-9-598 (PMC2621211; doi:10.1186/1471-2164-9-598)
Supplement: Additional file 3 — Differentially expressed SAGE transcripts. Represents the transcript tags significantly over expressed in the HMD SAGE library. [file 1471-2164-9-598-S3.doc]

| ID | Tag Sequence | LMD SAGE | HMD SAGE | Ratio HvsL | P value | Gene Symbol | Gene Description |
| --- | --- | --- | --- | --- | --- | --- | --- |
| 1 | GAGCAGCGTG | 1 | 17 | 17.0 | 0.00129 | Sppl3 | Signal peptide peptidase 3 |
| 2 | TGTTGTGTTG | 1 | 17 | 17.0 | 0.00129 | Slc26a4 | Solute carrier family 26, member 4 |
| 3 | TTGACTCTTA | 1 | 15 | 15.0 | 0.00350 | Ptk2 | PTK2 protein tyrosine kinase 2 |
| 4 | ACTTAAAAAA | 0 | 14 | 14.0 | 0.00081 | Nfe2l2 | Nuclear factor, erythroid derived 2, like 2 |
| 5 | ATCATAGTCT | 1 | 14 | 14.0 | 0.00496 |  |  |
| 6 | TGAATGGCCT | 1 | 14 | 14.0 | 0.00496 | Klhdc2 | Kelch domain containing 2 |
| 7 | TTTAAGAATG | 1 | 14 | 14.0 | 0.00496 | 0910001A06Rik | RIKEN cDNA 0910001A06 gene |
| 8 | ATTATATTTT | 1 | 13 | 13.0 | 0.00759 |  | ATPase, H+ transporting, lysosomal V1 subunit B2 |
| 9 | TGAAGAGAGA | 1 | 13 | 13.0 | 0.00759 | Ap3d1 | Adaptor-related protein complex 3, delta 1 subunit |
| 10 | AAGCAGGAGA | 1 | 12 | 12.0 | 0.01110 | Tmcc2 | Transmembrane and coiled-coil domains 2 |
| 11 | AGTCAGATTT | 1 | 12 | 12.0 | 0.01110 |  |  |
| 12 | CTGCCCCCCG | 1 | 12 | 12.0 | 0.01110 | Saa3 | Serum amyloid A 3 |
| 13 | GAAAGCAATG | 1 | 12 | 12.0 | 0.01110 | Ngfrap1 | Nerve growth factor receptor (TNFRSF16) associated protein 1 |
| 14 | AAGAAGACTT | 1 | 11 | 11.0 | 0.01873 | Gabarap | Gamma-aminobutyric acid receptor associated protein |
| 15 | AATGATAAAA | 1 | 11 | 11.0 | 0.01873 |  | Transcribed locus, moderately similar to XP_916581.2 PREDICTED: similar to Nol5a protein |
| 16 | CATACGCTCA | 1 | 11 | 11.0 | 0.01873 |  | Transcribed locus |
| 17 | CTAAGTGACA | 1 | 11 | 11.0 | 0.01873 | Gbp6 | Guanylate binding protein 6 |
| 18 | CTTTTGATAC | 1 | 11 | 11.0 | 0.01873 | Rapgef2 | Rap guanine nucleotide exchange factor (GEF) 2 |
| 19 | GCTTTCTTAC | 1 | 11 | 11.0 | 0.01873 |  | Transcribed locus, strongly similar to NP_904337.1 NADH dehydrogenase subunit 4 [Mus musculus] |
| 20 | TGAAATAGGA | 1 | 11 | 11.0 | 0.01873 | Dcbld2 | Discoidin, CUB and LCCL domain containing 2 |
| 21 | CAAATTTGTC | 2 | 21 | 10.5 | 0.00098 |  |  |
| 22 | ATTTTATTCT | 0 | 10 | 10.0 | 0.00600 | Dhx57 | DEAH (Asp-Glu-Ala-Asp/His) box polypeptide 57 |
| 23 | GGGGTTTTCC | 0 | 10 | 10.0 | 0.00600 |  |  |
| 24 | TGGTTGTCTA | 0 | 10 | 10.0 | 0.00600 |  |  |
| 25 | AATATCACCT | 1 | 10 | 10.0 | 0.02677 |  | Transcribed locus, strongly similar to NP_904330.1 cytochrome c oxidase subunit I |
| 26 | ATATGTCAGG | 1 | 10 | 10.0 | 0.02677 |  |  |
| 27 | GTCTGTGCCC | 1 | 10 | 10.0 | 0.02677 | Chmp4b | Chromatin modifying protein 4B |
| 28 | TTTAGCTGAC | 1 | 10 | 10.0 | 0.02677 | Zfp706 | Zinc finger protein 706 |
| 29 | TTTCAAGTGG | 1 | 10 | 10.0 | 0.02677 |  |  |
| 30 | TTTTGTATCA | 1 | 10 | 10.0 | 0.02677 |  |  |
| 31 | TGCCTTTAAA | 4 | 39 | 9.7 | 0.00001 | Retnla | Resistin like alpha |
| 32 | GGGAGGGAGT | 0 | 9 | 9.0 | 0.01008 | 0610025P10Rik | RIKEN cDNA 0610025P10 gene |
| 33 | GGGTTCTGAC | 0 | 9 | 9.0 | 0.01008 | Eif2c3 | Eukaryotic translation initiation factor 2C, 3 |
| 34 | TCCGCAGACA | 0 | 9 | 9.0 | 0.01008 | Mynn | Myoneurin |
| 35 | TCGAAAGACC | 0 | 9 | 9.0 | 0.01008 |  |  |
| 36 | AAATAGACAT | 1 | 9 | 9.0 | 0.04115 | 0610031J06Rik | RIKEN cDNA 0610031J06 gene |
| 37 | ATGTCTCAAG | 1 | 9 | 9.0 | 0.04115 |  |  |
| 38 | CACTCTGGAA | 1 | 9 | 9.0 | 0.04115 | Wdr42a | WD repeat domain 42A |
| 39 | GCGGCTCACC | 1 | 9 | 9.0 | 0.04115 | Lsm4 | LSM4 homolog, U6 small nuclear RNA associated |
| 40 | GGGGGTGAGG | 1 | 9 | 9.0 | 0.04115 |  | Histidine acid phosphatase domain containing 1 |
| 41 | TAACAAAGTA | 1 | 9 | 9.0 | 0.04115 | Ston1 | Stonin 1 |
| 42 | TAGGGTATGA | 1 | 9 | 9.0 | 0.04115 | B4galt4 | UDP-Gal:betaGlcNAc beta 1,4-galactosyltransferase, polypeptide 4 |
| 43 | TCACTGTACT | 1 | 9 | 9.0 | 0.04115 | Magi3 | Membrane associated guanylate kinase, WW and PDZ domain containing 3 |
| 44 | TCTTGAAATT | 1 | 9 | 9.0 | 0.04115 | Gbp2 | Guanylate nucleotide binding protein 2 |
| 45 | TTTATGGAAT | 1 | 9 | 9.0 | 0.04115 | Map2k4 | Mitogen-activated protein kinase kinase 4 |
| 46 | TGAAAAAAAA | 36 | 295 | 8.1 | 0.00000 | Alkbh4 | AlkB, alkylation repair homolog 4 (E. coli) |
| 47 | AAAAAATAAA | 0 | 8 | 8.0 | 0.01566 | Rnase9 | Ribonuclease, RNase A family, 9 (non-active) |
| 48 | AATGGCAGGG | 0 | 8 | 8.0 | 0.01566 | Cyb5b | Cytochrome b5 type B |
| 49 | AGCGTGGTGG | 0 | 8 | 8.0 | 0.01566 | Plekhm2 | Pleckstrin homology domain containing, family M (with RUN domain) member 2 |
| 50 | ATGCATTTCT | 0 | 8 | 8.0 | 0.01566 | Megf9 | Multiple EGF-like-domains 9 |
| 51 | GCTGGAATAA | 0 | 8 | 8.0 | 0.01566 | AU014645 | Expressed sequence AU014645 |
| 52 | GGTTTGGAAT | 0 | 8 | 8.0 | 0.01566 | C78226 | Myosin, heavy polypeptide 10, non-muscle |
| 53 | TTTCAAATAA | 0 | 8 | 8.0 | 0.01566 | Tacc2 | Transforming, acidic coiled-coil containing protein 2 |
| 54 | AAAATAAAAA | 2 | 15 | 7.5 | 0.01217 | Cfl2 | Cofilin 2, muscle |
| 55 | TATTTATTTA | 2 | 14 | 7.0 | 0.01617 | Gtf3c1 | General transcription factor III C 1 |
| 56 | TTCAGTAACA | 2 | 14 | 7.0 | 0.01617 | LOC553150 | Expressed sequence AA407270 |
| 57 | ATATTGTACC | 0 | 7 | 7.0 | 0.02663 | Dynlrb2 | Dynein light chain roadblock-type 2 |
| 58 | ATGGTTGTAG | 0 | 7 | 7.0 | 0.02663 |  |  |
| 59 | CAGGAGCATC | 0 | 7 | 7.0 | 0.02663 |  | Transcribed locus, strongly similar to NP_904330.1 cytochrome c oxidase subunit I |
| 60 | CCCCACAAAA | 0 | 7 | 7.0 | 0.02663 | Yipf5 | Yip1 domain family, member 5 |
| 61 | CCTTTAATCT | 0 | 7 | 7.0 | 0.02663 | Xpo5 | Exportin 5 |
| 62 | GCTCCCACAC | 0 | 7 | 7.0 | 0.02663 | Tnni3 | Troponin I, cardiac |
| 63 | TAACTGGATT | 0 | 7 | 7.0 | 0.02663 | Hps5 | Hermansky-Pudlak syndrome 5 homolog (human) |
| 64 | TATGTATTTG | 0 | 7 | 7.0 | 0.02663 | Rnf44 | Ring finger protein 44 |
| 65 | TATTATTTGG | 0 | 7 | 7.0 | 0.02663 | LOC100039918 | RIKEN cDNA 3321401G04 gene |
| 66 | TGACCAAGTA | 0 | 7 | 7.0 | 0.02663 |  |  |
| 67 | AGGAGATGGA | 2 | 13 | 6.5 | 0.02656 | Ctbp2 | Zinc finger, RAN-binding domain containing 1 |
| 68 | CGCTGATAGG | 2 | 13 | 6.5 | 0.02656 | Kctd2 | Potassium channel tetramerisation domain containing 2 |
| 69 | GAAATAAAGT | 2 | 13 | 6.5 | 0.02656 |  | RIKEN cDNA 1110012J17 gene |
| 70 | TAACTTAAGC | 2 | 13 | 6.5 | 0.02656 | Ypel5 | Yippee-like 5 (Drosophila) |
| 71 | ATTGGCCCCA | 2 | 12 | 6.0 | 0.04121 | Plekhb1 | Pleckstrin homology domain containing, family B (evectins) member 1 |
| 72 | ATTTCTTTGG | 2 | 12 | 6.0 | 0.04121 |  | Microtubule-associated protein tau |
| 73 | CCGAAAGTAA | 2 | 12 | 6.0 | 0.04121 | Sdc2 | Syndecan 2 |
| 74 | CCTTTTCTTT | 2 | 12 | 6.0 | 0.04121 | Ghitm | Growth hormone inducible transmembrane protein |
| 75 | GATAAAGCAA | 2 | 12 | 6.0 | 0.04121 | Cgnl1 | Cingulin-like 1 |
| 76 | GGAAAAAAAA | 2 | 12 | 6.0 | 0.04121 | Tspan33 | Tetraspanin 33 |
| 77 | TACTACTTTG | 2 | 12 | 6.0 | 0.04121 | Srebf1 | Sterol regulatory element binding transcription factor 1 |
| 78 | TCAGAGTGAG | 2 | 12 | 6.0 | 0.04121 | Igh-V3609N | Immunoglobulin heavy chain complex |
| 79 | TGCAATAAAG | 2 | 12 | 6.0 | 0.04121 | Cd69 | CD69 antigen |
| 80 | TGCACTATTG | 2 | 12 | 6.0 | 0.04121 | 1500012F01Rik | RIKEN cDNA 1500012F01 gene |
| 81 | TGCTGCCAGA | 2 | 12 | 6.0 | 0.04121 | Ogfod2 | 2-oxoglutarate and iron-dependent oxygenase domain containing 2 |
| 82 | TGGGAAGTGT | 2 | 12 | 6.0 | 0.04121 | Vat1 | Vesicle amine transport protein 1 homolog (T californica) |
| 83 | AAGCTCTCGG | 0 | 6 | 6.0 | 0.04697 |  |  |
| 84 | AATAATGATA | 0 | 6 | 6.0 | 0.04697 |  |  |
| 85 | ACATTTCAAT | 0 | 6 | 6.0 | 0.04697 | Gabarapl1 | Gamma-aminobutyric acid (GABA(A)) receptor-associated protein-like 1 |
| 86 | ACCAGGGCCT | 0 | 6 | 6.0 | 0.04697 | Actr1b | ARP1 actin-related protein 1 homolog B (yeast) |
| 87 | ACCCCAATCA | 0 | 6 | 6.0 | 0.04697 | Nfs1 | Nitrogen fixation gene 1 (S. cerevisiae) |
| 88 | ACTGATTGCA | 0 | 6 | 6.0 | 0.04697 |  | Phospholipase A2, activating protein |
| 89 | AGAGAGAGAG | 0 | 6 | 6.0 | 0.04697 | Csnk1e | Casein kinase 1, epsilon |
| 90 | ATACTGGCAT | 0 | 6 | 6.0 | 0.04697 |  | CDNA clone IMAGE:40049146 |
| 91 | ATGGGCAGGC | 0 | 6 | 6.0 | 0.04697 | Pmvk | Phosphomevalonate kinase |
| 92 | CTACAATCAG | 0 | 6 | 6.0 | 0.04697 | Nfkb1 | Nuclear factor of kappa light polypeptide gene enhancer in B-cells 1, p105 |
| 93 | CTGTATACTT | 0 | 6 | 6.0 | 0.04697 |  | Transcribed locus |
| 94 | GAAAAATAAA | 0 | 6 | 6.0 | 0.04697 | Eif1ad | Eukaryotic translation initiation factor 1A domain containing |
| 95 | GAAGAAGGTA | 0 | 6 | 6.0 | 0.04697 | Bcar3 | Breast cancer anti-estrogen resistance 3 |
| 96 | GAATTTTATT | 0 | 6 | 6.0 | 0.04697 | Eftud2 | Elongation factor Tu GTP binding domain containing 2 |
| 97 | GGAAGAAGAA | 0 | 6 | 6.0 | 0.04697 |  | Tudor domain containing 3 |
| 98 | GGCATCCCAT | 0 | 6 | 6.0 | 0.04697 | Scgb3a1 | Secretoglobin, family 3A, member 1 |
| 99 | GGTTAATGTT | 0 | 6 | 6.0 | 0.04697 | Pja1 | Praja1, RING-H2 motif containing |
| 100 | TAAATTCCAC | 0 | 6 | 6.0 | 0.04697 | Pde9a | Phosphodiesterase 9A |
| 101 | TAATAAATGA | 0 | 6 | 6.0 | 0.04697 | Deb1 | Differentially expressed in B16F10 1 |
| 102 | TACCTAAGGT | 0 | 6 | 6.0 | 0.04697 | ENSMUSG00000073776 | Coiled-coil and C2 domain containing 1B |
| 103 | TGAGTCCAAT | 0 | 6 | 6.0 | 0.04697 | Iws1 | IWS1 homolog (S. cerevisiae) |
| 104 | TGATTACTAG | 0 | 6 | 6.0 | 0.04697 |  |  |
| 105 | TGTAACATTA | 0 | 6 | 6.0 | 0.04697 |  | Transcribed locus |
| 106 | TGTAATGTAA | 0 | 6 | 6.0 | 0.04697 | Klf11 | Kruppel-like factor 11 |
| 107 | TTTAAAAAAA | 0 | 6 | 6.0 | 0.04697 | Ren2 | Renin 2 tandem duplication of Ren1 |
| 108 | TTTCCTTAGG | 0 | 6 | 6.0 | 0.04697 |  | Phosphatidylinositol transfer protein, beta |
| 109 | TTTTATTATT | 0 | 6 | 6.0 | 0.04697 | Ugcg | UDP-glucose ceramide glucosyltransferase |
| 110 | TTTTATTCTC | 0 | 6 | 6.0 | 0.04697 | Capn12 | Calpain 12 |
| 111 | TGTAAAAAAA | 7 | 41 | 5.8 | 0.00010 |  | Transcribed locus |
| 112 | AAACACTGTC | 4 | 20 | 5.0 | 0.01429 |  | Transcribed locus, strongly similar to NP_904330.1 cytochrome c oxidase subunit I [Mus musculus] |
| 113 | CTCGAATAAA | 4 | 19 | 4.7 | 0.01793 | Zeb2 | Zinc finger E-box binding homeobox 2 |
| 114 | AACCTAATGC | 7 | 33 | 4.7 | 0.00187 | Chia | Chitinase, acidic |
| 115 | TGCTGAATCA | 4 | 18 | 4.5 | 0.02580 | AU015740 | Coiled-coil domain containing 6 |
| 116 | ACCGTTCTGT | 5 | 22 | 4.4 | 0.01503 | Tcf4 | Transcription factor 4 |
| 117 | ACCTCAACTA | 4 | 17 | 4.2 | 0.03513 | Gstt1 | Glutathione S-transferase, theta 1 |
| 118 | TGTTTAGTTC | 4 | 17 | 4.3 | 0.03513 | Klc1 | Kinesin light chain 1 |
| 119 | TTTATTATTT | 5 | 21 | 4.2 | 0.02055 | Ncald | Neurocalcin delta |
| 120 | CTAAGACTTC | 7 | 28 | 4.0 | 0.00994 |  |  |
| 121 | CTGTAGGTGC | 5 | 20 | 4.0 | 0.03048 | BC020535 | CDNA sequence BC020535 |
| 122 | GAAAGCACTC | 5 | 20 | 4.0 | 0.03048 |  | Transcribed locus, moderately similar to XP_216565.2 PREDICTED: similar to PTEN induced putative kinase 1 [Rattus norvegicus] |
| 123 | TTTGTTAAAC | 5 | 20 | 4.0 | 0.03048 | Tmem176a | Transmembrane protein 176A |
| 124 | TTTTAAAATA | 5 | 20 | 4.0 | 0.03048 | Ttyh3 | Tweety homolog 3 (Drosophila) |
| 125 | TTAAAAAAAA | 7 | 27 | 3.9 | 0.01460 |  | NADH dehydrogenase (ubiquinone) Fe-S protein 1 |
| 126 | CTCCTAATCT | 10 | 38 | 3.8 | 0.00410 | Snrp70 | U1 small nuclear ribonucleoprotein polypeptide A |
| 127 | AGAATTTAAT | 5 | 19 | 3.8 | 0.04248 | Sema3c | Sema domain, immunoglobulin domain (Ig), short basic domain, secreted, (semaphorin) 3C |
| 128 | ATAACTGAGT | 5 | 19 | 3.8 | 0.04248 |  | Bromodomain containing 8 |
| 129 | TTCAGAGAAT | 5 | 19 | 3.8 | 0.04248 | Mmp15 | Matrix metallopeptidase 15 |
| 130 | GTTTTTTAAA | 11 | 40 | 3.6 | 0.00446 | Rpn1 | Ribophorin I |
| 131 | TCATCTCAGA | 8 | 29 | 3.6 | 0.01686 | Golga4 | Golgi autoantigen, golgin subfamily a, 4 |
| 132 | TAAATAAAGA | 10 | 33 | 3.3 | 0.01643 | LOC100043003 | Bromodomain containing 2 |
| 133 | AGTCCTGGAT | 8 | 26 | 3.3 | 0.03104 |  | High mobility group box 1 |
| 134 | TGTCAAAAAA | 10 | 31 | 3.1 | 0.02634 |  |  |
| 135 | ACCCTCCTCC | 29 | 87 | 3.0 | 0.00046 |  |  |
| 136 | TAAAAAAAAA | 29 | 71 | 2.5 | 0.01421 | Qdpr | Quinoid dihydropteridine reductase |
| 137 | TGTAAAATAA | 23 | 54 | 2.5 | 0.04192 |  | Potassium channel tetramerisation domain containing 10 |
| 138 | CTTGTTTCTT | 58 | 123 | 2.1 | 0.01657 | Chi3l3 | Chitinase 3-like 3 |
| 139 | TAGATATAGG | 126 | 258 | 2.1 | 0.00212 |  | CDNA clone IMAGE:40049146 |
