# Supplementary material for: Evaluating genome-wide DNA methylation changes in mice by Methylation Specific Digital Karyotyping
Source: BMC Genomics. 2008 Dec 11;9:598. doi: 10.1186/1471-2164-9-598 (PMC2621211; doi:10.1186/1471-2164-9-598)
Supplement: Additional file 4 — Differentially expressed SAGE transcripts. Represents the transcript tags significantly over expressed in the LMD SAGE library. [file 1471-2164-9-598-S4.doc]

| ID | Tag Sequence | LMD SAGE | HMD SAGE | Ratio LvsH | P value | Gene Symbol | Gene Description |
| --- | --- | --- | --- | --- | --- | --- | --- |
| 1 | ACCCGGCTAG | 15 | 0 | 15.0 | 0.00000 | Sept4 | Septin 4 |
| 2 | CATCTGTTAA | 15 | 0 | 15.0 | 0.00000 | Spon1 | Spondin 1, (f-spondin) extracellular matrix protein |
| 3 | GCTTTCCTGT | 13 | 1 | 13.0 | 0.00007 | Col4a1 | Collagen, type IV, alpha 1 |
| 4 | AATATGGCCG | 11 | 0 | 11.0 | 0.00003 |  |  |
| 5 | AGCCTACAGA | 11 | 0 | 11.0 | 0.00003 | Eif5a | Eukaryotic translation initiation factor 5A |
| 6 | CTTTCTCCTT | 11 | 0 | 11.0 | 0.00003 |  |  |
| 7 | GGAAATGCAG | 11 | 0 | 11.0 | 0.00003 | Ddx5 | DEAD (Asp-Glu-Ala-Asp) box polypeptide 5 |
| 8 | GTGGTGGTTT | 11 | 0 | 11.0 | 0.00003 | Malat1 | Metastasis associated lung adenocarcinoma transcript 1 (non-coding RNA) |
| 9 | TCATCTCGCG | 11 | 0 | 11.0 | 0.00003 | Cib1 | Calcium and integrin binding 1 (calmyrin) |
| 10 | AACATTTTGG | 11 | 1 | 11.0 | 0.00035 | Il10rb | Interleukin 10 receptor, beta |
| 11 | AAGGACAGCA | 11 | 1 | 11.0 | 0.00035 | Cd97 | CD97 antigen |
| 12 | AGCAGGTTTT | 11 | 1 | 11.0 | 0.00035 |  |  |
| 13 | ATGCAGGGCC | 11 | 1 | 11.0 | 0.00035 | Fasn | Fatty acid synthase |
| 14 | ATTTCCTTTG | 11 | 1 | 11.0 | 0.00035 | Mgat2 | Transcribed locus |
| 15 | CCCTGCACAG | 11 | 1 | 11.0 | 0.00035 | Cnpy2 | Canopy 2 homolog (zebrafish) |
| 16 | GCCCCAGGAG | 11 | 1 | 11.0 | 0.00035 | Nucb1 | Nucleobindin 1 |
| 17 | TTGTGCTTAT | 11 | 1 | 11.0 | 0.00035 |  | WW, C2 and coiled-coil domain containing 2 |
| 18 | AATATGCCTT | 10 | 0 | 10.0 | 0.00011 | Zfp36l2 | Zinc finger protein 36, C3H type-like 2 |
| 19 | AGTGTTTTCT | 10 | 0 | 10.0 | 0.00011 | Xlr5a | X-linked lymphocyte-regulated 5A |
| 20 | ATAAAATGTG | 10 | 0 | 10.0 | 0.00011 |  | Transcribed locus |
| 21 | CAAAAATAGT | 10 | 0 | 10.0 | 0.00011 | Sult1a1 | Sulfotransferase family 1A, phenol-preferring, member 1 |
| 22 | GAACACTTCT | 10 | 0 | 10.0 | 0.00011 | Stx7 | Syntaxin 7 |
| 23 | GATATTTTTC | 10 | 0 | 10.0 | 0.00011 |  |  |
| 24 | GTCTGCGTGC | 10 | 0 | 10.0 | 0.00011 | Psma1 | Proteasome (prosome, macropain) subunit, alpha type 1 |
| 25 | GTGTTTGAAA | 10 | 0 | 10.0 | 0.00011 | Cltb | Clathrin, light polypeptide (Lcb) |
| 26 | TCTGACTTCC | 10 | 0 | 10.0 | 0.00011 | Bgn | Biglycan |
| 27 | AATTTGGTCG | 10 | 1 | 10.0 | 0.00082 |  |  |
| 28 | ACCTCTCAGA | 10 | 1 | 10.0 | 0.00082 | Nup85 | Nucleoporin 85 |
| 29 | CCTTTGACTT | 10 | 1 | 10.0 | 0.00082 |  |  |
| 30 | CGGGCTCCAC | 10 | 1 | 10.0 | 0.00082 | Pccb | Propionyl Coenzyme A carboxylase, beta polypeptide |
| 31 | CTGAGCTGCA | 10 | 1 | 10.0 | 0.00082 | Wdr6 | WD repeat domain 6 |
| 32 | GCCCAGCCTT | 10 | 1 | 10.0 | 0.00082 | Ncaph2 | Non-SMC condensin II complex, subunit H2 |
| 33 | GCTGTCAGCC | 10 | 1 | 10.0 | 0.00082 | Lgals9 | Lectin, galactose binding, soluble 9 |
| 34 | GGTCACACTA | 10 | 1 | 10.0 | 0.00082 | Uqcrfs1 | Ubiquinol-cytochrome c reductase, Rieske iron-sulfur polypeptide 1 |
| 35 | TCTTGAATGT | 10 | 1 | 10.0 | 0.00082 |  |  |
| 36 | TGTAATCATA | 10 | 1 | 10.0 | 0.00082 |  | Forkhead box N3 |
| 37 | TTTTATTTTT | 10 | 1 | 10.0 | 0.00082 | D130076G13Rik | PAP associated domain containing 4 |
| 38 | TGGATCTGAG | 36 | 4 | 9.0 | 0.00000 |  | Transcribed locus |
| 39 | TTCTGGCTGC | 17 | 2 | 8.5 | 0.00001 | Uqcrc1 | Ubiquinol-cytochrome c reductase core protein 1 |
| 40 | AAAATGAACC | 8 | 0 | 8.0 | 0.00066 |  | RIKEN cDNA 1810022K09 gene |
| 41 | AAGCCGCAGG | 8 | 0 | 8.0 | 0.00066 | Fbl | Fibrillarin |
| 42 | ATGGCAATTT | 8 | 0 | 8.0 | 0.00066 | Cfl2 | Cofilin 2, muscle |
| 43 | ATGTCTGTTT | 8 | 0 | 8.0 | 0.00066 |  |  |
| 44 | CAGAACAAGT | 8 | 0 | 8.0 | 0.00066 | Meox2 | Mesenchyme homeobox 2 |
| 45 | CTAACTGAGA | 8 | 0 | 8.0 | 0.00066 | Cobra1 | Cofactor of BRCA1 |
| 46 | GAATTTTCCT | 8 | 0 | 8.0 | 0.00066 | Lamc1 | Laminin, gamma 1 |
| 47 | GAGGGTAAAG | 8 | 0 | 8.0 | 0.00066 | Ctso | Cathepsin O |
| 48 | GATGATGATG | 8 | 0 | 8.0 | 0.00066 | Josd3 | Josephin domain containing 3 |
| 49 | GGAGGGTTGG | 8 | 0 | 8.0 | 0.00066 | Arl8a | ADP-ribosylation factor-like 8A |
| 50 | GGCTCAGGAA | 8 | 0 | 8.0 | 0.00066 |  |  |
| 51 | GGCTGGAGAT | 8 | 0 | 8.0 | 0.00066 | Thbd | Thrombomodulin |
| 52 | GTGGAAGAAT | 8 | 0 | 8.0 | 0.00066 | Atp1b1 | ATPase, Na+/K+ transporting, beta 1 polypeptide |
| 53 | TGACTGAGGT | 8 | 0 | 8.0 | 0.00066 | Sra1 | Steroid receptor RNA activator 1 |
| 54 | TGTTCACTAT | 8 | 0 | 8.0 | 0.00066 | Shisa5 | Shisa homolog 5 (Xenopus laevis) |
| 55 | TTGCAACAGG | 8 | 0 | 8.0 | 0.00066 | Eltd1 | EGF, latrophilin seven transmembrane domain containing 1 |
| 56 | AAGGTCTATT | 8 | 1 | 8.0 | 0.00405 | Tbcel | Tubulin folding cofactor E-like |
| 57 | AAGTTGAGTA | 8 | 1 | 8.0 | 0.00405 |  | Transcribed locus, strongly similar to NP_081435.1 coiled-coil domain containing 115 [Mus musculus] |
| 58 | AAGTTTGCAA | 8 | 1 | 8.0 | 0.00405 | Marcks | Myristoylated alanine rich protein kinase C substrate |
| 59 | AGTTGCTTCT | 8 | 1 | 8.0 | 0.00405 |  |  |
| 60 | ATAAGAAGAG | 8 | 1 | 8.0 | 0.00405 | Myo1b | Myosin IB |
| 61 | CAAAATACTG | 8 | 1 | 8.0 | 0.00405 | Vezf1 | Vascular endothelial zinc finger 1 |
| 62 | CACACACACA | 8 | 1 | 8.0 | 0.00405 | Zfyve26 | Zinc finger, FYVE domain containing 26 |
| 63 | CAGAGCCACC | 8 | 1 | 8.0 | 0.00405 |  | Myelin basic protein |
| 64 | CAGGCGTCCC | 8 | 1 | 8.0 | 0.00405 | Rdm1 | RAD52 motif 1 |
| 65 | CGATTTGAAA | 8 | 1 | 8.0 | 0.00405 |  |  |
| 66 | CGCGACAAAC | 8 | 1 | 8.0 | 0.00405 | Acoxl | Acyl-Coenzyme A oxidase-like |
| 67 | CTCTTCCCCC | 8 | 1 | 8.0 | 0.00405 |  | Phosphatidylethanolamine binding protein 1 |
| 68 | CTGCTAGCAC | 8 | 1 | 8.0 | 0.00405 |  |  |
| 69 | CTTGTTTAGT | 8 | 1 | 8.0 | 0.00405 | 1110004F10Rik | RIKEN cDNA 1110004F10 gene |
| 70 | GAAGGAGGAC | 8 | 1 | 8.0 | 0.00405 | Ssb | Sjogren syndrome antigen B |
| 71 | GCCACACTTG | 8 | 1 | 8.0 | 0.00405 |  | CDNA clone IMAGE:6834410 |
| 72 | GCTCTAGTCT | 8 | 1 | 8.0 | 0.00405 |  |  |
| 73 | GGCACAGCTG | 8 | 1 | 8.0 | 0.00405 | Rps6ka4 | Ribosomal protein S6 kinase, polypeptide 4 |
| 74 | GGCAGTGCCC | 8 | 1 | 8.0 | 0.00405 | Plxnd1 | Plexin D1 |
| 75 | TAAGGAACAA | 8 | 1 | 8.0 | 0.00405 | Sec13 | SEC13 homolog (S. cerevisiae) |
| 76 | TCCCCCTCAA | 8 | 1 | 8.0 | 0.00405 |  | CDNA clone IMAGE:30292875 |
| 77 | TCCGAACAAA | 8 | 1 | 8.0 | 0.00405 | Nudt9 | Nudix (nucleoside diphosphate linked moiety X)-type motif 9 |
| 78 | TTAATTGTAT | 8 | 1 | 8.0 | 0.00405 | Phca | Phytoceramidase, alkaline |
| 79 | TGGATCCTGA | 407 | 53 | 7.7 | 0.00000 | Hbb-b1 | Hemoglobin, beta adult minor chain |
| 80 | AGCAGAGCCC | 15 | 2 | 7.5 | 0.00005 |  | Solute carrier family 35, member B4 |
| 81 | TCGCCTACTG | 15 | 2 | 7.5 | 0.00005 | Yif1b | Yip1 interacting factor homolog B (S. cerevisiae) |
| 82 | CAAGTTTCCA | 14 | 2 | 7.0 | 0.00010 |  |  |
| 83 | CAGGAGGAGT | 14 | 2 | 7.0 | 0.00010 | Pdia3 | Protein disulfide isomerase associated 3 |
| 84 | CCATTGATCA | 14 | 2 | 7.0 | 0.00010 | Dynlrb1 | Dynein light chain roadblock-type 1 |
| 85 | TGCCCTCGCC | 14 | 2 | 7.0 | 0.00010 | 0610010K14Rik | RIKEN cDNA 0610010K14 gene |
| 86 | AAGCAGAGCT | 7 | 0 | 7.0 | 0.00170 |  | G protein-coupled receptor, family C, group 5, member A |
| 87 | AAGCCTCACG | 7 | 0 | 7.0 | 0.00170 |  |  |
| 88 | AGCAAGCAAG | 7 | 0 | 7.0 | 0.00170 | 669460 | Hypothetical LOC669460 |
| 89 | AGCAGTGGCT | 7 | 0 | 7.0 | 0.00170 | C2cd2l | C2 calcium-dependent domain containing 2-like |
| 90 | AGCCAAGGGT | 7 | 0 | 7.0 | 0.00170 | Adamts13 | A disintegrin-like and metallopeptidase (reprolysin type) with thrombospondin type 1 motif, 13 |
| 91 | AGCCCAGCCA | 7 | 0 | 7.0 | 0.00170 |  | RIKEN cDNA 1700106N22 gene |
| 92 | AGGATGCTTG | 7 | 0 | 7.0 | 0.00170 |  |  |
| 93 | AGGGTGGCGG | 7 | 0 | 7.0 | 0.00170 |  |  |
| 94 | AGTACAATGA | 7 | 0 | 7.0 | 0.00170 | 2900010J23Rik | RIKEN cDNA 2900010J23 gene |
| 95 | ATAACTGGTT | 7 | 0 | 7.0 | 0.00170 |  |  |
| 96 | ATACAATAAA | 7 | 0 | 7.0 | 0.00170 | Nutf2 | Nuclear transport factor 2 |
| 97 | ATAGCAGAGG | 7 | 0 | 7.0 | 0.00170 |  | Transcribed locus |
| 98 | ATCGGTTCCA | 7 | 0 | 7.0 | 0.00170 | Bxdc1 | Brix domain containing 1 |
| 99 | ATCTGTTTAA | 7 | 0 | 7.0 | 0.00170 |  |  |
| 100 | ATGATGGTAG | 7 | 0 | 7.0 | 0.00170 |  | Transcribed locus, weakly similar to XP_001003727.1 PREDICTED: similar to RNP particle component [Mus musculus] |
| 101 | ATGTGCTTCC | 7 | 0 | 7.0 | 0.00170 |  |  |
| 102 | ATTGGGGTGG | 7 | 0 | 7.0 | 0.00170 | Dynll2 | Dynein light chain LC8-type 2 |
| 103 | CAGCAGAGCA | 7 | 0 | 7.0 | 0.00170 |  |  |
| 104 | CAGCTGCCTC | 7 | 0 | 7.0 | 0.00170 | Cidec | Cell death-inducing DFFA-like effector c |
| 105 | CAGCTGGCCA | 7 | 0 | 7.0 | 0.00170 | Fbln1 | Fibulin 1 |
| 106 | CAGCTTCCCT | 7 | 0 | 7.0 | 0.00170 | 2210411K11Rik | RIKEN cDNA 2210411K11 gene |
| 107 | CCAGGGGAAT | 7 | 0 | 7.0 | 0.00170 |  | Bromodomain and PHD finger containing, 1 |
| 108 | CTCCACAGAA | 7 | 0 | 7.0 | 0.00170 | Cx3cl1 | Chemokine (C-X3-C motif) ligand 1 |
| 109 | CTCTCCCTAT | 7 | 0 | 7.0 | 0.00170 | Cd200 | Cd200 antigen |
| 110 | CTGATGCCAT | 7 | 0 | 7.0 | 0.00170 |  | Eukaryotic translation elongation factor 2 |
| 111 | CTGGTTGGAG | 7 | 0 | 7.0 | 0.00170 |  | Lectin, mannose-binding, 1 |
| 112 | GACTACAGTA | 7 | 0 | 7.0 | 0.00170 | Nagk | N-acetylglucosamine kinase |
| 113 | GACTGCCCCT | 7 | 0 | 7.0 | 0.00170 | Dek | DEK oncogene (DNA binding) |
| 114 | GAGCTCCAGC | 7 | 0 | 7.0 | 0.00170 | Eif4ebp1 | Eukaryotic translation initiation factor 4E binding protein 1 |
| 115 | GAGCTGCTCA | 7 | 0 | 7.0 | 0.00170 |  |  |
| 116 | GCCCACACAT | 7 | 0 | 7.0 | 0.00170 | Rtp4 | Receptor transporter protein 4 |
| 117 | GGAAACCACG | 7 | 0 | 7.0 | 0.00170 |  | T-complex protein 1 |
| 118 | GGATTCAACA | 7 | 0 | 7.0 | 0.00170 | Pip5k1a | Phosphatidylinositol-4-phosphate 5-kinase, type 1 alpha |
| 119 | GGCCTGAATT | 7 | 0 | 7.0 | 0.00170 | Tor2a | Torsin family 2, member A |
| 120 | GGGCCTCCCC | 7 | 0 | 7.0 | 0.00170 | Pak4 | P21 (CDKN1A)-activated kinase 4 |
| 121 | GGGGTCATAC | 7 | 0 | 7.0 | 0.00170 | Per3 | Period homolog 3 (Drosophila) |
| 122 | GGTTGATTCT | 7 | 0 | 7.0 | 0.00170 | Cav2 | Caveolin 2 |
| 123 | GTATACAGAC | 7 | 0 | 7.0 | 0.00170 | Mrps7 | Mitchondrial ribosomal protein S7 |
| 124 | GTCCTACTCC | 7 | 0 | 7.0 | 0.00170 | Clic3 | Chloride intracellular channel 3 |
| 125 | GTTTGCCTGA | 7 | 0 | 7.0 | 0.00170 |  | Vestigial like 4 (Drosophila) |
| 126 | TAGTCTCTTC | 7 | 0 | 7.0 | 0.00170 |  |  |
| 127 | TCCATTTTGT | 7 | 0 | 7.0 | 0.00170 |  |  |
| 128 | TCTCACAACT | 7 | 0 | 7.0 | 0.00170 |  | Transcribed locus |
| 129 | TGAAAACCTA | 7 | 0 | 7.0 | 0.00170 |  | ADP-ribosylation factor-like 8B |
| 130 | TGACTACATA | 7 | 0 | 7.0 | 0.00170 | 6330403M23Rik | Transcribed locus, strongly similar to NP_001007190.1 hypothetical protein LOC492311 [Homo sapiens] |
| 131 | TGATGTAAAC | 7 | 0 | 7.0 | 0.00170 | Lmo7 | LIM domain only 7 |
| 132 | TGCAAGAAAG | 7 | 0 | 7.0 | 0.00170 | Setd7 | SET domain containing (lysine methyltransferase) 7 |
| 133 | TGCTCTCAAC | 7 | 0 | 7.0 | 0.00170 |  |  |
| 134 | TGGACCTCTG | 7 | 0 | 7.0 | 0.00170 |  |  |
| 135 | TGGGTTCTGA | 7 | 0 | 7.0 | 0.00170 |  | Transcribed locus |
| 136 | TGTCACAAAA | 7 | 0 | 7.0 | 0.00170 | Herc2 | Hect (homologous to the E6-AP (UBE3A) carboxyl terminus) domain and RCC1 (CHC1)-like domain (RLD) 2 |
| 137 | TGTCTGCCTC | 7 | 0 | 7.0 | 0.00170 | Cldn18 | Claudin 18 |
| 138 | TGTGGACAGT | 7 | 0 | 7.0 | 0.00170 | Aldh6a1 | Aldehyde dehydrogenase family 6, subfamily A1 |
| 139 | TGTGTGTCGC | 7 | 0 | 7.0 | 0.00170 | Dynll2 | Dynein light chain LC8-type 2 |
| 140 | TTTCTGAAGG | 7 | 0 | 7.0 | 0.00170 | Lrrc16a | Leucine rich repeat containing 16A |
| 141 | TTTGCTCGAT | 7 | 0 | 7.0 | 0.00170 | Rbm6 | RNA binding motif protein 6 |
| 142 | AAAGATCTGT | 7 | 1 | 7.0 | 0.00902 | Snord22 | Small nucleolar RNA, C/D box 22 |
| 143 | AAATGTATCA | 7 | 1 | 7.0 | 0.00902 |  |  |
| 144 | AAGAAGCCTC | 7 | 1 | 7.0 | 0.00902 | Zkscan17 | Zinc finger with KRAB and SCAN domains 17 |
| 145 | AATGTTCATC | 7 | 1 | 7.0 | 0.00902 | Ms4a4d | Membrane-spanning 4-domains, subfamily A, member 4D |
| 146 | ACAGGACTCA | 7 | 1 | 7.0 | 0.00902 | 1810058I24Rik | Transcribed locus, moderately similar to XP_940679.2 PREDICTED: hypothetical protein [Homo sapiens] |
| 147 | ACGTCGTGCG | 7 | 1 | 7.0 | 0.00902 | Hipk2 | Transcribed locus |
| 148 | AGAAATTTTA | 7 | 1 | 7.0 | 0.00902 | Gpr177 | G protein-coupled receptor 177 |
| 149 | AGACATTGGT | 7 | 1 | 7.0 | 0.00902 | Tmed3 | Transmembrane emp24 domain containing 3 |
| 150 | AGAGCTTGTT | 7 | 1 | 7.0 | 0.00902 | Smpd1 | Sphingomyelin phosphodiesterase 1, acid lysosomal |
| 151 | AGCTCTGTAG | 7 | 1 | 7.0 | 0.00902 |  | Transcribed locus, moderately similar to XP_001713705.1 PREDICTED: hypothetical protein [Homo sapiens] |
| 152 | AGGCCTCCAG | 7 | 1 | 7.0 | 0.00902 | Tap2 | Transporter 2, ATP-binding cassette, sub-family B (MDR/TAP) |
| 153 | ATAAAGTAAC | 7 | 1 | 7.0 | 0.00902 | Strap | Serine/threonine kinase receptor associated protein |
| 154 | ATGCAGCCGC | 7 | 1 | 7.0 | 0.00902 |  |  |
| 155 | ATGCGAGTGA | 7 | 1 | 7.0 | 0.00902 | Foxk2 | Forkhead box K2 |
| 156 | ATTTTAGCAA | 7 | 1 | 7.0 | 0.00902 | Whdc1 | WAS protein homology region 2 domain containing 1 |
| 157 | CAAAAGAATA | 7 | 1 | 7.0 | 0.00902 | Htatsf1 | HIV TAT specific factor 1 |
| 158 | CAACTTCCCG | 7 | 1 | 7.0 | 0.00902 | A130072A22Rik | ELAV (embryonic lethal, abnormal vision, Drosophila)-like 1 (Hu antigen R) |
| 159 | CAATTCAAAT | 7 | 1 | 7.0 | 0.00902 | Tomm20 | Translocase of outer mitochondrial membrane 20 homolog (yeast) |
| 160 | CAGCTCACGG | 7 | 1 | 7.0 | 0.00902 |  |  |
| 161 | CATACGCATA | 7 | 1 | 7.0 | 0.00902 | Ghr | Growth hormone receptor |
| 162 | CCAAAATTAG | 7 | 1 | 7.0 | 0.00902 |  |  |
| 163 | CCAGGGGCCT | 7 | 1 | 7.0 | 0.00902 |  |  |
| 164 | CCTGAGGGGT | 7 | 1 | 7.0 | 0.00902 |  |  |
| 165 | CCTGTCATCC | 7 | 1 | 7.0 | 0.00902 | Mier1 | Mesoderm induction early response 1 homolog (Xenopus laevis |
| 166 | CGTGTGGGCT | 7 | 1 | 7.0 | 0.00902 | Pgd | Phosphogluconate dehydrogenase |
| 167 | CTAATTAAGA | 7 | 1 | 7.0 | 0.00902 | Brap | BRCA1 associated protein |
| 168 | CTGTGGAATG | 7 | 1 | 7.0 | 0.00902 | Pnpla2 | Patatin-like phospholipase domain containing 2 |
| 169 | CTTAAACTGC | 7 | 1 | 7.0 | 0.00902 | Stim2 | Stromal interaction molecule 2 |
| 170 | GAACACACAG | 7 | 1 | 7.0 | 0.00902 |  | Transcribed locus |
| 171 | GAAGAGGGGA | 7 | 1 | 7.0 | 0.00902 | ENSMUSG00000073566 | Transcribed locus |
| 172 | GAAGCACCCT | 7 | 1 | 7.0 | 0.00902 | Lims2 | LIM and senescent cell antigen like domains 2 |
| 173 | GAAGCCTGTG | 7 | 1 | 7.0 | 0.00902 | 1110018F16Rik | Transcribed locus |
| 174 | GACTTCCTGA | 7 | 1 | 7.0 | 0.00902 | Tst | Thiosulfate sulfurtransferase, mitochondrial |
| 175 | GAGCCCTATA | 7 | 1 | 7.0 | 0.00902 | Haghl | Hydroxyacylglutathione hydrolase-like |
| 176 | GAGTGGACTT | 7 | 1 | 7.0 | 0.00902 | Ptgs2 | Prostaglandin-endoperoxide synthase 2 |
| 177 | GATGTGGTAC | 7 | 1 | 7.0 | 0.00902 | Ppib | Peptidylprolyl isomerase B |
| 178 | GCCTTCTTCT | 7 | 1 | 7.0 | 0.00902 | Olfr1308 | Olfactory receptor 1308 |
| 179 | GCTTTGACTG | 7 | 1 | 7.0 | 0.00902 |  | Transcribed locus |
| 180 | GGTGGAACGG | 7 | 1 | 7.0 | 0.00902 | Alg3 | Asparagine-linked glycosylation 3 homolog (yeast, alpha-1,3-mannosyltransferase) |
| 181 | GGTTTATGCT | 7 | 1 | 7.0 | 0.00902 | D14Ertd668e | DNA segment, Chr 14, ERATO Doi 668, expressed |
| 182 | GTGCATCCAG | 7 | 1 | 7.0 | 0.00902 | Csnk2b | Casein kinase 2, beta polypeptide |
| 183 | TATCACTCTG | 7 | 1 | 7.0 | 0.00902 | Mea1 | Male enhanced antigen 1 |
| 184 | TCTCTCTGCT | 7 | 1 | 7.0 | 0.00902 | Rcor1 | REST corepressor 1 |
| 185 | TTCAAATTTT | 7 | 1 | 7.0 | 0.00902 | Eif4e3 | Eukaryotic translation initiation factor 4E member 3 |
| 186 | TTCTTGACTT | 7 | 1 | 7.0 | 0.00902 | Palm | Paralemmin |
| 187 | TTTCTTTTTG | 7 | 1 | 7.0 | 0.00902 | Txndc10 | Thioredoxin domain containing 10 |
| 188 | TTTGCAGCCA | 7 | 1 | 7.0 | 0.00902 | Bckdha | Branched chain ketoacid dehydrogenase E1, alpha polypeptide |
| 189 | TTTGGTATTG | 7 | 1 | 7.0 | 0.00902 | LOC100038890 | Trans-golgi network protein |
| 190 | GAGCCTGTAA | 13 | 2 | 6.5 | 0.00030 |  | Transcribed locus |
| 191 | TCAGTGCACA | 13 | 2 | 6.5 | 0.00030 |  |  |
| 192 | AGTCAAAAAC | 69 | 12 | 5.8 | 0.00000 | Sftpc | Surfactant associated protein C |
| 193 | AGAGTCAGCG | 17 | 3 | 5.7 | 0.00005 | Atp6v0e | ATPase, H+ transporting, lysosomal V0 subunit E |
| 194 | TTGTGTGATG | 17 | 3 | 5.7 | 0.00005 | Mageh1 | Melanoma antigen, family H, 1 |
| 195 | AAGCTCGAAA | 11 | 2 | 5.5 | 0.00118 | Sdhd | Succinate dehydrogenase complex, subunit D, integral membrane protein |
| 196 | CGCTGTACAG | 11 | 2 | 5.5 | 0.00118 | Igfbp3 | Insulin-like growth factor binding protein 3 |
| 197 | CTGCACAGTG | 11 | 2 | 5.5 | 0.00118 |  | Eukaryotic translation elongation factor 2 |
| 198 | TCAAAGGGTT | 11 | 2 | 5.5 | 0.00118 |  |  |
| 199 | TCTGTGTATG | 11 | 2 | 5.5 | 0.00118 | Irx3 | Iroquois related homeobox 3 (Drosophila) |
| 200 | TCATTATTGA | 20 | 4 | 5.0 | 0.00002 | LOC100038890 | Trans-golgi network protein |
| 201 | GGCAAAGAGG | 15 | 3 | 5.0 | 0.00021 | Hc | Hemolytic complement |
| 202 | TGCTCAGATA | 15 | 3 | 5.0 | 0.00021 | Sod3 | Superoxide dismutase 3, extracellular |
| 203 | TGCTGGACGT | 15 | 3 | 5.0 | 0.00021 | Srebf2 | Sterol regulatory element binding factor 2 |
| 204 | AATCTGGAAA | 10 | 2 | 5.0 | 0.00281 | Pigq | Phosphatidylinositol glycan anchor biosynthesis, class Q |
| 205 | AATGGCCCTT | 10 | 2 | 5.0 | 0.00281 | Xbp1 | X-box binding protein 1 |
| 206 | ACTTATTATG | 10 | 2 | 5.0 | 0.00281 | Dcn | Decorin |
| 207 | CACTGTCCCT | 10 | 2 | 5.0 | 0.00281 | D0H4S114 | DNA segment, human D4S114 |
| 208 | CTGAGTAACA | 10 | 2 | 5.0 | 0.00281 |  |  |
| 209 | CTGCGAGATT | 10 | 2 | 5.0 | 0.00281 | Ndufv2 | NADH dehydrogenase (ubiquinone) flavoprotein 2 |
| 210 | GAACCCTTCT | 10 | 2 | 5.0 | 0.00281 |  |  |
| 211 | GGAATGTCAA | 10 | 2 | 5.0 | 0.00281 | Myd88 | Myeloid differentiation primary response gene 88 |
| 212 | GGCAATAATG | 10 | 2 | 5.0 | 0.00281 | Idh1 | Isocitrate dehydrogenase 1 (NADP+), soluble |
| 213 | GTTTTGGGAG | 10 | 2 | 5.0 | 0.00281 | Arfgef1 | ADP-ribosylation factor guanine nucleotide-exchange factor 1(brefeldin A-inhibited) |
| 214 | TAAGCTGTGA | 10 | 2 | 5.0 | 0.00281 | Katna1 | Katanin p60 (ATPase-containing) subunit A1 |
| 215 | TGGGTTCCTT | 10 | 2 | 5.0 | 0.00281 | Limch1 | LIM and calponin homology domains 1 |
| 216 | TTTGGTTATA | 10 | 2 | 5.0 | 0.00281 | Ndufc2 | NADH dehydrogenase (ubiquinone) 1, subcomplex unknown, 2 |
| 217 | AAAATTGCAG | 5 | 0 | 5.0 | 0.01040 | 9530046B11Rik | Transcribed locus |
| 218 | AAATTATATG | 5 | 0 | 5.0 | 0.01040 | Sec23b | SEC23B (S. cerevisiae) |
| 219 | AACTCTGTCA | 5 | 0 | 5.0 | 0.01040 | Dnahc2 | Dynein, axonemal, heavy chain 2 |
| 220 | AACTGCACAC | 5 | 0 | 5.0 | 0.01040 | Sdhb | Succinate dehydrogenase complex, subunit B, iron sulfur (Ip) |
| 221 | AACTGGGTCT | 5 | 0 | 5.0 | 0.01040 | Arl3 | ADP-ribosylation factor-like 3 |
| 222 | AACTTGGCTG | 5 | 0 | 5.0 | 0.01040 |  | RIKEN cDNA 4833439L19 gene |
| 223 | AAGGTGCTGG | 5 | 0 | 5.0 | 0.01040 | 1190007F08Rik | RIKEN cDNA 1190007F08 gene |
| 224 | AAGGTGGGTG | 5 | 0 | 5.0 | 0.01040 | D4Ucla1 | Cell division cycle 42 homolog (S. cerevisiae) |
| 225 | AAGTACAAAA | 5 | 0 | 5.0 | 0.01040 | Scaf1 | SR-related CTD-associated factor 1 |
| 226 | AATGGCTTTG | 5 | 0 | 5.0 | 0.01040 |  | Transcribed locus |
| 227 | ACCACAGTGA | 5 | 0 | 5.0 | 0.01040 |  |  |
| 228 | ACCCATCCAG | 5 | 0 | 5.0 | 0.01040 | Rshl2a | Radial spokehead-like 2A |
| 229 | AGACGCAAAG | 5 | 0 | 5.0 | 0.01040 | Bcam | Basal cell adhesion molecule |
| 230 | AGCCTCACGG | 5 | 0 | 5.0 | 0.01040 |  |  |
| 231 | AGGAAGGAAG | 5 | 0 | 5.0 | 0.01040 | Cacnb1 | Calcium channel, voltage-dependent, beta 1 subunit |
| 232 | AGGAATCCAC | 5 | 0 | 5.0 | 0.01040 | Gas1 | Growth arrest specific 1 |
| 233 | AGGGAGCTAC | 5 | 0 | 5.0 | 0.01040 |  | Prosaposin |
| 234 | ATATAAAAGA | 5 | 0 | 5.0 | 0.01040 | Igkv14-111 | Immunoglobulin kappa chain variable 14-111 |
| 235 | ATATTAGCAA | 5 | 0 | 5.0 | 0.01040 |  |  |
| 236 | ATCAGTGTGA | 5 | 0 | 5.0 | 0.01040 |  |  |
| 237 | ATCTGTCCAT | 5 | 0 | 5.0 | 0.01040 | Slc25a40 | Solute carrier family 25, member 40 |
| 238 | ATGAGTCATA | 5 | 0 | 5.0 | 0.01040 |  | NMD3 homolog (S. cerevisiae) |
| 239 | ATGCTCACGT | 5 | 0 | 5.0 | 0.01040 |  |  |
| 240 | ATTCACACTG | 5 | 0 | 5.0 | 0.01040 | Olfr908 | Olfactory receptor 908 |
| 241 | ATTCCTTTTC | 5 | 0 | 5.0 | 0.01040 | Fmo3 | Flavin containing monooxygenase 3 |
| 242 | ATTTCGTCAA | 5 | 0 | 5.0 | 0.01040 |  | Transcribed locus |
| 243 | ATTTGTTGGG | 5 | 0 | 5.0 | 0.01040 |  | F-box protein 28 |
| 244 | CAAACACCGT | 5 | 0 | 5.0 | 0.01040 | Spp1 | Secreted phosphoprotein 1 |
| 245 | CAAATTAACC | 5 | 0 | 5.0 | 0.01040 | Ccna2 | Cyclin A2 |
| 246 | CAATAAAGTC | 5 | 0 | 5.0 | 0.01040 |  |  |
| 247 | CACACAGACT | 5 | 0 | 5.0 | 0.01040 |  |  |
| 248 | CACACATACA | 5 | 0 | 5.0 | 0.01040 | 1700028K03Rik | RIKEN cDNA 1700028K03 gene |
| 249 | CACAGTTGTG | 5 | 0 | 5.0 | 0.01040 | Gtl3 | Gene trap locus 3 |
| 250 | CACATTAATA | 5 | 0 | 5.0 | 0.01040 |  | Signal recognition particle 72 |
| 251 | CACCGAGGTG | 5 | 0 | 5.0 | 0.01040 | Top3b | Topoisomerase (DNA) III beta |
| 252 | CACCGGGGAG | 5 | 0 | 5.0 | 0.01040 |  |  |
| 253 | CACTGTATGG | 5 | 0 | 5.0 | 0.01040 | Zfhx3 | Zinc finger homeobox 3 |
| 254 | CAGACTAATT | 5 | 0 | 5.0 | 0.01040 |  |  |
| 255 | CAGATTTAGG | 5 | 0 | 5.0 | 0.01040 |  |  |
| 256 | CAGCCAAATT | 5 | 0 | 5.0 | 0.01040 |  | Nipped-B homolog (Drosophila) |
| 257 | CAGTCTAAAA | 5 | 0 | 5.0 | 0.01040 | Wipi1 | WD repeat domain, phosphoinositide interacting 1 |
| 258 | CAGTGATTTC | 5 | 0 | 5.0 | 0.01040 | Tmed4 | Transmembrane emp24 protein transport domain containing 4 |
| 259 | CAGTGCTTGC | 5 | 0 | 5.0 | 0.01040 |  | Tissue factor pathway inhibitor |
| 260 | CCCTCCTTCT | 5 | 0 | 5.0 | 0.01040 |  | CDNA clone IMAGE:40049146 |
| 261 | CCGAAGCCCA | 5 | 0 | 5.0 | 0.01040 |  |  |
| 262 | CCGGGTCCAA | 5 | 0 | 5.0 | 0.01040 | Sec61b | Sec61 beta subunit |
| 263 | CCTGGAAACG | 5 | 0 | 5.0 | 0.01040 | Cnn1 | Calponin 1 |
| 264 | CCTTCCCTTC | 5 | 0 | 5.0 | 0.01040 | Ubqln4 | Ubiquilin 4 |
| 265 | CCTTGCTCAT | 5 | 0 | 5.0 | 0.01040 |  |  |
| 266 | CTACAAAAAG | 5 | 0 | 5.0 | 0.01040 | Mtch2 | Mitochondrial carrier homolog 2 (C. elegans) |
| 267 | CTAGTCGCAA | 5 | 0 | 5.0 | 0.01040 | 4930423K06Rik | Thymidine phosphorylase |
| 268 | CTCCTAACCC | 5 | 0 | 5.0 | 0.01040 | Rogdi | Rogdi homolog (Drosophila) |
| 269 | CTCTGAGTCT | 5 | 0 | 5.0 | 0.01040 | 1110032E23Rik | RIKEN cDNA 1110032E23 gene |
| 270 | CTCTGTGAAT | 5 | 0 | 5.0 | 0.01040 | Tmem18 | Transmembrane protein 18 |
| 271 | CTGCTTTCAG | 5 | 0 | 5.0 | 0.01040 |  | PR domain containing 2, with ZNF domain |
| 272 | CTGGACACTG | 5 | 0 | 5.0 | 0.01040 | Atp13a2 | ATPase type 13A2 |
| 273 | CTGGAGCCAA | 5 | 0 | 5.0 | 0.01040 | Eef1a1 | Eukaryotic translation elongation factor 1 alpha 1 |
| 274 | CTGTGTGGCC | 5 | 0 | 5.0 | 0.01040 | E130012A19Rik | RIKEN cDNA E130012A19 gene |
| 275 | CTTGACCTGT | 5 | 0 | 5.0 | 0.01040 | Luc7l | Luc7 homolog (S. cerevisiae)-like |
| 276 | CTTGACCTTT | 5 | 0 | 5.0 | 0.01040 | Aldh18a1 | Aldehyde dehydrogenase 18 family, member A1 |
| 277 | CTTGTCAGCT | 5 | 0 | 5.0 | 0.01040 |  |  |
| 278 | GAAAAATTGT | 5 | 0 | 5.0 | 0.01040 |  | Transcribed locus |
| 279 | GAAATCTTTA | 5 | 0 | 5.0 | 0.01040 | Dusp14 | Dual specificity phosphatase 14 |
| 280 | GAACCAGTTA | 5 | 0 | 5.0 | 0.01040 | Diras2 | DIRAS family, GTP-binding RAS-like 2 |
| 281 | GAAGAGGTTT | 5 | 0 | 5.0 | 0.01040 | Mnat1 | Menage a trois 1 |
| 282 | GACGAGTATG | 5 | 0 | 5.0 | 0.01040 | Mrps35 | Mitochondrial ribosomal protein S35 |
| 283 | GACTCAGCTC | 5 | 0 | 5.0 | 0.01040 | Tmem55a | Transmembrane protein 55A |
| 284 | GACTCAGGGA | 5 | 0 | 5.0 | 0.01040 | Plod3 | Procollagen-lysine, 2-oxoglutarate 5-dioxygenase 3 |
| 285 | GACTTGGACA | 5 | 0 | 5.0 | 0.01040 |  | Transcribed locus |
| 286 | GAGGATCTAC | 5 | 0 | 5.0 | 0.01040 | Sept10 | Septin 10 |
| 287 | GAGTCTTCGA | 5 | 0 | 5.0 | 0.01040 |  |  |
| 288 | GAGTGTGCCT | 5 | 0 | 5.0 | 0.01040 | Inmt | Indolethylamine N-methyltransferase |
| 289 | GATGGAGACT | 5 | 0 | 5.0 | 0.01040 | Pskh1 | Protein serine kinase H1 |
| 290 | GCACGAGCTC | 5 | 0 | 5.0 | 0.01040 | Ctdsp1 | CTD (carboxy-terminal domain, RNA polymerase II, polypeptide A) small phosphatase 1 |
| 291 | GCAGCACTCA | 5 | 0 | 5.0 | 0.01040 |  | Transcribed locus |
| 292 | GCAGCATTCT | 5 | 0 | 5.0 | 0.01040 | 2210020M01Rik | RIKEN cDNA 2210020M01 gene |
| 293 | GCCAAGAACT | 5 | 0 | 5.0 | 0.01040 | Ubfd1 | Ubiquitin family domain containing 1 |
| 294 | GCCACCACCT | 5 | 0 | 5.0 | 0.01040 | Muc1 | Mucin 1, transmembrane |
| 295 | GCCTCGGGGG | 5 | 0 | 5.0 | 0.01040 | Mrpl33 | Mitochondrial ribosomal protein L33 |
| 296 | GCGAGCCCCA | 5 | 0 | 5.0 | 0.01040 |  |  |
| 297 | GCTAATCTTC | 5 | 0 | 5.0 | 0.01040 | Tm9sf3 | Transmembrane 9 superfamily member 3 |
| 298 | GCTCCCCCAA | 5 | 0 | 5.0 | 0.01040 | A430104N18Rik | Transcribed locus |
| 299 | GCTCTATACA | 5 | 0 | 5.0 | 0.01040 | Yy1 | Transcribed locus |
| 300 | GCTCTGCCCA | 5 | 0 | 5.0 | 0.01040 | Gabrp | Gamma-aminobutyric acid (GABA-A) receptor, pi |
| 301 | GCTGACTCCG | 5 | 0 | 5.0 | 0.01040 |  |  |
| 302 | GGAGGTGTGC | 5 | 0 | 5.0 | 0.01040 | Lcn8 | Lipocalin 8 |
| 303 | GGATGCATTC | 5 | 0 | 5.0 | 0.01040 |  | CDNA clone IMAGE:5253491 |
| 304 | GGCCCGAGAG | 5 | 0 | 5.0 | 0.01040 |  |  |
| 305 | GGCGACGGGA | 5 | 0 | 5.0 | 0.01040 |  |  |
| 306 | GGCTGGGAGG | 5 | 0 | 5.0 | 0.01040 | 1810022C23Rik | RIKEN cDNA 1810022C23 gene |
| 307 | GGGATATAAA | 5 | 0 | 5.0 | 0.01040 | Dnaja1 | DnaJ (Hsp40) homolog, subfamily A, member 1 |
| 308 | GGGTGGGGGA | 5 | 0 | 5.0 | 0.01040 | D4Ucla1 | Cell division cycle 42 homolog (S. cerevisiae) |
| 309 | GGTGGTCAAT | 5 | 0 | 5.0 | 0.01040 |  |  |
| 310 | GTACCTGCTT | 5 | 0 | 5.0 | 0.01040 |  |  |
| 311 | GTACTGGTTC | 5 | 0 | 5.0 | 0.01040 |  | Transcribed locus |
| 312 | GTCAAAGTTC | 5 | 0 | 5.0 | 0.01040 | Nolc1 | Nucleolar and coiled-body phosphoprotein 1 |
| 313 | GTGAAGAACA | 5 | 0 | 5.0 | 0.01040 | Lepr | Leptin receptor |
| 314 | GTGCTGAATA | 5 | 0 | 5.0 | 0.01040 | Hba-a1 | Hemoglobin alpha, adult chain 1 |
| 315 | GTGGATTTTT | 5 | 0 | 5.0 | 0.01040 | EG623592 | Predicted gene, EG623592 |
| 316 | GTGGGAATAG | 5 | 0 | 5.0 | 0.01040 |  |  |
| 317 | GTTCTCTGTC | 5 | 0 | 5.0 | 0.01040 | 3110001D03Rik | RIKEN cDNA 3110001D03 gene |
| 318 | GTTGTAGAAA | 5 | 0 | 5.0 | 0.01040 | Gtf2e1 | General transcription factor II E, polypeptide 1 (alpha subunit) |
| 319 | TAATAATTAT | 5 | 0 | 5.0 | 0.01040 | Als2cr4 | Amyotrophic lateral sclerosis 2 (juvenile) chromosome region, candidate 4 |
| 320 | TACACACTGG | 5 | 0 | 5.0 | 0.01040 |  | Trafficking protein particle complex 2 |
| 321 | TAGAAAAGAC | 5 | 0 | 5.0 | 0.01040 | Rab21 | RAB21, member RAS oncogene family |
| 322 | TATCTGAGGC | 5 | 0 | 5.0 | 0.01040 | Olfr1019 | Olfactory receptor 1019 |
| 323 | TCAGTGTGCA | 5 | 0 | 5.0 | 0.01040 | Lrg1 | Leucine-rich alpha-2-glycoprotein 1 |
| 324 | TCATCTGGAA | 5 | 0 | 5.0 | 0.01040 | Chfr | Checkpoint with forkhead and ring finger domains |
| 325 | TCTGTGAACT | 5 | 0 | 5.0 | 0.01040 | Il18r1 | Interleukin 18 receptor 1 |
| 326 | TGAACATCTC | 5 | 0 | 5.0 | 0.01040 | Ptprg | Protein tyrosine phosphatase, receptor type, G |
| 327 | TGACACGGGT | 5 | 0 | 5.0 | 0.01040 |  |  |
| 328 | TGGACAGCCT | 5 | 0 | 5.0 | 0.01040 | 1110039B18Rik | RIKEN cDNA 1110039B18 gene |
| 329 | TGGCATACAG | 5 | 0 | 5.0 | 0.01040 | Jarid1a | Jumonji, AT rich interactive domain 1A (Rbp2 like) |
| 330 | TGGGATGCAC | 5 | 0 | 5.0 | 0.01040 | Eif3j | Eukaryotic translation initiation factor 3, subunit J |
| 331 | TGTAAGGTAT | 5 | 0 | 5.0 | 0.01040 | Slc25a24 | Solute carrier family 25 (mitochondrial carrier, phosphate carrier), member 24 |
| 332 | TGTATACAGT | 5 | 0 | 5.0 | 0.01040 | Mmp11 | Matrix metallopeptidase 11 |
| 333 | TGTTGGAGTC | 5 | 0 | 5.0 | 0.01040 |  |  |
| 334 | TTACACAGTT | 5 | 0 | 5.0 | 0.01040 |  |  |
| 335 | TTACATTTAT | 5 | 0 | 5.0 | 0.01040 | Cdkal1 | CDK5 regulatory subunit associated protein 1-like 1 |
| 336 | TTCCAGCTAC | 5 | 0 | 5.0 | 0.01040 | AW549877 | Expressed sequence AW549877 |
| 337 | TTCCCACCTT | 5 | 0 | 5.0 | 0.01040 |  | Transcribed locus |
| 338 | TTCGCAGCAG | 5 | 0 | 5.0 | 0.01040 |  |  |
| 339 | TTCTGGTTTG | 5 | 0 | 5.0 | 0.01040 |  | A disintegrin and metallopeptidase domain 28 |
| 340 | TTGAGTCGGC | 5 | 0 | 5.0 | 0.01040 |  |  |
| 341 | TTGATCATCA | 5 | 0 | 5.0 | 0.01040 | Arhgdib | Rho, GDP dissociation inhibitor (GDI) beta |
| 342 | TTGCTCTATC | 5 | 0 | 5.0 | 0.01040 | Hrh4 | Histamine receptor H4 |
| 343 | TTGGATCGCG | 5 | 0 | 5.0 | 0.01040 |  |  |
| 344 | TTGTACACTT | 5 | 0 | 5.0 | 0.01040 | Serpinb9 | Serine (or cysteine) peptidase inhibitor, clade B, member 9 |
| 345 | TTGTACAGGT | 5 | 0 | 5.0 | 0.01040 |  |  |
| 346 | TTTAAGATTT | 5 | 0 | 5.0 | 0.01040 | D130067C23Rik | Trans-acting transcription factor 3 |
| 347 | TTTATAAGTT | 5 | 0 | 5.0 | 0.01040 | Tomm40 | Translocase of outer mitochondrial membrane 40 homolog (yeast) |
| 348 | TTTCTGTACA | 5 | 0 | 5.0 | 0.01040 | Mrpl11 | Mitochondrial ribosomal protein L11 |
| 349 | TTTGCTCCCG | 5 | 0 | 5.0 | 0.01040 | Mtus1 | Mitochondrial tumor suppressor 1 |
| 350 | TTTGCTTGCA | 5 | 0 | 5.0 | 0.01040 | Tardbp | TAR DNA binding protein |
| 351 | TTTGTTACAA | 5 | 0 | 5.0 | 0.01040 | Zfp503 | Zinc finger protein 503 |
| 352 | TTTTAGGAAA | 5 | 0 | 5.0 | 0.01040 | Trim44 | Tripartite motif-containing 44 |
| 353 | TTTTCTAAGA | 5 | 0 | 5.0 | 0.01040 | Pde9a | Phosphodiesterase 9A |
| 354 | AAAAGCAAGA | 5 | 1 | 5.0 | 0.04119 | Gcc2 | GRIP and coiled-coil domain containing 2 |
| 355 | AAACCAATTT | 5 | 1 | 5.0 | 0.04119 | 1100001H23Rik | RIKEN cDNA 1100001H23 gene |
| 356 | AAATTTGTTC | 5 | 1 | 5.0 | 0.04119 | AW555464 | Expressed sequence AW555464 |
| 357 | AACAGATATA | 5 | 1 | 5.0 | 0.04119 | Hiatl1 | Hippocampus abundant transcript-like 1 |
| 358 | AACTCTTGTT | 5 | 1 | 5.0 | 0.04119 | Fgl2 | Fibrinogen-like protein 2 |
| 359 | AAGATGCAGC | 5 | 1 | 5.0 | 0.04119 | Has1 | Hyaluronan synthase1 |
| 360 | AAGGGAGTCC | 5 | 1 | 5.0 | 0.04119 |  |  |
| 361 | ACCAGTGGCC | 5 | 1 | 5.0 | 0.04119 | Nelf | Nasal embryonic LHRH factor |
| 362 | ACCGTAGGAG | 5 | 1 | 5.0 | 0.04119 | EG667418 | Pleckstrin homology-like domain, family B, member 2 |
| 363 | ACCTGCATCT | 5 | 1 | 5.0 | 0.04119 | Rrp1 | Ribosomal RNA processing 1 homolog (S. cerevisiae) |
| 364 | ACTTCCTATA | 5 | 1 | 5.0 | 0.04119 |  | Transcribed locus |
| 365 | ACTTCTATAT | 5 | 1 | 5.0 | 0.04119 | BC057079 | CDNA sequence BC057079 |
| 366 | AGAATGGCAG | 5 | 1 | 5.0 | 0.04119 | Nrarp | Notch-regulated ankyrin repeat protein |
| 367 | AGCTCCACGA | 5 | 1 | 5.0 | 0.04119 |  |  |
| 368 | AGCTGGGGTT | 5 | 1 | 5.0 | 0.04119 |  | Transcribed locus |
| 369 | AGGAAATACC | 5 | 1 | 5.0 | 0.04119 | Cramp1l | Crm, cramped-like (Drosophila) |
| 370 | AGGCACAGTG | 5 | 1 | 5.0 | 0.04119 | Gtf3a | General transcription factor III A |
| 371 | AGTCATCCCT | 5 | 1 | 5.0 | 0.04119 | Cd2ap | CD2-associated protein |
| 372 | ATCAGGCCTC | 5 | 1 | 5.0 | 0.04119 | S1pr2 | Sphingosine-1-phosphate receptor 2 |
| 373 | ATCTACATAG | 5 | 1 | 5.0 | 0.04119 |  |  |
| 374 | ATCTTGGGTA | 5 | 1 | 5.0 | 0.04119 | Scnn1g | Sodium channel, nonvoltage-gated 1 gamma |
| 375 | CAAATGAAGT | 5 | 1 | 5.0 | 0.04119 | Csde1 | Cold shock domain containing E1, RNA binding |
| 376 | CAAATGGTTA | 5 | 1 | 5.0 | 0.04119 | Sec23ip | Sec23 interacting protein |
| 377 | CAACTCTCAC | 5 | 1 | 5.0 | 0.04119 | F830002L21Rik | Transcribed locus, strongly similar to XP_001478046.1 PREDICTED: hypothetical protein [Mus musculus] |
| 378 | CAAGCCTGTA | 5 | 1 | 5.0 | 0.04119 | Ttc1 | Tetratricopeptide repeat domain 1 |
| 379 | CAAGGATTGT | 5 | 1 | 5.0 | 0.04119 | Phyhd1 | Phytanoyl-CoA dioxygenase domain containing 1 |
| 380 | CAATTAAGGT | 5 | 1 | 5.0 | 0.04119 |  | E1A binding protein p300 |
| 381 | CAGAAGAAAG | 5 | 1 | 5.0 | 0.04119 | Cdk2ap1 | CDK2 (cyclin-dependent kinase 2)-associated protein 1 |
| 382 | CAGACTGGGG | 5 | 1 | 5.0 | 0.04119 |  |  |
| 383 | CCCCTGTGTA | 5 | 1 | 5.0 | 0.04119 | Irak1 | Interleukin-1 receptor-associated kinase 1 |
| 384 | CCCGACGCCC | 5 | 1 | 5.0 | 0.04119 | Ythdf1 | YTH domain family 1 |
| 385 | CCCTTCCCTT | 5 | 1 | 5.0 | 0.04119 | LOC668661 | RIKEN cDNA 2410002F23 gene |
| 386 | CCGGGCGGTG | 5 | 1 | 5.0 | 0.04119 |  |  |
| 387 | CCTACCTTTG | 5 | 1 | 5.0 | 0.04119 | Usp19 | Ubiquitin specific peptidase 19 |
| 388 | CCTCTCCAGT | 5 | 1 | 5.0 | 0.04119 | Dpp8 | Transcribed locus |
| 389 | CCTGTCTGGT | 5 | 1 | 5.0 | 0.04119 | Tm9sf2 | Transmembrane 9 superfamily member 2 |
| 390 | CCTTGGCCTC | 5 | 1 | 5.0 | 0.04119 | Hnrpdl | Heterogeneous nuclear ribonucleoprotein D-like |
| 391 | CTATGGTCAC | 5 | 1 | 5.0 | 0.04119 |  |  |
| 392 | CTCACCTGAT | 5 | 1 | 5.0 | 0.04119 | 0610010O12Rik | RIKEN cDNA 0610010O12 gene |
| 393 | CTCCTGCCAA | 5 | 1 | 5.0 | 0.04119 | Rufy1 | RUN and FYVE domain containing 1 |
| 394 | CTCTGTTTTT | 5 | 1 | 5.0 | 0.04119 | Evc2 | Ellis van Creveld syndrome 2 homolog (human) |
| 395 | CTCTTCTTCA | 5 | 1 | 5.0 | 0.04119 |  | Transcribed locus |
| 396 | CTGAGCCAGA | 5 | 1 | 5.0 | 0.04119 | Hsd17b4 | Hydroxysteroid (17-beta) dehydrogenase 4 |
| 397 | CTGGAATTTG | 5 | 1 | 5.0 | 0.04119 | Coasy | Coenzyme A synthase |
| 398 | CTTTGTTCTC | 5 | 1 | 5.0 | 0.04119 | Znrd1 | Zinc ribbon domain containing, 1 |
| 399 | GAATTGCGGA | 5 | 1 | 5.0 | 0.04119 | Agpat6 | 1-acylglycerol-3-phosphate O-acyltransferase 6 (lysophosphatidic acid acyltransferase, zeta) |
| 400 | GACTCTAACC | 5 | 1 | 5.0 | 0.04119 | Clec12a | C-type lectin domain family 12, member a |
| 401 | GAGCTTGTAG | 5 | 1 | 5.0 | 0.04119 | Gcc1 | Golgi coiled coil 1 |
| 402 | GAGGACTTGA | 5 | 1 | 5.0 | 0.04119 | Taf10 | TAF10 RNA polymerase II, TATA box binding protein (TBP)-associated factor |
| 403 | GATGCTGTCA | 5 | 1 | 5.0 | 0.04119 | Abhd11 | Abhydrolase domain containing 11 |
| 404 | GCATCCAAAA | 5 | 1 | 5.0 | 0.04119 |  |  |
| 405 | GCATCTGTGT | 5 | 1 | 5.0 | 0.04119 | Ppm1m | Protein phosphatase 1M |
| 406 | GCCACCTAGC | 5 | 1 | 5.0 | 0.04119 | EG624086 | Transcribed locus, weakly similar to NP_808358.2 NACHT, leucine rich repeat and PYD containing 2 [Mus musculus] |
| 407 | GCCAGACCTG | 5 | 1 | 5.0 | 0.04119 |  | Brain protein 44 |
| 408 | GCCCTAGAGT | 5 | 1 | 5.0 | 0.04119 |  | Transcribed locus |
| 409 | GCTCCAGCTG | 5 | 1 | 5.0 | 0.04119 | 0610031O16Rik | RIKEN cDNA 0610031O16 gene |
| 410 | GCTGTTAGGG | 5 | 1 | 5.0 | 0.04119 | Pnpo | Pyridoxine 5'-phosphate oxidase |
| 411 | GCTTAAGTGT | 5 | 1 | 5.0 | 0.04119 | Reep5 | Receptor accessory protein 5 |
| 412 | GGAAGTACAG | 5 | 1 | 5.0 | 0.04119 | Mrpl51 | Mitochondrial ribosomal protein L51 |
| 413 | GGATGTACCC | 5 | 1 | 5.0 | 0.04119 | Wsb2 | WD repeat and SOCS box-containing 2 |
| 414 | GGGGGCCAGA | 5 | 1 | 5.0 | 0.04119 | Dact3 | Dapper homolog 3, antagonist of beta-catenin (xenopus) |
| 415 | GGGTAGCTGC | 5 | 1 | 5.0 | 0.04119 | Hist1h1c | Histone cluster 1, H1c |
| 416 | GGTTCCTTTT | 5 | 1 | 5.0 | 0.04119 | Arrdc4 | Arrestin domain containing 4 |
| 417 | GGTTTTAAGT | 5 | 1 | 5.0 | 0.04119 | Eif1 | Eukaryotic translation initiation factor 1 |
| 418 | GTAGCAAAAA | 5 | 1 | 5.0 | 0.04119 |  |  |
| 419 | GTAGGCACGG | 5 | 1 | 5.0 | 0.04119 |  |  |
| 420 | GTCGGCCGCC | 5 | 1 | 5.0 | 0.04119 | Trap1 | TNF receptor-associated protein 1 |
| 421 | GTCTTGGGCG | 5 | 1 | 5.0 | 0.04119 | Htra1 | HtrA serine peptidase 1 |
| 422 | GTGAGGTTTG | 5 | 1 | 5.0 | 0.04119 | 4833432E10Rik | RIKEN cDNA 4833432E10 gene |
| 423 | GTGGCTTATA | 5 | 1 | 5.0 | 0.04119 | Ddx27 | DEAD (Asp-Glu-Ala-Asp) box polypeptide 27 |
| 424 | GTTAATGCTA | 5 | 1 | 5.0 | 0.04119 | Ythdc1 | YTH domain containing 1 |
| 425 | GTTAGTTCAG | 5 | 1 | 5.0 | 0.04119 |  |  |
| 426 | TAAATCAAAG | 5 | 1 | 5.0 | 0.04119 | Gtf3c5 | General transcription factor IIIC, polypeptide 5 |
| 427 | TAAATTAAGA | 5 | 1 | 5.0 | 0.04119 | Hexb | Hexosaminidase B |
| 428 | TAAGATGGCA | 5 | 1 | 5.0 | 0.04119 | Trim24 | Tripartite motif-containing 24 |
| 429 | TACCTCAGAT | 5 | 1 | 5.0 | 0.04119 |  |  |
| 430 | TACTCCGCTA | 5 | 1 | 5.0 | 0.04119 |  |  |
| 431 | TAGTTTTGTC | 5 | 1 | 5.0 | 0.04119 | Fgfr1op2 | FGFR1 oncogene partner 2 |
| 432 | TATGTCTGTG | 5 | 1 | 5.0 | 0.04119 | Commd2 | COMM domain containing 2 |
| 433 | TCACGGAGTA | 5 | 1 | 5.0 | 0.04119 | Wwtr1 | WW domain containing transcription regulator 1 |
| 434 | TCCTAGAAAA | 5 | 1 | 5.0 | 0.04119 |  |  |
| 435 | TCCTCTCTCT | 5 | 1 | 5.0 | 0.04119 | Copa | Coatomer protein complex subunit alpha |
| 436 | TCGCATTTTA | 5 | 1 | 5.0 | 0.04119 | Armcx3 | Armadillo repeat containing, X-linked 3 |
| 437 | TCGGAGAAGA | 5 | 1 | 5.0 | 0.04119 | Ptms | Parathymosin |
| 438 | TCGGCCCCCA | 5 | 1 | 5.0 | 0.04119 |  |  |
| 439 | TCTAGATTAG | 5 | 1 | 5.0 | 0.04119 |  | Glyceronephosphate O-acyltransferase |
| 440 | TGAGAAAATG | 5 | 1 | 5.0 | 0.04119 | Col4a5 | Collagen, type IV, alpha 5 |
| 441 | TGAGGGTAGG | 5 | 1 | 5.0 | 0.04119 |  |  |
| 442 | TGCTTATACC | 5 | 1 | 5.0 | 0.04119 | Ndufb6 | NADH dehydrogenase (ubiquinone) 1 beta subcomplex, 6 |
| 443 | TGGATTCATT | 5 | 1 | 5.0 | 0.04119 | Pcdha8 | Protocadherin alpha 7 |
| 444 | TGGGTTGTGG | 5 | 1 | 5.0 | 0.04119 |  | Tubulin tyrosine ligase |
| 445 | TTAAAACTGA | 5 | 1 | 5.0 | 0.04119 |  | CDNA clone IMAGE:40049146 |
| 446 | TTAAAGATAG | 5 | 1 | 5.0 | 0.04119 |  | RIKEN cDNA 1200016E24 gene |
| 447 | TTAACAATAG | 5 | 1 | 5.0 | 0.04119 |  |  |
| 448 | TTAGGACTCT | 5 | 1 | 5.0 | 0.04119 | Egln1 | EGL nine homolog 1 (C. elegans) |
| 449 | TTATAGCTGC | 5 | 1 | 5.0 | 0.04119 |  |  |
| 450 | TTCAGGCACT | 5 | 1 | 5.0 | 0.04119 | Ttc13 | Tetratricopeptide repeat domain 13 |
| 451 | TTCAGTAATG | 5 | 1 | 5.0 | 0.04119 |  | Natural killer tumor recognition sequence |
| 452 | TTCCCCACGT | 5 | 1 | 5.0 | 0.04119 | Cdc2l1 | Cell division cycle 2-like 1 |
| 453 | TTCTGGCCTG | 5 | 1 | 5.0 | 0.04119 | 1110008P14Rik | RIKEN cDNA 1110008P14 gene |
| 454 | TTGAAGCATA | 5 | 1 | 5.0 | 0.04119 |  |  |
| 455 | TTTAGATGTT | 5 | 1 | 5.0 | 0.04119 | Rab1 | RAB1, member RAS oncogene family |
| 456 | TTTCTTCTCT | 5 | 1 | 5.0 | 0.04119 |  | PHD finger protein 23 |
| 457 | TTTGTATGGG | 5 | 1 | 5.0 | 0.04119 | Bmi1 | Bmi1 polycomb ring finger oncogene |
| 458 | TTTGTTGATT | 5 | 1 | 5.0 | 0.04119 | Tmem18 | Transmembrane protein 18 |
| 459 | CGACCAGCAG | 14 | 3 | 4.7 | 0.00042 | Vdac2 | Voltage-dependent anion channel 2 |
| 460 | GCGTAGCGCT | 14 | 3 | 4.7 | 0.00042 |  |  |
| 461 | GGCTGCATTC | 14 | 3 | 4.7 | 0.00042 | Vkorc1 | Vitamin K epoxide reductase complex, subunit 1 |
| 462 | TATGTCAGTA | 14 | 3 | 4.7 | 0.00042 | Rap1gap | Rap1 GTPase-activating protein |
| 463 | TCAGCTGAAT | 14 | 3 | 4.7 | 0.00042 | Gosr2 | Golgi SNAP receptor complex member 2 |
| 464 | TTGTTTTCCT | 14 | 3 | 4.7 | 0.00042 | Etv6 | Ets variant gene 6 (TEL oncogene) |
| 465 | TAACTGCACA | 46 | 10 | 4.6 | 0.00000 | Casc4 | Cancer susceptibility candidate 4 |
| 466 | AGCAGACAGT | 13 | 3 | 4.3 | 0.00090 | LOC677557 | Hypothetical LOC666452 |
| 467 | CACCACCACC | 13 | 3 | 4.3 | 0.00090 | Orai3 | ORAI calcium release-activated calcium modulator 3 |
| 468 | AGAGGGTTTT | 17 | 4 | 4.3 | 0.00013 |  | Reticulon 3 |
| 469 | GAAACAATGC | 17 | 4 | 4.3 | 0.00013 | Zdhhc3 | Zinc finger, DHHC domain containing 3 |
| 470 | GCCTGTCTTC | 21 | 5 | 4.2 | 0.00003 | Tmem205 | Transmembrane protein 205 |
| 471 | GTGTCTGATA | 21 | 5 | 4.2 | 0.00003 | Col4a1 | Collagen, type IV, alpha 1 |
| 472 | GATAATAAAG | 20 | 5 | 4.0 | 0.00006 | Notch4 | Notch gene homolog 4 (Drosophila) |
| 473 | AAGATCAGTT | 8 | 2 | 4.0 | 0.01264 | Arl6ip5 | ADP-ribosylation factor-like 6 interacting protein 5 |
| 474 | AAGGAAGAGG | 8 | 2 | 4.0 | 0.01264 | Vegfa | Vascular endothelial growth factor A |
| 475 | ATGCTATGTA | 8 | 2 | 4.0 | 0.01264 | BC022623 | CDNA sequence BC022623 |
| 476 | ATTCCCCTCC | 8 | 2 | 4.0 | 0.01264 | Dpy30 | Dpy-30 homolog (C. elegans) |
| 477 | ATTTCTGCAT | 8 | 2 | 4.0 | 0.01264 | Klhl9 | Kelch-like 9 (Drosophila) |
| 478 | CAAGTTTGCT | 8 | 2 | 4.0 | 0.01264 | Eef1a1 | Eukaryotic translation elongation factor 1 alpha 1 |
| 479 | CAGGACAAGA | 8 | 2 | 4.0 | 0.01264 | Pdgfrb | Platelet derived growth factor receptor, beta polypeptide |
| 480 | CAGTTACAAA | 8 | 2 | 4.0 | 0.01264 |  | Transcribed locus |
| 481 | CCATTTAGCA | 8 | 2 | 4.0 | 0.01264 |  |  |
| 482 | CCTACCTGCA | 8 | 2 | 4.0 | 0.01264 | Rpl27a | Ribosomal protein L27a |
| 483 | CTCGCTAATG | 8 | 2 | 4.0 | 0.01264 | Usp7 | Ubiquitin specific peptidase 7 |
| 484 | CTGAGCTGTG | 8 | 2 | 4.0 | 0.01264 | Rela | V-rel reticuloendotheliosis viral oncogene homolog A (avian) |
| 485 | GAAACCAGGA | 8 | 2 | 4.0 | 0.01264 | Tapt1 | Transmembrane anterior posterior transformation 1 |
| 486 | GACTTGTTTC | 8 | 2 | 4.0 | 0.01264 | Sept8 | Septin 8 |
| 487 | GAGCGAGAAC | 8 | 2 | 4.0 | 0.01264 | Rftn1 | Raftlin lipid raft linker 1 |
| 488 | GAGGCATTTC | 8 | 2 | 4.0 | 0.01264 | Spint1 | Serine protease inhibitor, Kunitz type 1 |
| 489 | GCGTTGCGTG | 8 | 2 | 4.0 | 0.01264 | Kpna6 | Karyopherin (importin) alpha 6 |
| 490 | GTGACACGGG | 8 | 2 | 4.0 | 0.01264 |  |  |
| 491 | GTGCGCTAGA | 8 | 2 | 4.0 | 0.01264 | Rnasek | Ribonuclease, RNase K |
| 492 | GTGGTTCACA | 8 | 2 | 4.0 | 0.01264 | Styx | Phosphoserine/threonine/tyrosine interaction protein |
| 493 | TAAAGCCACT | 8 | 2 | 4.0 | 0.01264 | Psmd6 | Proteasome (prosome, macropain) 26S subunit, non-ATPase, 6 |
| 494 | TAAGATTTCA | 8 | 2 | 4.0 | 0.01264 |  | Heterogeneous nuclear ribonucleoprotein R |
| 495 | TACTTGTATG | 8 | 2 | 4.0 | 0.01264 | Hoxa4 | Homeo box A4 |
| 496 | TAGATCATTT | 8 | 2 | 4.0 | 0.01264 |  |  |
| 497 | TGTAAAGGAG | 8 | 2 | 4.0 | 0.01264 | Atf4 | Activating transcription factor 4 |
| 498 | TTGGAACCCT | 8 | 2 | 4.0 | 0.01264 | Sephs1 | Selenophosphate synthetase 1 |
| 499 | AAAACGCAGT | 4 | 0 | 4.0 | 0.02557 |  |  |
| 500 | AAAAGGAACA | 4 | 0 | 4.0 | 0.02557 | Dusp18 | Dual specificity phosphatase 18 |
| 501 | AAAAGGTTGG | 4 | 0 | 4.0 | 0.02557 |  |  |
| 502 | AAAAGTAATC | 4 | 0 | 4.0 | 0.02557 | Spa17 | Sperm autoantigenic protein 17 |
| 503 | AAAATCTTAA | 4 | 0 | 4.0 | 0.02557 | Tmem166 | Transmembrane protein 166 |
| 504 | AAACAGAAGT | 4 | 0 | 4.0 | 0.02557 |  | Ubiquitin specific peptidase 15 |
| 505 | AAAGATAACT | 4 | 0 | 4.0 | 0.02557 | Ccdc126 | Coiled-coil domain containing 126 |
| 506 | AAATAGCAGA | 4 | 0 | 4.0 | 0.02557 |  |  |
| 507 | AAATGAAAGG | 4 | 0 | 4.0 | 0.02557 |  |  |
| 508 | AAATTGCTTC | 4 | 0 | 4.0 | 0.02557 | Dhrs3 | Dehydrogenase/reductase (SDR family) member 3 |
| 509 | AAATTGGCAG | 4 | 0 | 4.0 | 0.02557 | Atrnl1 | Attractin like 1 |
| 510 | AACAAGTCAT | 4 | 0 | 4.0 | 0.02557 | Sugt1 | SGT1, suppressor of G2 allele of SKP1 (S. cerevisiae) |
| 511 | AACATAAAGC | 4 | 0 | 4.0 | 0.02557 | Atf5 | Activating transcription factor 5 |
| 512 | AACCAGATGT | 4 | 0 | 4.0 | 0.02557 |  | Glypican 6 |
| 513 | AACTAATAAA | 4 | 0 | 4.0 | 0.02557 | Adam5 | A disintegrin and metallopeptidase domain 5 |
| 514 | AACTCCTAAG | 4 | 0 | 4.0 | 0.02557 |  | Transcribed locus |
| 515 | AACTGCATTG | 4 | 0 | 4.0 | 0.02557 | Bgn | Biglycan |
| 516 | AACTGGACTT | 4 | 0 | 4.0 | 0.02557 | Rhob | Ras homolog gene family, member B |
| 517 | AAGAAGATGG | 4 | 0 | 4.0 | 0.02557 | Apip | APAF1 interacting protein |
| 518 | AAGAGGAAGA | 4 | 0 | 4.0 | 0.02557 | Psma4 | Proteasome (prosome, macropain) subunit, alpha type 4 |
| 519 | AAGAGGGGGA | 4 | 0 | 4.0 | 0.02557 |  |  |
| 520 | AAGATCTGGT | 4 | 0 | 4.0 | 0.02557 | Bsdc1 | BSD domain containing 1 |
| 521 | AAGATTGGGG | 4 | 0 | 4.0 | 0.02557 | Cd44 | CD44 antigen |
| 522 | AAGCACACGG | 4 | 0 | 4.0 | 0.02557 |  |  |
| 523 | AAGCAGACTG | 4 | 0 | 4.0 | 0.02557 | Exoc3 | Exocyst complex component 3 |
| 524 | AAGCAGCAGC | 4 | 0 | 4.0 | 0.02557 | 2700038C09Rik | RIKEN cDNA 2700038C09 gene |
| 525 | AAGCCAAAGC | 4 | 0 | 4.0 | 0.02557 | Lynx1 | Ly6/neurotoxin 1 |
| 526 | AAGCCTTTGT | 4 | 0 | 4.0 | 0.02557 | Tbrg1 | Transforming growth factor beta regulated gene 1 |
| 527 | AAGCGTGCAG | 4 | 0 | 4.0 | 0.02557 | Pfn2 | Profilin 2 |
| 528 | AAGCTAAAGC | 4 | 0 | 4.0 | 0.02557 | Lipa | Lysosomal acid lipase A |
| 529 | AAGCTCCCGG | 4 | 0 | 4.0 | 0.02557 |  |  |
| 530 | AAGCTGAATT | 4 | 0 | 4.0 | 0.02557 | Tmem195 | Transmembrane protein 195 |
| 531 | AAGGCCTGGG | 4 | 0 | 4.0 | 0.02557 | Fancc | Fanconi anemia, complementation group C |
| 532 | AAGGTAGATG | 4 | 0 | 4.0 | 0.02557 | 100039707 | Similar to 5, 10-methenyltetrahydrofolate synthetase |
| 533 | AAGTAATTTA | 4 | 0 | 4.0 | 0.02557 |  |  |
| 534 | AATACTGGTT | 4 | 0 | 4.0 | 0.02557 |  |  |
| 535 | AATAGACACG | 4 | 0 | 4.0 | 0.02557 | Mllt1 | Myeloid/lymphoid or mixed-lineage leukemia (trithorax homolog, Drosophila); translocated to, 1 |
| 536 | AATCAGTGGC | 4 | 0 | 4.0 | 0.02557 |  | Cyclin I |
| 537 | AATGCTTGAT | 4 | 0 | 4.0 | 0.02557 | AI256676 | Retinoblastoma binding protein 7 |
| 538 | AATTTCTCCT | 4 | 0 | 4.0 | 0.02557 | Pisd | Phosphatidylserine decarboxylase |
| 539 | ACAAAAACCA | 4 | 0 | 4.0 | 0.02557 |  |  |
| 540 | ACACCCTGGA | 4 | 0 | 4.0 | 0.02557 |  | Morf4 family associated protein 1 |
| 541 | ACAGAGAGTG | 4 | 0 | 4.0 | 0.02557 |  |  |
| 542 | ACAGCCGTTC | 4 | 0 | 4.0 | 0.02557 | Wwp1 | WW domain containing E3 ubiquitin protein ligase 1 |
| 543 | ACCAAAAGTC | 4 | 0 | 4.0 | 0.02557 |  |  |
| 544 | ACCAAGTGTA | 4 | 0 | 4.0 | 0.02557 | Ndufs8 | NADH dehydrogenase (ubiquinone) Fe-S protein 8 |
| 545 | ACCATATCAG | 4 | 0 | 4.0 | 0.02557 | Anxa5 | Annexin A5 |
| 546 | ACCGATCATC | 4 | 0 | 4.0 | 0.02557 | Tmcc3 | Transmembrane and coiled coil domains 3 |
| 547 | ACCGGCGGCC | 4 | 0 | 4.0 | 0.02557 | Clec3b | C-type lectin domain family 3, member b |
| 548 | ACTATACCAT | 4 | 0 | 4.0 | 0.02557 | D030029J20Rik | Transcribed locus |
| 549 | ACTCAGCCAG | 4 | 0 | 4.0 | 0.02557 |  |  |
| 550 | ACTCCGGGGA | 4 | 0 | 4.0 | 0.02557 | Gria1 | Glutamate receptor, ionotropic, AMPA1 (alpha 1) |
| 551 | ACTCCTGGAC | 4 | 0 | 4.0 | 0.02557 | Tob2 | Transducer of ERBB2, 2 |
| 552 | ACTCTATCAA | 4 | 0 | 4.0 | 0.02557 | Wfdc1 | WAP four-disulfide core domain 1 |
| 553 | ACTCTGGGCC | 4 | 0 | 4.0 | 0.02557 | Ryk | Receptor-like tyrosine kinase |
| 554 | AGAATTTGGT | 4 | 0 | 4.0 | 0.02557 | Smg7 | Smg-7 homolog, nonsense mediated mRNA decay factor (C. elegans) |
| 555 | AGACAATGCT | 4 | 0 | 4.0 | 0.02557 | Efnb1 | Ephrin B1 |
| 556 | AGACACAAAT | 4 | 0 | 4.0 | 0.02557 | Exosc8 | Exosome component 8 |
| 557 | AGACACAATG | 4 | 0 | 4.0 | 0.02557 | Hsdl2 | Hydroxysteroid dehydrogenase like 2 |
| 558 | AGACACTGTC | 4 | 0 | 4.0 | 0.02557 | Tubd1 | Tubulin, delta 1 |
| 559 | AGACCCTATT | 4 | 0 | 4.0 | 0.02557 | 668253 | Deleted in lymphocytic leukemia, 2 |
| 560 | AGACCGGATC | 4 | 0 | 4.0 | 0.02557 |  |  |
| 561 | AGACTATTTA | 4 | 0 | 4.0 | 0.02557 | Ndst1 | N-deacetylase/N-sulfotransferase (heparan glucosaminyl) 1 |
| 562 | AGACTGTAAA | 4 | 0 | 4.0 | 0.02557 |  | Transcribed locus |
| 563 | AGAGAACACT | 4 | 0 | 4.0 | 0.02557 |  |  |
| 564 | AGAGCAGGGA | 4 | 0 | 4.0 | 0.02557 | Mfsd1 | Major facilitator superfamily domain containing 1 |
| 565 | AGAGGCACTT | 4 | 0 | 4.0 | 0.02557 | Acss2 | Acyl-CoA synthetase short-chain family member 2 |
| 566 | AGAGGCGTAG | 4 | 0 | 4.0 | 0.02557 |  |  |
| 567 | AGATCACGGT | 4 | 0 | 4.0 | 0.02557 | Sigirr | Single immunoglobulin and toll-interleukin 1 receptor (TIR) domain |
| 568 | AGATTCCATT | 4 | 0 | 4.0 | 0.02557 | Ppp1r12a | Protein phosphatase 1, regulatory (inhibitor) subunit 12A |
| 569 | AGCAAAGCCC | 4 | 0 | 4.0 | 0.02557 |  | Transcribed locus, strongly similar to NP_904330.1 cytochrome c oxidase subunit I [Mus musculus] |
| 570 | AGCCAGCAGT | 4 | 0 | 4.0 | 0.02557 | Cbx7 | Chromobox homolog 7 |
| 571 | AGCCATTCTA | 4 | 0 | 4.0 | 0.02557 | Mcat | Malonyl CoA:ACP acyltransferase (mitochondrial) |
| 572 | AGCCGGCCAA | 4 | 0 | 4.0 | 0.02557 |  |  |
| 573 | AGCCTGTTTA | 4 | 0 | 4.0 | 0.02557 | Hoxa5 | Homeo box A5 |
| 574 | AGCTCTGGGG | 4 | 0 | 4.0 | 0.02557 |  | Sno, strawberry notch homolog 1 (Drosophila) |
| 575 | AGCTGAGCCG | 4 | 0 | 4.0 | 0.02557 | Tbc1d22a | TBC1 domain family, member 22a |
| 576 | AGGAAAGTGG | 4 | 0 | 4.0 | 0.02557 | Exosc7 | Exosome component 7 |
| 577 | AGGAATTTTT | 4 | 0 | 4.0 | 0.02557 | Cep70 | Centrosomal protein 70 |
| 578 | AGGCAGGCCG | 4 | 0 | 4.0 | 0.02557 | Gltscr2 | Glioma tumor suppressor candidate region gene 2 |
| 579 | AGGCCCTGGG | 4 | 0 | 4.0 | 0.02557 |  | Transcribed locus |
| 580 | AGGCTGACAA | 4 | 0 | 4.0 | 0.02557 | Tiprl | TIP41, TOR signalling pathway regulator-like (S. cerevisiae) |
| 581 | AGGGGTCTGG | 4 | 0 | 4.0 | 0.02557 | Csf1r | Colony stimulating factor 1 receptor |
| 582 | AGGTGCTGAG | 4 | 0 | 4.0 | 0.02557 | Cyp2j11 | Cytochrome P450, family 2, subfamily j, polypeptide 11 |
| 583 | AGTATTGTTG | 4 | 0 | 4.0 | 0.02557 |  |  |
| 584 | AGTGGTTTGC | 4 | 0 | 4.0 | 0.02557 | 9030411K21Rik | SAFB-like, transcription modulator |
| 585 | ATAACTGATG | 4 | 0 | 4.0 | 0.02557 |  |  |
| 586 | ATAATGAATG | 4 | 0 | 4.0 | 0.02557 | Xdh | Xanthine dehydrogenase |
| 587 | ATACACTCCA | 4 | 0 | 4.0 | 0.02557 | Hnrnpu | Heterogeneous nuclear ribonucleoprotein U |
| 588 | ATACCTTTCT | 4 | 0 | 4.0 | 0.02557 |  |  |
| 589 | ATATACCTAA | 4 | 0 | 4.0 | 0.02557 |  | CDNA clone IMAGE:40049146 |
| 590 | ATCAAGGTGG | 4 | 0 | 4.0 | 0.02557 |  |  |
| 591 | ATCCAAAATA | 4 | 0 | 4.0 | 0.02557 | A930001N09Rik | RIKEN cDNA A930001N09 gene |
| 592 | ATCCGTTGCC | 4 | 0 | 4.0 | 0.02557 |  | Eukaryotic translation initiation factor 2 alpha kinase 1 |
| 593 | ATCTACAGGC | 4 | 0 | 4.0 | 0.02557 |  |  |
| 594 | ATCTTCCCTT | 4 | 0 | 4.0 | 0.02557 | Cyp2f2 | Cytochrome P450, family 2, subfamily f, polypeptide 2 |
| 595 | ATGATAATCT | 4 | 0 | 4.0 | 0.02557 | Wdr25 | WD repeat domain 25 |
| 596 | ATGCTCCTCC | 4 | 0 | 4.0 | 0.02557 | D11Wsu99e | DNA segment, Chr 11, Wayne State University 99, expressed |
| 597 | ATGGGCAGAC | 4 | 0 | 4.0 | 0.02557 | Kif1c | Kinesin family member 1C |
| 598 | ATGTAATGAA | 4 | 0 | 4.0 | 0.02557 | Far1 | Male sterility domain containing 2 |
| 599 | ATGTTTACAG | 4 | 0 | 4.0 | 0.02557 |  |  |
| 600 | ATTAACCGTG | 4 | 0 | 4.0 | 0.02557 |  |  |
| 601 | ATTATGTGCA | 4 | 0 | 4.0 | 0.02557 |  | Transcribed locus |
| 602 | ATTCTCAGTG | 4 | 0 | 4.0 | 0.02557 | 4930589O11Rik | Transcribed locus, strongly similar to XP_928447.1 PREDICTED: hypothetical protein LOC78214 [Mus musculus] |
| 603 | ATTCTTGAAT | 4 | 0 | 4.0 | 0.02557 | AW209491 | Expressed sequence AW209491 |
| 604 | ATTGCTGTCG | 4 | 0 | 4.0 | 0.02557 |  |  |
| 605 | ATTTCAAGGG | 4 | 0 | 4.0 | 0.02557 |  |  |
| 606 | ATTTGAGAAG | 4 | 0 | 4.0 | 0.02557 | Rad23b | RAD23b homolog (S. cerevisiae) |
| 607 | ATTTTCCTTC | 4 | 0 | 4.0 | 0.02557 |  |  |
| 608 | ATTTTTATTT | 4 | 0 | 4.0 | 0.02557 | Mbnl2 | Muscleblind-like 2 |
| 609 | CAAAACATAC | 4 | 0 | 4.0 | 0.02557 |  |  |
| 610 | CAAAACTATG | 4 | 0 | 4.0 | 0.02557 | Vegfc | Vascular endothelial growth factor C |
| 611 | CAAAGACAAC | 4 | 0 | 4.0 | 0.02557 | Zfp706 | Zinc finger protein 706 |
| 612 | CAAATTCTTC | 4 | 0 | 4.0 | 0.02557 |  |  |
| 613 | CAAGCTGCAA | 4 | 0 | 4.0 | 0.02557 |  | Protein phosphatase 4, regulatory subunit 2 |
| 614 | CAAGGGAGGC | 4 | 0 | 4.0 | 0.02557 |  | Transcribed locus |
| 615 | CAAGTGGTAC | 4 | 0 | 4.0 | 0.02557 | 3100002L24Rik | RIKEN cDNA 3100002L24 gene |
| 616 | CAATAAACGC | 4 | 0 | 4.0 | 0.02557 | Dpep1 | Dipeptidase 1 (renal) |
| 617 | CAATTAGTTG | 4 | 0 | 4.0 | 0.02557 | Tmed4 | Transmembrane emp24 protein transport domain containing 4 |
| 618 | CACAACGTAG | 4 | 0 | 4.0 | 0.02557 |  |  |
| 619 | CACATTCATT | 4 | 0 | 4.0 | 0.02557 | Tex10 | Testis expressed gene 10 |
| 620 | CACATTGAGA | 4 | 0 | 4.0 | 0.02557 | Cyp4b1 | Cytochrome P450, family 4, subfamily b, polypeptide 1 |
| 621 | CACCCTTTCC | 4 | 0 | 4.0 | 0.02557 | Slc15a2 | Solute carrier family 15 (H+/peptide transporter), member 2 |
| 622 | CACCGCGGGG | 4 | 0 | 4.0 | 0.02557 |  |  |
| 623 | CACTCTGCCT | 4 | 0 | 4.0 | 0.02557 |  | Ring finger protein 139 |
| 624 | CACTGCTATG | 4 | 0 | 4.0 | 0.02557 |  |  |
| 625 | CACTTCAGAA | 4 | 0 | 4.0 | 0.02557 | Mgst1 | Microsomal glutathione S-transferase 1 |
| 626 | CAGAAGTATG | 4 | 0 | 4.0 | 0.02557 | Abcb1a | ATP-binding cassette, sub-family B (MDR/TAP), member 1A |
| 627 | CAGACAAAAC | 4 | 0 | 4.0 | 0.02557 | 5730455P16Rik | RIKEN cDNA 5730455P16 gene |
| 628 | CAGATCGTGT | 4 | 0 | 4.0 | 0.02557 | Xpo6 | Exportin 6 |
| 629 | CAGCATACAG | 4 | 0 | 4.0 | 0.02557 | Eml1 | Echinoderm microtubule associated protein like 1 |
| 630 | CAGGGCGAGA | 4 | 0 | 4.0 | 0.02557 | Irf2bp1 | Interferon regulatory factor 2 binding protein 1 |
| 631 | CAGGTTAACT | 4 | 0 | 4.0 | 0.02557 | Kif5b | Kinesin family member 5B |
| 632 | CATAAACTGT | 4 | 0 | 4.0 | 0.02557 | Shprh | SNF2 histone linker PHD RING helicase |
| 633 | CATCCCTGAC | 4 | 0 | 4.0 | 0.02557 | C430049A07Rik | Fibronectin type III domain containing 3B |
| 634 | CATCTCCGCC | 4 | 0 | 4.0 | 0.02557 | AF085738 | GNAS (guanine nucleotide binding protein, alpha stimulating) complex locus |
| 635 | CATCTTCGCC | 4 | 0 | 4.0 | 0.02557 | Rps25 | Transcribed locus, strongly similar to NP_001005528.1 ribosomal protein s25 [Rattus norvegicus] |
| 636 | CATTGTGGAA | 4 | 0 | 4.0 | 0.02557 |  | EP300 interacting inhibitor of differentiation 1 |
| 637 | CCAAAAGGTG | 4 | 0 | 4.0 | 0.02557 |  |  |
| 638 | CCAAGGTGTT | 4 | 0 | 4.0 | 0.02557 | Guf1 | GUF1 GTPase homolog (S. cerevisiae) |
| 639 | CCAGAGCTGT | 4 | 0 | 4.0 | 0.02557 |  |  |
| 640 | CCATTTTCTG | 4 | 0 | 4.0 | 0.02557 | Eif3s10 | Eukaryotic translation initiation factor 3, subunit 10 (theta) |
| 641 | CCCAAGGAGA | 4 | 0 | 4.0 | 0.02557 |  | Chaperonin subunit 4 (delta) |
| 642 | CCCACAAGTA | 4 | 0 | 4.0 | 0.02557 |  |  |
| 643 | CCCAGCCGGA | 4 | 0 | 4.0 | 0.02557 |  | Protein phosphatase 1, regulatory (inhibitor) subunit 16A |
| 644 | CCCAGTGGCT | 4 | 0 | 4.0 | 0.02557 |  | RIKEN cDNA 9130005N14 gene |
| 645 | CCCATCATCC | 4 | 0 | 4.0 | 0.02557 |  | Ribosomal RNA processing 15 homolog (S. cerevisiae) |
| 646 | CCCCCCATCC | 4 | 0 | 4.0 | 0.02557 | Cdc2l5 | Cell division cycle 2-like 5 (cholinesterase-related cell division controller) |
| 647 | CCCTTCTTTC | 4 | 0 | 4.0 | 0.02557 |  |  |
| 648 | CCGACGGGCC | 4 | 0 | 4.0 | 0.02557 |  |  |
| 649 | CCGCAGACTG | 4 | 0 | 4.0 | 0.02557 | Boc | Biregional cell adhesion molecule-related/down-regulated by oncogenes (Cdon) binding protein |
| 650 | CCGCAGGGTG | 4 | 0 | 4.0 | 0.02557 |  |  |
| 651 | CCGGACGAGG | 4 | 0 | 4.0 | 0.02557 | Ppp1r14a | Protein phosphatase 1, regulatory (inhibitor) subunit 14A |
| 652 | CCGTCATCTT | 4 | 0 | 4.0 | 0.02557 | Lrrc34 | Leucine rich repeat containing 34 |
| 653 | CCGTCTCCTG | 4 | 0 | 4.0 | 0.02557 | Mtr | 5-methyltetrahydrofolate-homocysteine methyltransferase |
| 654 | CCGTGGGCAT | 4 | 0 | 4.0 | 0.02557 | Rpl21 | Ribosomal protein L21 |
| 655 | CCTAAGTTTA | 4 | 0 | 4.0 | 0.02557 |  |  |
| 656 | CCTAGTGGCT | 4 | 0 | 4.0 | 0.02557 | Casp12 | Caspase 12 |
| 657 | CCTCAAGAGC | 4 | 0 | 4.0 | 0.02557 | Mrpl30 | Mitochondrial ribosomal protein L30 |
| 658 | CCTCCTCTAC | 4 | 0 | 4.0 | 0.02557 |  |  |
| 659 | CCTCTAAAAA | 4 | 0 | 4.0 | 0.02557 | Zbtb5 | Zinc finger and BTB domain containing 5 |
| 660 | CCTGAATGAC | 4 | 0 | 4.0 | 0.02557 | Fbxw9 | F-box and WD-40 domain protein 9 |
| 661 | CCTGATTTTA | 4 | 0 | 4.0 | 0.02557 |  |  |
| 662 | CCTGGACTCT | 4 | 0 | 4.0 | 0.02557 | Tctn2 | Tectonic family member 2 |
| 663 | CCTGGGACCA | 4 | 0 | 4.0 | 0.02557 |  | Acyl-CoA synthetase medium-chain family member 2 |
| 664 | CCTGTAAAAA | 4 | 0 | 4.0 | 0.02557 |  |  |
| 665 | CCTTACCGCT | 4 | 0 | 4.0 | 0.02557 |  | RIKEN cDNA A930034L06 gene |
| 666 | CCTTGAGTCC | 4 | 0 | 4.0 | 0.02557 | Gdnf | Glial cell line derived neurotrophic factor |
| 667 | CGAGTCTCTG | 4 | 0 | 4.0 | 0.02557 | Podxl | Podocalyxin-like |
| 668 | CGATATCTGT | 4 | 0 | 4.0 | 0.02557 | Csnk1g3 | Casein kinase 1, gamma 3 |
| 669 | CGCACTCTTG | 4 | 0 | 4.0 | 0.02557 |  |  |
| 670 | CGCGTCACTA | 4 | 0 | 4.0 | 0.02557 |  |  |
| 671 | CGGATCAAAC | 4 | 0 | 4.0 | 0.02557 |  | Vascular endothelial growth factor A |
| 672 | CGTGGATCCC | 4 | 0 | 4.0 | 0.02557 | Cdkn1c | Cyclin-dependent kinase inhibitor 1C (P57) |
| 673 | CGTTCACCAT | 4 | 0 | 4.0 | 0.02557 |  | Transcribed locus |
| 674 | CGTTGGGCTC | 4 | 0 | 4.0 | 0.02557 | Hars | Histidyl-tRNA synthetase |
| 675 | CTAGTGACTG | 4 | 0 | 4.0 | 0.02557 | AU022870 | Expressed sequence AU022870 |
| 676 | CTATCACCAG | 4 | 0 | 4.0 | 0.02557 |  |  |
| 677 | CTATTGTTTT | 4 | 0 | 4.0 | 0.02557 | Mpdz | Multiple PDZ domain protein |
| 678 | CTATTTAAAG | 4 | 0 | 4.0 | 0.02557 | Fbxl5 | F-box and leucine-rich repeat protein 5 |
| 679 | CTCACAGGCA | 4 | 0 | 4.0 | 0.02557 | Zfp523 | Zinc finger protein 523 |
| 680 | CTCAGGTCTC | 4 | 0 | 4.0 | 0.02557 | Mb | Myoglobin |
| 681 | CTCATTTCCT | 4 | 0 | 4.0 | 0.02557 |  | Serpine1 mRNA binding protein 1 |
| 682 | CTCCCCATTA | 4 | 0 | 4.0 | 0.02557 | Tspan18 | Tetraspanin 18 |
| 683 | CTCGCTAACC | 4 | 0 | 4.0 | 0.02557 | Lxn | Latexin |
| 684 | CTCTAGGTTG | 4 | 0 | 4.0 | 0.02557 |  |  |
| 685 | CTCTCAGCTG | 4 | 0 | 4.0 | 0.02557 | Mpi | Mannose phosphate isomerase |
| 686 | CTCTCTCTAG | 4 | 0 | 4.0 | 0.02557 |  |  |
| 687 | CTCTCTGTGG | 4 | 0 | 4.0 | 0.02557 | Mtmr3 | Myotubularin related protein 3 |
| 688 | CTCTTAAGGG | 4 | 0 | 4.0 | 0.02557 | Anxa5 | Annexin A5 |
| 689 | CTCTTATGTT | 4 | 0 | 4.0 | 0.02557 | Gucy1a3 | Guanylate cyclase 1, soluble, alpha 3 |
| 690 | CTGAAGCGGC | 4 | 0 | 4.0 | 0.02557 | Ace | Angiotensin I converting enzyme (peptidyl-dipeptidase A) 1 |
| 691 | CTGAAGTGAT | 4 | 0 | 4.0 | 0.02557 | Atg3 | Autophagy-related 3 (yeast) |
| 692 | CTGATCTTTA | 4 | 0 | 4.0 | 0.02557 |  |  |
| 693 | CTGCTAATGC | 4 | 0 | 4.0 | 0.02557 | Igfbp6 | Insulin-like growth factor binding protein 6 |
| 694 | CTGGCGTGAG | 4 | 0 | 4.0 | 0.02557 | Pycrl | Pyrroline-5-carboxylate reductase-like |
| 695 | CTGGGAAATG | 4 | 0 | 4.0 | 0.02557 | Gpx4 | Glutathione peroxidase 4 |
| 696 | CTGGGTGCCT | 4 | 0 | 4.0 | 0.02557 | Zfp335 | Zinc finger protein 335 |
| 697 | CTGGTACTTC | 4 | 0 | 4.0 | 0.02557 | Ninj1 | Ninjurin 1 |
| 698 | CTGGTGTTGT | 4 | 0 | 4.0 | 0.02557 | A1cf | APOBEC1 complementation factor |
| 699 | CTGTAATAAA | 4 | 0 | 4.0 | 0.02557 | Jarid1c | Jumonji, AT rich interactive domain 1C (Rbp2 like) |
| 700 | CTGTCAGTGA | 4 | 0 | 4.0 | 0.02557 | Siah2 | Seven in absentia 2 |
| 701 | CTGTGCTCCT | 4 | 0 | 4.0 | 0.02557 | Stard3nl | STARD3 N-terminal like |
| 702 | CTGTGTGGTC | 4 | 0 | 4.0 | 0.02557 | C130074G19Rik | RIKEN cDNA C130074G19 gene |
| 703 | CTTAATAGCT | 4 | 0 | 4.0 | 0.02557 | Slc11a2 | Solute carrier family 11 (proton-coupled divalent metal ion transporters), member 2 |
| 704 | CTTACATTTC | 4 | 0 | 4.0 | 0.02557 | Lmnb1 | Lamin B1 |
| 705 | CTTATTAAAC | 4 | 0 | 4.0 | 0.02557 |  | Transcribed locus |
| 706 | CTTATTGAAT | 4 | 0 | 4.0 | 0.02557 | Aldh3a2 | Aldehyde dehydrogenase family 3, subfamily A2 |
| 707 | CTTCTGGTTA | 4 | 0 | 4.0 | 0.02557 | Sema3e | Sema domain, immunoglobulin domain (Ig), short basic domain, secreted, (semaphorin) 3E |
| 708 | CTTGAATATT | 4 | 0 | 4.0 | 0.02557 | Cmpk1 | Cytidine monophosphate (UMP-CMP) kinase 1 |
| 709 | CTTGATGCTT | 4 | 0 | 4.0 | 0.02557 | Shroom2 | Shroom family member 2 |
| 710 | CTTGCTCAAT | 4 | 0 | 4.0 | 0.02557 |  |  |
| 711 | CTTTACAAAG | 4 | 0 | 4.0 | 0.02557 | Kank4 | KN motif and ankyrin repeat domains 4 |
| 712 | CTTTCTGCTC | 4 | 0 | 4.0 | 0.02557 | Igf1r | Insulin-like growth factor I receptor |
| 713 | CTTTTTCATC | 4 | 0 | 4.0 | 0.02557 |  |  |
| 714 | GAAAAAATTT | 4 | 0 | 4.0 | 0.02557 | Car8 | Carbonic anhydrase 8 |
| 715 | GAAAGAAATA | 4 | 0 | 4.0 | 0.02557 | Pwwp2b | PWWP domain containing 2B |
| 716 | GAAATGTCTG | 4 | 0 | 4.0 | 0.02557 | Kctd6 | Potassium channel tetramerisation domain containing 6 |
| 717 | GAACAAATCA | 4 | 0 | 4.0 | 0.02557 |  | Transcribed locus |
| 718 | GAAGACCTTT | 4 | 0 | 4.0 | 0.02557 |  | Transcribed locus |
| 719 | GAAGCTGAAC | 4 | 0 | 4.0 | 0.02557 |  |  |
| 720 | GAATGTCAAG | 4 | 0 | 4.0 | 0.02557 |  | Ribosomal protein S6 kinase, polypeptide 5 |
| 721 | GACAATCAGA | 4 | 0 | 4.0 | 0.02557 | 0610012G03Rik | RIKEN cDNA 0610012G03 gene |
| 722 | GACACTAACA | 4 | 0 | 4.0 | 0.02557 |  |  |
| 723 | GACCTGAAGC | 4 | 0 | 4.0 | 0.02557 | Tor1a | Torsin family 1, member A (torsin A) |
| 724 | GACTAAGGCT | 4 | 0 | 4.0 | 0.02557 |  | RIKEN cDNA 1700123O12 gene |
| 725 | GACTGAAGGT | 4 | 0 | 4.0 | 0.02557 | D330022A01Rik | Ubiquitin-activating enzyme E1-like |
| 726 | GACTTCAGTT | 4 | 0 | 4.0 | 0.02557 | Srgap2 | SLIT-ROBO Rho GTPase activating protein 2 |
| 727 | GACTTTGAAG | 4 | 0 | 4.0 | 0.02557 | Hp | Haptoglobin |
| 728 | GAGAAATAAA | 4 | 0 | 4.0 | 0.02557 |  | Transcribed locus |
| 729 | GAGACCAAAA | 4 | 0 | 4.0 | 0.02557 |  |  |
| 730 | GAGATCCTGG | 4 | 0 | 4.0 | 0.02557 | Pdcd5 | Programmed cell death 5 |
| 731 | GAGCCCTTCC | 4 | 0 | 4.0 | 0.02557 | Pdk3 | Pyruvate dehydrogenase kinase, isoenzyme 3 |
| 732 | GAGCGCCATT | 4 | 0 | 4.0 | 0.02557 |  | Transcribed locus |
| 733 | GAGTCCTGGC | 4 | 0 | 4.0 | 0.02557 | Raf1 | V-raf-leukemia viral oncogene 1 |
| 734 | GAGTTAATTA | 4 | 0 | 4.0 | 0.02557 |  |  |
| 735 | GAGTTCTCTT | 4 | 0 | 4.0 | 0.02557 | Lmna | Lamin A |
| 736 | GATAAAACTC | 4 | 0 | 4.0 | 0.02557 | Utp11l | UTP11-like, U3 small nucleolar ribonucleoprotein, (yeast) |
| 737 | GATAATGTGC | 4 | 0 | 4.0 | 0.02557 |  | ATP-binding cassette, sub-family G (WHITE), member 1 |
| 738 | GATGGCATCG | 4 | 0 | 4.0 | 0.02557 | Hdgf | Hepatoma-derived growth factor |
| 739 | GATTCCTCAC | 4 | 0 | 4.0 | 0.02557 |  |  |
| 740 | GATTCGGGCA | 4 | 0 | 4.0 | 0.02557 |  |  |
| 741 | GATTTGTCTG | 4 | 0 | 4.0 | 0.02557 | Ypel2 | Yippee-like 2 (Drosophila) |
| 742 | GCAACTTCGG | 4 | 0 | 4.0 | 0.02557 |  |  |
| 743 | GCACTGCCCT | 4 | 0 | 4.0 | 0.02557 | C230081A13Rik | RIKEN cDNA C230081A13 gene |
| 744 | GCAGGCTGGG | 4 | 0 | 4.0 | 0.02557 | RP23-157O10.6 | RIKEN cDNA 4933428G20 gene |
| 745 | GCAGTTTGTT | 4 | 0 | 4.0 | 0.02557 | Axin2 | Axin2 |
| 746 | GCATCTGTAA | 4 | 0 | 4.0 | 0.02557 |  |  |
| 747 | GCATTGTAAT | 4 | 0 | 4.0 | 0.02557 | Ces1 | Carboxylesterase 1 |
| 748 | GCATTTTCTT | 4 | 0 | 4.0 | 0.02557 |  | DNA methyltransferase 3A |
| 749 | GCCAATGCAG | 4 | 0 | 4.0 | 0.02557 |  | Transcribed locus |
| 750 | GCCACCAACG | 4 | 0 | 4.0 | 0.02557 | Ostf1 | Osteoclast stimulating factor 1 |
| 751 | GCCACTCTCT | 4 | 0 | 4.0 | 0.02557 | Ddx39 | DEAD (Asp-Glu-Ala-Asp) box polypeptide 39 |
| 752 | GCCACTTTGA | 4 | 0 | 4.0 | 0.02557 | Hmox1 | Heme oxygenase (decycling) 1 |
| 753 | GCCCAGGGGC | 4 | 0 | 4.0 | 0.02557 | Shank3 | SH3/ankyrin domain gene 3 |
| 754 | GCCCCTGCCC | 4 | 0 | 4.0 | 0.02557 |  |  |
| 755 | GCCCGAGGAC | 4 | 0 | 4.0 | 0.02557 |  | Transcribed locus, strongly similar to NP_904337.1 NADH dehydrogenase subunit 4 [Mus musculus] |
| 756 | GCCCTGGGGC | 4 | 0 | 4.0 | 0.02557 | Ufc1 | Ubiquitin-fold modifier conjugating enzyme 1 |
| 757 | GCCCTTAAAA | 4 | 0 | 4.0 | 0.02557 | Zfp187 | Zinc finger protein 187 |
| 758 | GCCTTGACCT | 4 | 0 | 4.0 | 0.02557 | Dennd4b | DENN/MADD domain containing 4B |
| 759 | GCCTTGAGAT | 4 | 0 | 4.0 | 0.02557 | Parp14 | Poly (ADP-ribose) polymerase family, member 14 |
| 760 | GCCTTTTACT | 4 | 0 | 4.0 | 0.02557 | Rer1 | RER1 retention in endoplasmic reticulum 1 homolog (S. cerevisiae) |
| 761 | GCGGCACAGT | 4 | 0 | 4.0 | 0.02557 | Lysmd1 | LysM, putative peptidoglycan-binding, domain containing 1 |
| 762 | GCGTACTCTG | 4 | 0 | 4.0 | 0.02557 | Cntd1 | Cyclin N-terminal domain containing 1 |
| 763 | GCTAAATATT | 4 | 0 | 4.0 | 0.02557 | 9930039A11Rik | RIKEN cDNA 9930039A11 gene |
| 764 | GCTATGCTAT | 4 | 0 | 4.0 | 0.02557 | BC050078 | CDNA sequence BC050078 |
| 765 | GCTCCTGTAT | 4 | 0 | 4.0 | 0.02557 | Snx13 | Sorting nexin 13 |
| 766 | GCTGAATATT | 4 | 0 | 4.0 | 0.02557 |  |  |
| 767 | GCTGCCCCCT | 4 | 0 | 4.0 | 0.02557 | Casp14 | Caspase 14 |
| 768 | GCTGCTGTGG | 4 | 0 | 4.0 | 0.02557 | Cyb561d1 | Cytochrome b-561 domain containing 1 |
| 769 | GCTGTGACCA | 4 | 0 | 4.0 | 0.02557 |  |  |
| 770 | GCTGTGGGGA | 4 | 0 | 4.0 | 0.02557 | Gjb4 | Gap junction protein, beta 4 |
| 771 | GCTTCAAGAT | 4 | 0 | 4.0 | 0.02557 | Gtlf3b | Gene trap locus F3b |
| 772 | GCTTCATAGA | 4 | 0 | 4.0 | 0.02557 | Cish | Cytokine inducible SH2-containing protein |
| 773 | GGAAATGTGT | 4 | 0 | 4.0 | 0.02557 |  |  |
| 774 | GGAAGGCGGC | 4 | 0 | 4.0 | 0.02557 |  |  |
| 775 | GGACAGAAGC | 4 | 0 | 4.0 | 0.02557 | Cnot1 | CCR4-NOT transcription complex, subunit 1 |
| 776 | GGACCATAGC | 4 | 0 | 4.0 | 0.02557 |  |  |
| 777 | GGACTTGGCG | 4 | 0 | 4.0 | 0.02557 | Actr3 | ARP3 actin-related protein 3 homolog (yeast) |
| 778 | GGAGGGAAAG | 4 | 0 | 4.0 | 0.02557 |  |  |
| 779 | GGAGTGACTA | 4 | 0 | 4.0 | 0.02557 | Aph1a | Anterior pharynx defective 1a homolog (C. elegans) |
| 780 | GGATCCACCC | 4 | 0 | 4.0 | 0.02557 | Htr5b | 5-hydroxytryptamine (serotonin) receptor 5B |
| 781 | GGCAAGAAAG | 4 | 0 | 4.0 | 0.02557 | Cbr2 | Carbonyl reductase 2 |
| 782 | GGCATCTCTT | 4 | 0 | 4.0 | 0.02557 | Galnt4 | UDP-N-acetyl-alpha-D-galactosamine:polypeptide N-acetylgalactosaminyltransferase 4 |
| 783 | GGCCCCCACA | 4 | 0 | 4.0 | 0.02557 | Chmp1a | Chromatin modifying protein 1A |
| 784 | GGCTGGTGGG | 4 | 0 | 4.0 | 0.02557 | 1500005A01Rik | RIKEN cDNA 1500005A01 gene |
| 785 | GGCTGTGTTT | 4 | 0 | 4.0 | 0.02557 | Exoc2 | Exocyst complex component 2 |
| 786 | GGGCAGCTGG | 4 | 0 | 4.0 | 0.02557 | Emilin1 | Elastin microfibril interfacer 1 |
| 787 | GGGTGAGAGC | 4 | 0 | 4.0 | 0.02557 |  |  |
| 788 | GGTCAGTCGA | 4 | 0 | 4.0 | 0.02557 |  |  |
| 789 | GTAAATTGAC | 4 | 0 | 4.0 | 0.02557 | Ndc80 | NDC80 homolog, kinetochore complex component (S. cerevisiae) |
| 790 | GTAACAAGTA | 4 | 0 | 4.0 | 0.02557 | Nat12 | N-acetyltransferase 12 |
| 791 | GTACTCTTTA | 4 | 0 | 4.0 | 0.02557 |  | LMBR1 domain containing 1 |
| 792 | GTCAGCTTTA | 4 | 0 | 4.0 | 0.02557 |  | CDNA clone IMAGE:5028619 |
| 793 | GTCATTTCCT | 4 | 0 | 4.0 | 0.02557 | 6330406I15Rik | RIKEN cDNA 6330406I15 gene |
| 794 | GTCCTTGTAC | 4 | 0 | 4.0 | 0.02557 |  |  |
| 795 | GTCTACAGAG | 4 | 0 | 4.0 | 0.02557 | AY074887 | CDNA sequence AY074887 |
| 796 | GTCTCACACC | 4 | 0 | 4.0 | 0.02557 | Pcgf3 | Polycomb group ring finger 3 |
| 797 | GTCTGGTTTG | 4 | 0 | 4.0 | 0.02557 | Cap2 | CAP, adenylate cyclase-associated protein, 2 (yeast) |
| 798 | GTGAGAAAAT | 4 | 0 | 4.0 | 0.02557 | 1110003O08Rik | F-box protein 31 |
| 799 | GTGAGCAGCG | 4 | 0 | 4.0 | 0.02557 | Bcl10 | B-cell leukemia/lymphoma 10 |
| 800 | GTGAGCCCAT | 4 | 0 | 4.0 | 0.02557 | Hsp90ab1 | Heat shock protein 90kDa alpha (cytosolic), class B member 1 |
| 801 | GTGATAGCGT | 4 | 0 | 4.0 | 0.02557 | Sorl1 | Sortilin-related receptor, LDLR class A repeats-containing |
| 802 | GTGATGCAGA | 4 | 0 | 4.0 | 0.02557 |  |  |
| 803 | GTGATTTTAT | 4 | 0 | 4.0 | 0.02557 | Stx8 | Syntaxin 8 |
| 804 | GTGCAATTTG | 4 | 0 | 4.0 | 0.02557 | Sdc2 | Syndecan 2 |
| 805 | GTGCACCTGA | 4 | 0 | 4.0 | 0.02557 | Hbb-b1 | Hemoglobin, beta adult minor chain |
| 806 | GTGCCTGCCT | 4 | 0 | 4.0 | 0.02557 | Slc12a9 | Solute carrier family 12 (potassium/chloride transporters), member 9 |
| 807 | GTGGAAAGAC | 4 | 0 | 4.0 | 0.02557 | 4930534B04Rik | RIKEN cDNA 4930534B04 gene |
| 808 | GTGGCCTTGG | 4 | 0 | 4.0 | 0.02557 |  | Transcribed locus |
| 809 | GTGGCGCACA | 4 | 0 | 4.0 | 0.02557 |  | Sidekick homolog 2 (chicken) |
| 810 | GTGGCTCGCC | 4 | 0 | 4.0 | 0.02557 | Pqlc1 | PQ loop repeat containing 1 |
| 811 | GTGTAAGAAG | 4 | 0 | 4.0 | 0.02557 |  | Transcribed locus |
| 812 | GTGTCCAGCT | 4 | 0 | 4.0 | 0.02557 | Cables2 | Cdk5 and Abl enzyme substrate 2 |
| 813 | GTTAACTGAA | 4 | 0 | 4.0 | 0.02557 | Smc3 | Structural maintenace of chromosomes 3 |
| 814 | GTTCAAATGT | 4 | 0 | 4.0 | 0.02557 |  |  |
| 815 | GTTCTTCCTG | 4 | 0 | 4.0 | 0.02557 |  | Hematological and neurological expressed 1-like |
| 816 | GTTTAAAATA | 4 | 0 | 4.0 | 0.02557 |  | S-adenosylhomocysteine hydrolase-like 2 |
| 817 | GTTTATTAAA | 4 | 0 | 4.0 | 0.02557 | Ttc35 | Tetratricopeptide repeat domain 35 |
| 818 | GTTTGTTACC | 4 | 0 | 4.0 | 0.02557 |  |  |
| 819 | GTTTTAGTTT | 4 | 0 | 4.0 | 0.02557 |  | Transcribed locus, weakly similar to XP_001478155.1 PREDICTED: hypothetical protein [Mus musculus] |
| 820 | GTTTTGTTTC | 4 | 0 | 4.0 | 0.02557 | Ap4b1 | Adaptor-related protein complex AP-4, beta 1 |
| 821 | GTTTTTGTTT | 4 | 0 | 4.0 | 0.02557 | Dnd1 | Dead end homolog 1 (zebrafish) |
| 822 | TAAAATTAGT | 4 | 0 | 4.0 | 0.02557 |  |  |
| 823 | TAAACCTTGC | 4 | 0 | 4.0 | 0.02557 | Gna14 | Guanine nucleotide binding protein, alpha 14 |
| 824 | TAAACTCAAG | 4 | 0 | 4.0 | 0.02557 | Rb1cc1 | RB1-inducible coiled-coil 1 |
| 825 | TAAATAAATA | 4 | 0 | 4.0 | 0.02557 |  | Transcribed locus |
| 826 | TAAATATAGT | 4 | 0 | 4.0 | 0.02557 |  | Ectonucleotide pyrophosphatase/phosphodiesterase 4 |
| 827 | TAACACTCAG | 4 | 0 | 4.0 | 0.02557 |  | Mitogen-activated protein kinase-activated protein kinase 3 |
| 828 | TAACCATCTG | 4 | 0 | 4.0 | 0.02557 | Ppap2b | Phosphatidic acid phosphatase type 2B |
| 829 | TAAGAACATA | 4 | 0 | 4.0 | 0.02557 | Col15a1 | Collagen, type XV, alpha 1 |
| 830 | TAAGTGGTGC | 4 | 0 | 4.0 | 0.02557 |  |  |
| 831 | TAATAGTGTT | 4 | 0 | 4.0 | 0.02557 | Rsbn1 | Rosbin, round spermatid basic protein 1 |
| 832 | TACAAATAAA | 4 | 0 | 4.0 | 0.02557 |  | Transcribed locus |
| 833 | TACAGAATTA | 4 | 0 | 4.0 | 0.02557 | Adam17 | A disintegrin and metallopeptidase domain 17 |
| 834 | TACAGTATAA | 4 | 0 | 4.0 | 0.02557 | Inhbb | Inhibin beta-B |
| 835 | TACCATTTAA | 4 | 0 | 4.0 | 0.02557 | Tmem184c | Transmembrane protein 34 |
| 836 | TACTTCAGGC | 4 | 0 | 4.0 | 0.02557 |  | Uroplakin 3B |
| 837 | TACTTCAGTT | 4 | 0 | 4.0 | 0.02557 |  |  |
| 838 | TACTTGGTGT | 4 | 0 | 4.0 | 0.02557 | Itfg1 | Integrin alpha FG-GAP repeat containing 1 |
| 839 | TAGAAACTAA | 4 | 0 | 4.0 | 0.02557 | Spata6 | Spermatogenesis associated 6 |
| 840 | TAGAACTAAT | 4 | 0 | 4.0 | 0.02557 | Esf1 | ESF1, nucleolar pre-rRNA processing protein, homolog (S. cerevisiae) |
| 841 | TAGAAGAATG | 4 | 0 | 4.0 | 0.02557 | Cntn5 | Contactin 5 |
| 842 | TAGACTGCTT | 4 | 0 | 4.0 | 0.02557 | Elmo2 | Engulfment and cell motility 2, ced-12 homolog (C. elegans) |
| 843 | TAGATGAGTT | 4 | 0 | 4.0 | 0.02557 |  | PRP38 pre-mRNA processing factor 38 (yeast) domain containing A |
| 844 | TAGCTGAGTT | 4 | 0 | 4.0 | 0.02557 | Smndc1 | Survival motor neuron domain containing 1 |
| 845 | TATAATCCAC | 4 | 0 | 4.0 | 0.02557 |  |  |
| 846 | TATAGCTGTA | 4 | 0 | 4.0 | 0.02557 | Tspyl4 | TSPY-like 4 |
| 847 | TATGATCCCG | 4 | 0 | 4.0 | 0.02557 | Smad2 | MAD homolog 2 (Drosophila) |
| 848 | TATTTTGTGG | 4 | 0 | 4.0 | 0.02557 | Slc6a6 | Solute carrier family 6 (neurotransmitter transporter, taurine), member 6 |
| 849 | TCAACCAGAA | 4 | 0 | 4.0 | 0.02557 | Mospd1 | Motile sperm domain containing 1 |
| 850 | TCACAGACGC | 4 | 0 | 4.0 | 0.02557 |  |  |
| 851 | TCACCTTTCC | 4 | 0 | 4.0 | 0.02557 |  |  |
| 852 | TCAGAACAAG | 4 | 0 | 4.0 | 0.02557 | Zmat5 | Zinc finger, matrin type 5 |
| 853 | TCAGCCGCTA | 4 | 0 | 4.0 | 0.02557 | 1110007C09Rik | RIKEN cDNA 1110007C09 gene |
| 854 | TCAGTACAGA | 4 | 0 | 4.0 | 0.02557 |  | Transcribed locus |
| 855 | TCAGTGACCT | 4 | 0 | 4.0 | 0.02557 | Col14a1 | Collagen, type XIV, alpha 1 |
| 856 | TCATCTCTTT | 4 | 0 | 4.0 | 0.02557 | Shpk | Sedoheptulokinase |
| 857 | TCCAAAAGCT | 4 | 0 | 4.0 | 0.02557 |  | RIKEN cDNA 3010026O09 gene |
| 858 | TCCACTCAGA | 4 | 0 | 4.0 | 0.02557 | Bgn | Biglycan |
| 859 | TCCAGATTTG | 4 | 0 | 4.0 | 0.02557 | Lipm | Lipase, family member M |
| 860 | TCCCTCCTTA | 4 | 0 | 4.0 | 0.02557 | Atp6v0d1 | ATPase, H+ transporting, lysosomal V0 subunit D1 |
| 861 | TCCGAGGGGG | 4 | 0 | 4.0 | 0.02557 | Trip10 | Thyroid hormone receptor interactor 10 |
| 862 | TCCTGGCTCC | 4 | 0 | 4.0 | 0.02557 | Baiap2l1 | BAI1-associated protein 2-like 1 |
| 863 | TCCTTGCTAT | 4 | 0 | 4.0 | 0.02557 |  |  |
| 864 | TCGCCTGAAC | 4 | 0 | 4.0 | 0.02557 |  |  |
| 865 | TCGGAGAAGT | 4 | 0 | 4.0 | 0.02557 |  |  |
| 866 | TCTAATTCTC | 4 | 0 | 4.0 | 0.02557 | Frem1 | Fras1 related extracellular matrix protein 1 |
| 867 | TCTATCTCAG | 4 | 0 | 4.0 | 0.02557 | 2510006D16Rik | RIKEN cDNA 2510006D16 gene |
| 868 | TCTGCTAAAG | 4 | 0 | 4.0 | 0.02557 | Hmgb1-rs17 | High mobility group box 1, related sequence 17 |
| 869 | TCTGGATTTT | 4 | 0 | 4.0 | 0.02557 | Mansc1 | MANSC domain containing 1 |
| 870 | TCTGTATAAT | 4 | 0 | 4.0 | 0.02557 | Wbscr18 | Williams-Beuren syndrome chromosome region 18 homolog (human) |
| 871 | TCTTCTCTTA | 4 | 0 | 4.0 | 0.02557 | Bap1 | Brca1 associated protein 1 |
| 872 | TCTTTCTGCT | 4 | 0 | 4.0 | 0.02557 | Plod2 | Procollagen lysine, 2-oxoglutarate 5-dioxygenase 2 |
| 873 | TGAAACCGCG | 4 | 0 | 4.0 | 0.02557 |  | Transcribed locus |
| 874 | TGACCCGGCG | 4 | 0 | 4.0 | 0.02557 | Ppp2cb | Protein phosphatase 2 (formerly 2A), catalytic subunit, beta isoform |
| 875 | TGACCTCACA | 4 | 0 | 4.0 | 0.02557 | Ccbl1 | Cysteine conjugate-beta lyase 1 |
| 876 | TGAGAGGGCA | 4 | 0 | 4.0 | 0.02557 |  |  |
| 877 | TGAGATATGT | 4 | 0 | 4.0 | 0.02557 |  | Ankyrin repeat domain 17 |
| 878 | TGAGCCAGGC | 4 | 0 | 4.0 | 0.02557 |  | Polymerase (RNA) III (DNA directed) polypeptide A |
| 879 | TGATCACGGC | 4 | 0 | 4.0 | 0.02557 | Rab31 | RAB31, member RAS oncogene family |
| 880 | TGATTTTCAT | 4 | 0 | 4.0 | 0.02557 | 1200013P24Rik | RIKEN cDNA 1200013P24 gene |
| 881 | TGCAAGCTGT | 4 | 0 | 4.0 | 0.02557 | Usp16 | Ubiquitin specific peptidase 16 |
| 882 | TGCACAAATA | 4 | 0 | 4.0 | 0.02557 | Mmp7 | Matrix metallopeptidase 7 |
| 883 | TGCACTGCTG | 4 | 0 | 4.0 | 0.02557 | Med17 | Mediator complex subunit 17 |
| 884 | TGCATCTCTG | 4 | 0 | 4.0 | 0.02557 | Pik3r4 | Phosphatidylinositol 3 kinase, regulatory subunit, polypeptide 4, p150 |
| 885 | TGCCAAAGCT | 4 | 0 | 4.0 | 0.02557 |  |  |
| 886 | TGCCATTGTA | 4 | 0 | 4.0 | 0.02557 | Crk | V-crk sarcoma virus CT10 oncogene homolog (avian) |
| 887 | TGCCCGGTGT | 4 | 0 | 4.0 | 0.02557 |  | WW, C2 and coiled-coil domain containing 2 |
| 888 | TGCCTTTTTC | 4 | 0 | 4.0 | 0.02557 |  | Transcribed locus |
| 889 | TGCTCACAAC | 4 | 0 | 4.0 | 0.02557 | Bmp1 | Bone morphogenetic protein 1 |
| 890 | TGCTGCAGTG | 4 | 0 | 4.0 | 0.02557 | Heatr3 | HEAT repeat containing 3 |
| 891 | TGCTGTGTTG | 4 | 0 | 4.0 | 0.02557 | Gcsh | Glycine cleavage system protein H (aminomethyl carrier) |
| 892 | TGGATCGTGA | 4 | 0 | 4.0 | 0.02557 |  |  |
| 893 | TGGCAAATGC | 4 | 0 | 4.0 | 0.02557 |  | Heparan sulfate 2-O-sulfotransferase 1 |
| 894 | TGGCTGCTGG | 4 | 0 | 4.0 | 0.02557 | Taok3 | TAO kinase 3 |
| 895 | TGGCTGGCAC | 4 | 0 | 4.0 | 0.02557 |  |  |
| 896 | TGGCTGTGTA | 4 | 0 | 4.0 | 0.02557 | Klk1b9 | Kallikrein 1-related peptidase b9 |
| 897 | TGGGCCCTTG | 4 | 0 | 4.0 | 0.02557 |  |  |
| 898 | TGGGCTCCAG | 4 | 0 | 4.0 | 0.02557 | Eif2b3 | Eukaryotic translation initiation factor 2B, subunit 3 |
| 899 | TGGGGACCTG | 4 | 0 | 4.0 | 0.02557 |  | Myosin phosphatase Rho interacting protein |
| 900 | TGGGTGCTGG | 4 | 0 | 4.0 | 0.02557 | LOC100043538 | CDNA sequence BC027582 |
| 901 | TGGTTTTTGT | 4 | 0 | 4.0 | 0.02557 | 1700023I07Rik | RIKEN cDNA 1700023I07 gene |
| 902 | TGTAAACAAG | 4 | 0 | 4.0 | 0.02557 | D930001I22Rik | RIKEN cDNA D930001I22 gene |
| 903 | TGTAACCCAT | 4 | 0 | 4.0 | 0.02557 | Dapk1 | Death associated protein kinase 1 |
| 904 | TGTATGTGTC | 4 | 0 | 4.0 | 0.02557 |  | Transcribed locus |
| 905 | TGTCATTTAA | 4 | 0 | 4.0 | 0.02557 | AU016128 | Choline phosphotransferase 1 |
| 906 | TGTCTGAGGC | 4 | 0 | 4.0 | 0.02557 |  | Stromal cell derived factor 4 |
| 907 | TGTGCTCTAA | 4 | 0 | 4.0 | 0.02557 | Yeats4 | YEATS domain containing 4 |
| 908 | TGTTACCAAC | 4 | 0 | 4.0 | 0.02557 | Vps13a | Vacuolar protein sorting 13A (yeast) |
| 909 | TGTTCCCTCT | 4 | 0 | 4.0 | 0.02557 | Mxi1 | Max interacting protein 1 |
| 910 | TTAAAAACAC | 4 | 0 | 4.0 | 0.02557 |  | NADH dehydrogenase (ubiquinone) Fe-S protein 1 |
| 911 | TTAAACTGAA | 4 | 0 | 4.0 | 0.02557 |  |  |
| 912 | TTAAATAAAT | 4 | 0 | 4.0 | 0.02557 | Plekha5 | Pleckstrin homology domain containing, family A member 5 |
| 913 | TTAAATAATG | 4 | 0 | 4.0 | 0.02557 |  | Hypoxia inducible factor 1, alpha subunit |
| 914 | TTAATGTGAA | 4 | 0 | 4.0 | 0.02557 |  |  |
| 915 | TTACTTGTTT | 4 | 0 | 4.0 | 0.02557 |  |  |
| 916 | TTATCTTTAA | 4 | 0 | 4.0 | 0.02557 | Fer1l3 | Fer-1-like 3, myoferlin (C. elegans) |
| 917 | TTATCTTTAG | 4 | 0 | 4.0 | 0.02557 | Dnm1l | Dynamin 1-like |
| 918 | TTATGTTCAG | 4 | 0 | 4.0 | 0.02557 | Glo1 | Glyoxalase 1 |
| 919 | TTCAAAAGAG | 4 | 0 | 4.0 | 0.02557 | Cant1 | Calcium activated nucleotidase 1 |
| 920 | TTCAAGTCTT | 4 | 0 | 4.0 | 0.02557 | Hist3h2a | Histone cluster 3, H2a |
| 921 | TTCAGAACCT | 4 | 0 | 4.0 | 0.02557 | Arrdc2 | Arrestin domain containing 2 |
| 922 | TTCAGAGAAA | 4 | 0 | 4.0 | 0.02557 | Oasl1 | 2'-5' oligoadenylate synthetase-like 1 |
| 923 | TTCAGCCTTC | 4 | 0 | 4.0 | 0.02557 |  |  |
| 924 | TTCCAGATTG | 4 | 0 | 4.0 | 0.02557 | Reep1 | Receptor accessory protein 1 |
| 925 | TTCTACCGAC | 4 | 0 | 4.0 | 0.02557 | Eepd1 | Endonuclease/exonuclease/phosphatase family domain containing 1 |
| 926 | TTCTAGTCCT | 4 | 0 | 4.0 | 0.02557 | Slc38a6 | Solute carrier family 38, member 6 |
| 927 | TTGATTAGCA | 4 | 0 | 4.0 | 0.02557 | Vamp2 | Vesicle-associated membrane protein 2 |
| 928 | TTGCCACTTT | 4 | 0 | 4.0 | 0.02557 | Saa4 | Serum amyloid A 4 |
| 929 | TTGCCCCGGA | 4 | 0 | 4.0 | 0.02557 |  |  |
| 930 | TTGGCTCAGG | 4 | 0 | 4.0 | 0.02557 | Fstl3 | Follistatin-like 3 |
| 931 | TTGTGCTTCT | 4 | 0 | 4.0 | 0.02557 | Timp3 | Tissue inhibitor of metalloproteinase 3 |
| 932 | TTGTTGTTGA | 4 | 0 | 4.0 | 0.02557 | Calm2 | Calmodulin 2 |
| 933 | TTTCCTTAGA | 4 | 0 | 4.0 | 0.02557 | 4933430I17Rik | RIKEN cDNA 4933430I17 gene |
| 934 | TTTCTTTGTT | 4 | 0 | 4.0 | 0.02557 | Rbm35a | RNA binding motif protein 35A |
| 935 | TTTGAGAAAT | 4 | 0 | 4.0 | 0.02557 | AA408650 | Transcribed locus |
| 936 | TTTGAGTTTT | 4 | 0 | 4.0 | 0.02557 | Tiam2 | T-cell lymphoma invasion and metastasis 2 |
| 937 | TTTGGACCCT | 4 | 0 | 4.0 | 0.02557 |  |  |
| 938 | TTTGTGTTAA | 4 | 0 | 4.0 | 0.02557 | Ctnna1 | Catenin (cadherin associated protein), alpha 1 |
| 939 | TTTGTTTTTC | 4 | 0 | 4.0 | 0.02557 | Wdr48 | WD repeat domain 48 |
| 940 | TTTTAAAAAC | 4 | 0 | 4.0 | 0.02557 | Sp2 | Sp2 transcription factor |
| 941 | TTTTCTGCTT | 4 | 0 | 4.0 | 0.02557 | Hlf | Hepatic leukemia factor |
| 942 | TTTTGAAAGT | 4 | 0 | 4.0 | 0.02557 | Nufip2 | Nuclear fragile X mental retardation protein interacting protein 2 |
| 943 | TTTTTAAGTG | 4 | 0 | 4.0 | 0.02557 | AI597479 | Expressed sequence AI597479 |
| 944 | TTTTTAAGTT | 4 | 0 | 4.0 | 0.02557 | Tbc1d15 | TBC1 domain family, member 15 |
| 945 | TTTTTATACC | 4 | 0 | 4.0 | 0.02557 | Stt3b | STT3, subunit of the oligosaccharyltransferase complex, homolog B (S. cerevisiae) |
| 946 | TTTTTTATGA | 4 | 0 | 4.0 | 0.02557 |  |  |
| 947 | AAGATGGCTC | 23 | 6 | 3.8 | 0.00002 | Sftpc | Surfactant associated protein C |
| 948 | GCGAGCACAC | 15 | 4 | 3.8 | 0.00064 | Lyz2 | Lysozyme 2 |
| 949 | GTCAGAGCTG | 15 | 4 | 3.8 | 0.00064 |  |  |
| 950 | TGTTCAATCA | 15 | 4 | 3.8 | 0.00064 | Sap18 | Sin3-associated polypeptide 18 |
| 951 | AACGCACTGA | 26 | 7 | 3.7 | 0.00001 | Pcolce2 | Procollagen C-endopeptidase enhancer 2 |
| 952 | AAAGTGGGTG | 11 | 3 | 3.7 | 0.00413 | Zbtb22 | Zinc finger and BTB domain containing 22 |
| 953 | AAATAGACGT | 11 | 3 | 3.7 | 0.00413 | Azin1 | Antizyme inhibitor 1 |
| 954 | AACAAGAGTC | 11 | 3 | 3.7 | 0.00413 | Arpc3 | Actin related protein 2/3 complex, subunit 3 |
| 955 | ATACTGGGTT | 11 | 3 | 3.7 | 0.00413 |  |  |
| 956 | CACAGACTGT | 11 | 3 | 3.7 | 0.00413 | Rps27l | Ribosomal protein S27-like |
| 957 | CAGCATAAAT | 11 | 3 | 3.7 | 0.00413 | Appl2 | Adaptor protein, phosphotyrosine interaction, PH domain and leucine zipper containing 2 |
| 958 | CCAGACAGAC | 11 | 3 | 3.7 | 0.00413 |  | Transcribed locus, weakly similar to XP_001479449.1 PREDICTED: similar to pORF1 [Mus musculus] |
| 959 | CCATCTTCTC | 11 | 3 | 3.7 | 0.00413 |  |  |
| 960 | TGATTTTTGT | 11 | 3 | 3.7 | 0.00413 | Dab2 | Disabled homolog 2 (Drosophila) |
| 961 | TGGTCTGGTC | 11 | 3 | 3.7 | 0.00413 | Ifitm2 | Interferon induced transmembrane protein 2 |
| 962 | CTGCTGGTGG | 18 | 5 | 3.6 | 0.00020 | Rabac1 | Rab acceptor 1 (prenylated) |
| 963 | GCCAAGGGTC | 14 | 4 | 3.5 | 0.00130 | Rpl29 | Ribosomal protein L29 |
| 964 | TATTGTTTAC | 14 | 4 | 3.5 | 0.00130 | Psmd7 | Proteasome (prosome, macropain) 26S subunit, non-ATPase, 7 |
| 965 | AAACTTGAGG | 7 | 2 | 3.5 | 0.02529 | 2610209M04Rik | RIKEN cDNA 2610209M04 gene |
| 966 | AATGGCGGCC | 7 | 2 | 3.5 | 0.02529 | Fmod | Fibromodulin |
| 967 | ACCAGAGCAG | 7 | 2 | 3.5 | 0.02529 | Cnih4 | Cornichon homolog 4 (Drosophila) |
| 968 | ACTACCATCA | 7 | 2 | 3.5 | 0.02529 |  | CDNA clone IMAGE:4910858 |
| 969 | AGAGGAAGCT | 7 | 2 | 3.5 | 0.02529 | Nupr1 | Nuclear protein 1 |
| 970 | AGATCCAGCT | 7 | 2 | 3.5 | 0.02529 | OTTMUSG00000016571 | Predicted gene, OTTMUSG00000016571 |
| 971 | AGGCTTTATG | 7 | 2 | 3.5 | 0.02529 | Slc30a1 | Solute carrier family 30 (zinc transporter), member 1 |
| 972 | ATGTCACAAT | 7 | 2 | 3.5 | 0.02529 | Fbn1 | Fibrillin 1 |
| 973 | CAGAACAATG | 7 | 2 | 3.5 | 0.02529 | Adrm1 | Adhesion regulating molecule 1 |
| 974 | CAGGACAAAG | 7 | 2 | 3.5 | 0.02529 | AI450540 | Expressed sequence AI450540 |
| 975 | CAGGCTTGGG | 7 | 2 | 3.5 | 0.02529 | 2210016L21Rik | RIKEN cDNA 2210016L21 gene |
| 976 | CATTATGGGT | 7 | 2 | 3.5 | 0.02529 | Ugt1a1 | UDP glucuronosyltransferase 1 family, polypeptide A6B |
| 977 | CCACACAAGC | 7 | 2 | 3.5 | 0.02529 | Rnf130 | Hypothetical LOC552912 |
| 978 | CCCTGCAGCT | 7 | 2 | 3.5 | 0.02529 | OTTMUSG00000020100 | Hypothetical protein LOC100042904 |
| 979 | CGCCTGCACA | 7 | 2 | 3.5 | 0.02529 | Vps25 | Vacuolar protein sorting 25 (yeast) |
| 980 | CTGGTCTCTG | 7 | 2 | 3.5 | 0.02529 |  | TBC1 domain family, member 9B |
| 981 | CTTAGAAATA | 7 | 2 | 3.5 | 0.02529 | Gm114 | Gene model 114, (NCBI) |
| 982 | CTTATTTCCC | 7 | 2 | 3.5 | 0.02529 | Fgf7 | Fibroblast growth factor 7 |
| 983 | CTTTGTAGTG | 7 | 2 | 3.5 | 0.02529 |  | Protein phosphatase 1, catalytic subunit, beta isoform |
| 984 | GAAAAGCCTC | 7 | 2 | 3.5 | 0.02529 | Lyn | Yamaguchi sarcoma viral (v-yes-1) oncogene homolog |
| 985 | GAAACTCTAC | 7 | 2 | 3.5 | 0.02529 | Cdo1 | Cysteine dioxygenase 1, cytosolic |
| 986 | GAACCCTGAG | 7 | 2 | 3.5 | 0.02529 | Supt5h | Suppressor of Ty 5 homolog (S. cerevisiae) |
| 987 | GAACCCTGGC | 7 | 2 | 3.5 | 0.02529 | Gramd1a | GRAM domain containing 1A |
| 988 | GACCAAAAAT | 7 | 2 | 3.5 | 0.02529 | Tspan15 | Tetraspanin 15 |
| 989 | GACGGAGTGG | 7 | 2 | 3.5 | 0.02529 | Npc2 | Niemann Pick type C2 |
| 990 | GACTGAACAA | 7 | 2 | 3.5 | 0.02529 | Gimap3 | GTPase, IMAP family member 3 |
| 991 | GAGAACTTGC | 7 | 2 | 3.5 | 0.02529 | 6430527G18Rik | RIKEN cDNA 6430527G18 gene |
| 992 | GAGAGGGCCC | 7 | 2 | 3.5 | 0.02529 | 2310007B03Rik | RIKEN cDNA 2310007B03 gene |
| 993 | GAGGTCACTG | 7 | 2 | 3.5 | 0.02529 | Sars | Seryl-aminoacyl-tRNA synthetase |
| 994 | GATCCTTCTA | 7 | 2 | 3.5 | 0.02529 |  |  |
| 995 | GCAATGCAAA | 7 | 2 | 3.5 | 0.02529 |  |  |
| 996 | GCCTTTACGA | 7 | 2 | 3.5 | 0.02529 | Unc119b | Unc-119 homolog B (C. elegans) |
| 997 | GCTATTTGGA | 7 | 2 | 3.5 | 0.02529 | Erbb2ip | Erbb2 interacting protein |
| 998 | GGAGGAAAAA | 7 | 2 | 3.5 | 0.02529 | Ptma | Prothymosin alpha |
| 999 | GGCCTATCTC | 7 | 2 | 3.5 | 0.02529 |  | Transcribed locus |
| 1000 | GGCTGTGAAG | 7 | 2 | 3.5 | 0.02529 | Ctps2 | Cytidine 5'-triphosphate synthase 2 |
| 1001 | GGGGAGAGGG | 7 | 2 | 3.5 | 0.02529 | Man2b2 | Mannosidase 2, alpha B2 |
| 1002 | GTACAAAAAT | 7 | 2 | 3.5 | 0.02529 | Rab1 | RAB1, member RAS oncogene family |
| 1003 | GTCCTGGCCA | 7 | 2 | 3.5 | 0.02529 | Athl1 | ATH1, acid trehalase-like 1 (yeast) |
| 1004 | TAAAATGAGC | 7 | 2 | 3.5 | 0.02529 | AU020094 | Transcribed locus, strongly similar to XP_001061227.1 PREDICTED: similar to interleukin 17D precursor [Rattus norvegicus] |
| 1005 | TAACCGAGAC | 7 | 2 | 3.5 | 0.02529 | Ezr | Ezrin |
| 1006 | TATAGCGGAG | 7 | 2 | 3.5 | 0.02529 | Lyve1 | Lymphatic vessel endothelial hyaluronan receptor 1 |
| 1007 | TCGCGTCGCT | 7 | 2 | 3.5 | 0.02529 | Pan3 | PAN3 polyA specific ribonuclease subunit homolog (S. cerevisiae) |
| 1008 | TGACAATTTT | 7 | 2 | 3.5 | 0.02529 | Tmem131 | Transmembrane protein 131 |
| 1009 | TGACAGAGCC | 7 | 2 | 3.5 | 0.02529 | Dpp3 | Dipeptidylpeptidase 3 |
| 1010 | TGACAGCTGC | 7 | 2 | 3.5 | 0.02529 | S100a9 | S100 calcium binding protein A9 (calgranulin B) |
| 1011 | TGCTGATCGC | 7 | 2 | 3.5 | 0.02529 | Ppp1r13b | Protein phosphatase 1, regulatory (inhibitor) subunit 13B |
| 1012 | TGCTGTTGCT | 7 | 2 | 3.5 | 0.02529 | Tmem14c | Transmembrane protein 14C |
| 1013 | TGGACAAACC | 7 | 2 | 3.5 | 0.02529 | Naaa | N-acylethanolamine acid amidase |
| 1014 | TGTAAATAAA | 7 | 2 | 3.5 | 0.02529 | 1110058L19Rik | RIKEN cDNA 1110058L19 gene |
| 1015 | TTAAATAAAA | 7 | 2 | 3.5 | 0.02529 |  | Transcribed locus |
| 1016 | TTAACGACAA | 7 | 2 | 3.5 | 0.02529 | Foxa1 | Forkhead box A1 |
| 1017 | TTCTTAGTTT | 7 | 2 | 3.5 | 0.02529 | Uso1 | USO1 homolog, vesicle docking protein (yeast) |
| 1018 | TTGGAAATAG | 7 | 2 | 3.5 | 0.02529 |  | Transcribed locus, strongly similar to NP_001119771.1 establishment of cohesion 1 homolog 1 [Rattus norvegicus] |
| 1019 | TTGTTAGAGG | 7 | 2 | 3.5 | 0.02529 | Nap1l1 | Nucleosome assembly protein 1-like 1 |
| 1020 | TTTATTTCTG | 7 | 2 | 3.5 | 0.02529 | Rrs1 | RRS1 ribosome biogenesis regulator homolog (S. cerevisiae) |
| 1021 | TTTGAAACTG | 7 | 2 | 3.5 | 0.02529 | Wdr7 | WD repeat domain 7 |
| 1022 | TTTTAATGTT | 7 | 2 | 3.5 | 0.02529 | Gm1574 | Gene model 1574, (NCBI) |
| 1023 | TTTTTTCCTT | 7 | 2 | 3.5 | 0.02529 | Slc12a7 | Solute carrier family 12, member 7 |
| 1024 | TCAAACTTGG | 17 | 5 | 3.4 | 0.00043 | Fabp5 | Fatty acid binding protein 5, epidermal |
| 1025 | TCTGACAAAC | 17 | 5 | 3.4 | 0.00043 | Tmsb4x | Thymosin, beta 4, X chromosome |
| 1026 | CTCTGTCTGT | 20 | 6 | 3.3 | 0.00014 | Cr1l | Complement component (3b/4b) receptor 1-like |
| 1027 | AAAGCCAAGA | 10 | 3 | 3.3 | 0.00765 | Etfb | Electron transferring flavoprotein, beta polypeptide |
| 1028 | AATGATACTT | 10 | 3 | 3.3 | 0.00765 | Pard6b | Par-6 (partitioning defective 6) homolog beta (C. elegans) |
| 1029 | ACTGCTCCCA | 10 | 3 | 3.3 | 0.00765 |  |  |
| 1030 | ACTGCTTGTC | 10 | 3 | 3.3 | 0.00765 | Aldh1a7 | Aldehyde dehydrogenase family 1, subfamily A7 |
| 1031 | AGACAAGAGA | 10 | 3 | 3.3 | 0.00765 |  | Transcribed locus |
| 1032 | AGTTACTTGA | 10 | 3 | 3.3 | 0.00765 | Srp19 | Signal recognition particle 19 |
| 1033 | ATGCTTCTCA | 10 | 3 | 3.3 | 0.00765 | 545175 | Similar to LOC635138 protein |
| 1034 | ATTAGCTGTC | 10 | 3 | 3.3 | 0.00765 | Plxna1 | Plexin A1 |
| 1035 | CAGTGAAAAA | 10 | 3 | 3.3 | 0.00765 | Dmxl1 | Dmx-like 1 |
| 1036 | CCAAATACAG | 10 | 3 | 3.3 | 0.00765 | Mbc2 | Membrane bound C2 domain containing protein |
| 1037 | CCTGCTTGTC | 10 | 3 | 3.3 | 0.00765 | Aldh1a1 | Aldehyde dehydrogenase family 1, subfamily A1 |
| 1038 | CTTCTCTTGT | 10 | 3 | 3.3 | 0.00765 | BC003266 | CDNA sequence BC003266 |
| 1039 | GCCTAAGGAG | 10 | 3 | 3.3 | 0.00765 |  |  |
| 1040 | GCCTAGAACT | 10 | 3 | 3.3 | 0.00765 | Hbxip | Hepatitis B virus x interacting protein |
| 1041 | GGCTTTGGAG | 10 | 3 | 3.3 | 0.00765 | 1810006K21Rik | RIKEN cDNA 1810006K21 gene |
| 1042 | GGGCAGATTG | 10 | 3 | 3.3 | 0.00765 | Crispld2 | Cysteine-rich secretory protein LCCL domain containing 2 |
| 1043 | GTCAAACGGA | 10 | 3 | 3.3 | 0.00765 | Edf1 | Endothelial differentiation-related factor 1 |
| 1044 | TCCGGCATAG | 10 | 3 | 3.3 | 0.00765 | Ube2z | Ubiquitin-conjugating enzyme E2Z (putative) |
| 1045 | TCCTTGTTCT | 10 | 3 | 3.3 | 0.00765 |  | Transcribed locus |
| 1046 | TGGTGAAGAG | 10 | 3 | 3.3 | 0.00765 | Atp5f1 | ATP synthase, H+ transporting, mitochondrial F0 complex, subunit b, isoform 1 |
| 1047 | TGGTTCTGTT | 10 | 3 | 3.3 | 0.00765 | Dnttip1 | Deoxynucleotidyltransferase, terminal, interacting protein 1 |
| 1048 | TTAAACTGTC | 10 | 3 | 3.3 | 0.00765 | Klhl7 | Kelch-like 7 (Drosophila) |
| 1049 | AAGGAAGAGA | 13 | 4 | 3.3 | 0.00273 | Vim | Vimentin |
| 1050 | ACTAGTGTTG | 13 | 4 | 3.3 | 0.00273 | Pdzd2 | PDZ domain containing 2 |
| 1051 | GCCCAGACCT | 13 | 4 | 3.3 | 0.00273 |  | Sorbitol dehydrogenase |
| 1052 | GGTTGTTTCT | 13 | 4 | 3.3 | 0.00273 | Snx17 | Sorting nexin 17 |
| 1053 | TACCGCGTCA | 13 | 4 | 3.3 | 0.00273 |  |  |
| 1054 | TAGAGACTGC | 13 | 4 | 3.3 | 0.00273 |  |  |
| 1055 | TGTGAAGCCC | 13 | 4 | 3.3 | 0.00273 | Pmp22 | Peripheral myelin protein |
| 1056 | TGTGTGGAAT | 37 | 12 | 3.1 | 0.00000 | Cyp4b1 | Cytochrome P450, family 4, subfamily b, polypeptide 1 |
| 1057 | GTCACTTTCA | 24 | 8 | 3.0 | 0.00003 | Myh11 | Myosin, heavy polypeptide 11, smooth muscle |
| 1058 | AACTTCTGCT | 15 | 5 | 3.0 | 0.00187 | Ifitm3 | Interferon induced transmembrane protein 3 |
| 1059 | GCCACTGCCT | 15 | 5 | 3.0 | 0.00187 | Tmem176a | Transmembrane protein 176A |
| 1060 | CACGGGACCA | 17 | 6 | 2.8 | 0.00099 | Uqcrh | Ubiquinol-cytochrome c reductase hinge protein |
| 1061 | CGGTTGCTGG | 17 | 6 | 2.8 | 0.00099 | LOC768253 | Zinc finger protein 664 |
| 1062 | TATAGTATGT | 17 | 6 | 2.8 | 0.00099 | Glul | Glutamate-ammonia ligase (glutamine synthetase) |
| 1063 | ATTTGTTTTC | 14 | 5 | 2.8 | 0.00273 | Bphl | Biphenyl hydrolase-like (serine hydrolase, breast epithelial mucin-associated antigen) |
| 1064 | CCTGTGTAAC | 14 | 5 | 2.8 | 0.00273 | Slc4a2 | Solute carrier family 4 (anion exchanger), member 2 |
| 1065 | GCTAATGTAC | 14 | 5 | 2.8 | 0.00273 |  |  |
| 1066 | GTTCCAAAGA | 14 | 5 | 2.8 | 0.00273 |  | Transcribed locus, weakly similar to XP_001004338.1 PREDICTED: similar to novel KRAB box and zinc finger, C2H2 type domain containing protein isoform 4 [Mus musculus] |
| 1067 | TGACTCCTTG | 14 | 5 | 2.8 | 0.00273 | Cd164 | CD164 antigen |
| 1068 | AAGCACCCAC | 11 | 4 | 2.8 | 0.00993 | Plvap | Plasmalemma vesicle associated protein |
| 1069 | AATGTGAGTC | 11 | 4 | 2.8 | 0.00993 |  | Tyrosine 3-monooxygenase/tryptophan 5-monooxygenase activation protein, gamma polypeptide |
| 1070 | ACAACAGAGG | 11 | 4 | 2.8 | 0.00993 | Ptgfrn | Prostaglandin F2 receptor negative regulator |
| 1071 | AGCCCAGATC | 11 | 4 | 2.8 | 0.00993 | Ext2 | Exostoses (multiple) 2 |
| 1072 | AGCCTATGAT | 11 | 4 | 2.8 | 0.00993 | Agps | Alkylglycerone phosphate synthase |
| 1073 | CAAAACTGTA | 11 | 4 | 2.8 | 0.00993 | Notch1 | Notch gene homolog 1 (Drosophila) |
| 1074 | CCTCGGGGGC | 11 | 4 | 2.8 | 0.00993 |  | Transcribed locus |
| 1075 | CCTCTCTGGA | 11 | 4 | 2.8 | 0.00993 | Hba-a1 | Hemoglobin alpha, adult chain 1 |
| 1076 | CTATGGGCTG | 11 | 4 | 2.8 | 0.00993 | Mobkl1b | MOB1, Mps One Binder kinase activator-like 1B (yeast) |
| 1077 | TAACAAGCCT | 11 | 4 | 2.8 | 0.00993 | Nfib | Nuclear factor I/B |
| 1078 | TGGCTCACAA | 11 | 4 | 2.8 | 0.00993 | 5430410E06Rik | Histocompatibility 2, D region locus 1 |
| 1079 | AGGCATTCAG | 43 | 16 | 2.7 | 0.00000 | Lyz1 | Lysozyme 1 |
| 1080 | AAAACCTGTA | 8 | 3 | 2.7 | 0.02909 |  | Transcribed locus |
| 1081 | AATAAACTTA | 8 | 3 | 2.7 | 0.02909 |  | Ras-related GTP binding A |
| 1082 | ACCTGACCAG | 8 | 3 | 2.7 | 0.02909 | Arih2 | Ariadne homolog 2 (Drosophila) |
| 1083 | AGAGAGACAA | 8 | 3 | 2.7 | 0.02909 | Wisp2 | WNT1 inducible signaling pathway protein 2 |
| 1084 | AGCCTCGCGT | 8 | 3 | 2.7 | 0.02909 | Cd93 | CD93 antigen |
| 1085 | AGGGCAGAAA | 8 | 3 | 2.7 | 0.02909 | EG626571 | Predicted gene, EG626571 |
| 1086 | AGTTCAAACC | 8 | 3 | 2.7 | 0.02909 |  | SEC11 homolog A (S. cerevisiae) |
| 1087 | ATCTCCTTGG | 8 | 3 | 2.7 | 0.02909 | Rab28 | RAB28, member RAS oncogene family |
| 1088 | ATGCGGAAAA | 8 | 3 | 2.7 | 0.02909 |  |  |
| 1089 | CAAAATACAT | 8 | 3 | 2.7 | 0.02909 | Maged1 | Melanoma antigen, family D, 1 |
| 1090 | CACCTGCTTT | 8 | 3 | 2.7 | 0.02909 |  | Phosphoribosylaminoimidazole carboxylase, phosphoribosylaminoribosylaminoimidazole, succinocarboxamide synthetase |
| 1091 | CATCTTTATG | 8 | 3 | 2.7 | 0.02909 | Isca1 | Iron-sulfur cluster assembly 1 homolog (S. cerevisiae) |
| 1092 | CGTCGAAAAA | 8 | 3 | 2.7 | 0.02909 | Specc1l | SPECC1-like |
| 1093 | CTACAGAGAT | 8 | 3 | 2.7 | 0.02909 |  | Transcribed locus, strongly similar to NP_036788.1 secreted acidic cysteine rich glycoprotein [Rattus norvegicus] |
| 1094 | CTTCCCGGCT | 8 | 3 | 2.7 | 0.02909 | BC017612 | CDNA sequence BC017612 |
| 1095 | GACTTCTACC | 8 | 3 | 2.7 | 0.02909 |  | RIKEN cDNA 1110014K08 gene |
| 1096 | GCATTTTTAT | 8 | 3 | 2.7 | 0.02909 | Fgfr4 | Fibroblast growth factor receptor 4 |
| 1097 | GCCTGTGTAT | 8 | 3 | 2.7 | 0.02909 | Tead1 | TEA domain family member 1 |
| 1098 | GGTGTTTGCA | 8 | 3 | 2.7 | 0.02909 | Mdm4 | Transformed mouse 3T3 cell double minute 4 |
| 1099 | GTTAAGTTAA | 8 | 3 | 2.7 | 0.02909 | Ndufc1 | NADH dehydrogenase (ubiquinone) 1, subcomplex unknown, 1 |
| 1100 | GTTACAGGTA | 8 | 3 | 2.7 | 0.02909 | Ogt | O-linked N-acetylglucosamine (GlcNAc) transferase (UDP-N-acetylglucosamine:polypeptide-N-acetylglucosaminyl transferase) |
| 1101 | GTTCCTGCTT | 8 | 3 | 2.7 | 0.02909 | Ric8 | Resistance to inhibitors of cholinesterase 8 homolog (C. elegans) |
| 1102 | GTTTTCAATA | 8 | 3 | 2.7 | 0.02909 | Rabl4 | RAB, member of RAS oncogene family-like 4 |
| 1103 | TATCTTCCGC | 8 | 3 | 2.7 | 0.02909 |  | Transmembrane protein 98 |
| 1104 | TGGAACAATG | 8 | 3 | 2.7 | 0.02909 | Srgn | Serglycin |
| 1105 | TGGCCAAGTG | 8 | 3 | 2.7 | 0.02909 | Sox18 | SRY-box containing gene 18 |
| 1106 | TGGGCAGAAG | 8 | 3 | 2.7 | 0.02909 |  |  |
| 1107 | TTACTTACCA | 8 | 3 | 2.7 | 0.02909 |  | High mobility group box transcription factor 1 |
| 1108 | TTTCTTTCGG | 8 | 3 | 2.7 | 0.02909 |  | Eukaryotic translation initiation factor 5 |
| 1109 | ATACTGGTCC | 26 | 10 | 2.6 | 0.00009 |  |  |
| 1110 | ATCGTTGTAA | 13 | 5 | 2.6 | 0.00568 | Dhx15 | DEAH (Asp-Glu-Ala-His) box polypeptide 15 |
| 1111 | ATGTACTAAA | 13 | 5 | 2.6 | 0.00568 | Tfg | Trk-fused gene |
| 1112 | CAAACTGTGC | 13 | 5 | 2.6 | 0.00568 | Krt18 | Keratin 18 |
| 1113 | CAAGCACTTT | 13 | 5 | 2.6 | 0.00568 |  | MAP kinase-interacting serine/threonine kinase 2 |
| 1114 | CCCTGCCCTG | 13 | 5 | 2.6 | 0.00568 | Aip | Aryl-hydrocarbon receptor-interacting protein |
| 1115 | TCAAACTGTG | 13 | 5 | 2.6 | 0.00568 |  | Transcribed locus |
| 1116 | TCTTCTAATC | 13 | 5 | 2.6 | 0.00568 | Hnrnpa3 | Heterogeneous nuclear ribonucleoprotein A3 |
| 1117 | TGAGCCACTG | 13 | 5 | 2.6 | 0.00568 |  | Transposon-related mRNA, partial sequence |
| 1118 | GCTTTGAATG | 31 | 12 | 2.6 | 0.00003 | Atpif1 | ATPase inhibitory factor 1 |
| 1119 | CCTTTGGCAA | 18 | 7 | 2.6 | 0.00117 | Psmb7 | Proteasome (prosome, macropain) subunit, beta type 7 |
| 1120 | GCAACAACAC | 18 | 7 | 2.6 | 0.00117 |  |  |
| 1121 | GCCCTGGTGC | 18 | 7 | 2.6 | 0.00117 | Ypel3 | Yippee-like 3 (Drosophila) |
| 1122 | GGGGAATAAA | 18 | 7 | 2.6 | 0.00117 | Ltbp2 | Latent transforming growth factor beta binding protein 2 |
| 1123 | TTGGGCCAGA | 20 | 8 | 2.5 | 0.00079 | D13Ertd332e | Cytochrome c oxidase, subunit VIIc |
| 1124 | ATCCAGCACA | 15 | 6 | 2.5 | 0.00340 |  | FERM, RhoGEF (Arhgef) and pleckstrin domain protein 1 (chondrocyte-derived) |
| 1125 | CCCTCTTCTG | 15 | 6 | 2.5 | 0.00340 | BC028528 | CDNA sequence BC028528 |
| 1126 | CCTGAGTCCA | 15 | 6 | 2.5 | 0.00340 |  |  |
| 1127 | CTTATTCCAG | 15 | 6 | 2.5 | 0.00340 | Phlda3 | Pleckstrin homology-like domain, family A, member 3 |
| 1128 | GGAAGATGCT | 15 | 6 | 2.5 | 0.00340 | Bex2 | Brain expressed X-linked 2 |
| 1129 | TCACTTGTCA | 15 | 6 | 2.5 | 0.00340 | Tm2d1 | TM2 domain containing 1 |
| 1130 | TCCTCGTGAA | 15 | 6 | 2.5 | 0.00340 | Ear2 | Eosinophil-associated, ribonuclease A family, member 2 |
| 1131 | AACTACAGCT | 10 | 4 | 2.5 | 0.01904 | 0610039K10Rik | Serine incorporator 3 |
| 1132 | AACTGTCCCT | 10 | 4 | 2.5 | 0.01904 | Hras1 | Harvey rat sarcoma virus oncogene 1 |
| 1133 | ATACACGAAA | 10 | 4 | 2.5 | 0.01904 | Ing1 | Inhibitor of growth family, member 1 |
| 1134 | ATGATGAGAG | 10 | 4 | 2.5 | 0.01904 |  | Transcribed locus |
| 1135 | ATTTTTGAGG | 10 | 4 | 2.5 | 0.01904 | 4931406C07Rik | RIKEN cDNA 4931406C07 gene |
| 1136 | CACACTTGTA | 10 | 4 | 2.5 | 0.01904 | Orai1 | ORAI calcium release-activated calcium modulator 1 |
| 1137 | CAGACGGAAG | 10 | 4 | 2.5 | 0.01904 |  |  |
| 1138 | CAGATAATGT | 10 | 4 | 2.5 | 0.01904 | Cept1 | Choline/ethanolaminephosphotransferase 1 |
| 1139 | CCGACGGCGC | 10 | 4 | 2.5 | 0.01904 |  |  |
| 1140 | CCTGTCTACT | 10 | 4 | 2.5 | 0.01904 | Psma6 | Proteasome (prosome, macropain) subunit, alpha type 6 |
| 1141 | CTGCCTTGTT | 10 | 4 | 2.5 | 0.01904 | BC063749 | CDNA sequence BC063749 |
| 1142 | CTGTCCATCT | 10 | 4 | 2.5 | 0.01904 | Scgb1a1 | Secretoglobin, family 1A, member 1 (uteroglobin) |
| 1143 | CTTAATTGAC | 10 | 4 | 2.5 | 0.01904 | 4933426M11Rik | RIKEN cDNA 4933426M11 gene |
| 1144 | GGTTTCTTTT | 10 | 4 | 2.5 | 0.01904 | Slc4a8 | Solute carrier family 4 (anion exchanger), member 8 |
| 1145 | GTGGTCCATA | 10 | 4 | 2.5 | 0.01904 |  | Transcribed locus, strongly similar to NP_036788.1 secreted acidic cysteine rich glycoprotein [Rattus norvegicus] |
| 1146 | TAGACCAGAC | 10 | 4 | 2.5 | 0.01904 | BC004004 | CDNA sequence BC004004 |
| 1147 | TCTGTTTCTG | 10 | 4 | 2.5 | 0.01904 | Tcfe2a | Transcription factor E2a |
| 1148 | TGAAATGGTT | 10 | 4 | 2.5 | 0.01904 | Commd3 | COMM domain containing 3 |
| 1149 | TGCAATATGG | 10 | 4 | 2.5 | 0.01904 |  |  |
| 1150 | TGGGTGTCTT | 10 | 4 | 2.5 | 0.01904 | Skp1a | S-phase kinase-associated protein 1A |
| 1151 | TGTGTTCCCA | 10 | 4 | 2.5 | 0.01904 |  | Ubiquitin protein ligase E3B |
| 1152 | TTCCATTTAA | 10 | 4 | 2.5 | 0.01904 | C1d | Nuclear DNA binding protein |
| 1153 | TTCTTGGGGA | 10 | 4 | 2.5 | 0.01904 | Trim41 | Tripartite motif-containing 41 |
| 1154 | TTGTACAACA | 10 | 4 | 2.5 | 0.01904 | Memo1 | Mediator of cell motility 1 |
| 1155 | TTTTGTACTT | 10 | 4 | 2.5 | 0.01904 | 2900064A13Rik | RIKEN cDNA 2900064A13 gene |
| 1156 | TTTTGTTTAT | 10 | 4 | 2.5 | 0.01904 |  | Guanosine diphosphate (GDP) dissociation inhibitor 2 |
| 1157 | GCATACGGCG | 17 | 7 | 2.4 | 0.00248 | D830035I06 | ATP synthase, H+ transporting, mitochondrial F1F0 complex, subunit e |
| 1158 | GTAATCTGCT | 17 | 7 | 2.4 | 0.00248 |  |  |
| 1159 | GTTCAGCAAA | 17 | 7 | 2.4 | 0.00248 |  | RNA binding motif protein 39 |
| 1160 | AGGAGGGCCT | 29 | 12 | 2.4 | 0.00005 | Bcl3 | B-cell leukemia/lymphoma 3 |
| 1161 | CCCCTTTTAC | 31 | 13 | 2.4 | 0.00003 | Msn | Moesin |
| 1162 | CAGGGATGTT | 21 | 9 | 2.3 | 0.00087 |  | Transcribed locus |
| 1163 | GAATCCAACT | 21 | 9 | 2.3 | 0.00087 | Ndufb11 | NADH dehydrogenase (ubiquinone) 1 beta subcomplex, 11 |
| 1164 | AAAGATTAAT | 14 | 6 | 2.3 | 0.00662 | Snx3 | Sorting nexin 3 |
| 1165 | ATCTGGGATC | 14 | 6 | 2.3 | 0.00662 | Cyp2b10 | Cytochrome P450, family 2, subfamily b, polypeptide 10 |
| 1166 | CAAGTGCACT | 14 | 6 | 2.3 | 0.00662 | Map1lc3b | Microtubule-associated protein 1 light chain 3 beta |
| 1167 | CTCAGTATCC | 14 | 6 | 2.3 | 0.00662 | Lrp1 | Low density lipoprotein receptor-related protein 1 |
| 1168 | GAAGTGCAGA | 14 | 6 | 2.3 | 0.00662 |  |  |
| 1169 | GATACCATTA | 14 | 6 | 2.3 | 0.00662 | Scp2 | Sterol carrier protein 2, liver |
| 1170 | GCGTCGAGCC | 14 | 6 | 2.3 | 0.00662 | 2810428I15Rik | RIKEN cDNA 2810428I15 gene |
| 1171 | GTGGACTCAA | 14 | 6 | 2.3 | 0.00662 | Ifitm1 | Interferon induced transmembrane protein 1 |
| 1172 | CGGGAGATGC | 23 | 10 | 2.3 | 0.00053 | Atp5o | ATP synthase, H+ transporting, mitochondrial F1 complex, O subunit |
| 1173 | ATCAACACCG | 39 | 17 | 2.3 | 0.00001 | AF085738 | GNAS (guanine nucleotide binding protein, alpha stimulating) complex locus |
| 1174 | TCTAAGTACG | 39 | 17 | 2.3 | 0.00001 |  |  |
| 1175 | TCAATAAAGC | 27 | 12 | 2.3 | 0.00015 | Scn3b | Sodium channel, voltage-gated, type III, beta |
| 1176 | GTAGAGATGG | 18 | 8 | 2.3 | 0.00283 | Cd36 | CD36 antigen |
| 1177 | CCTTTGAGAT | 29 | 13 | 2.2 | 0.00015 | Rps5 | Ribosomal protein S5 |
| 1178 | ATTTCCCGAG | 20 | 9 | 2.2 | 0.00159 | Arpc5 | Actin related protein 2/3 complex, subunit 5 |
| 1179 | CAAGGCAATG | 20 | 9 | 2.2 | 0.00159 | Serpinb6b | Serine (or cysteine) peptidase inhibitor, clade B, member 6b |
| 1180 | GGATTGTCAA | 20 | 9 | 2.2 | 0.00159 | Slk | STE20-like kinase (yeast) |
| 1181 | GGGATGGACG | 20 | 9 | 2.2 | 0.00159 | C4a | Complement component 4B (Childo blood group) |
| 1182 | TTGATGTACA | 20 | 9 | 2.2 | 0.00159 | Sfrs11 | Splicing factor, arginine/serine-rich 11 |
| 1183 | GTTTGTCAGC | 31 | 14 | 2.2 | 0.00009 | Fgfr3 | Fibroblast growth factor receptor 3 |
| 1184 | AGGCAAAGAG | 42 | 19 | 2.2 | 0.00001 | Cxcl15 | Chemokine (C-X-C motif) ligand 15 |
| 1185 | AAATGCAATA | 11 | 5 | 2.2 | 0.01948 | Nfyc | Nuclear transcription factor-Y gamma |
| 1186 | AATCACTGGC | 11 | 5 | 2.2 | 0.01948 | Ict1 | Immature colon carcinoma transcript 1 |
| 1187 | CTACTAGTTT | 11 | 5 | 2.2 | 0.01948 | D5Ertd77e | RNA binding motif protein 47 |
| 1188 | CTTGGCTACC | 11 | 5 | 2.2 | 0.01948 | Igk-V4 | RIKEN cDNA 9530068E07 gene |
| 1189 | GAGCCTCACA | 11 | 5 | 2.2 | 0.01948 |  | Protein kinase inhibitor, gamma |
| 1190 | GCTCCCTCTG | 11 | 5 | 2.2 | 0.01948 | LOC544737 | RIKEN cDNA 4921506J03 gene |
| 1191 | GTGGCGCACG | 11 | 5 | 2.2 | 0.01948 | Hyal3 | Hyaluronoglucosaminidase 3 |
| 1192 | TATAGCCCTG | 11 | 5 | 2.2 | 0.01948 | Ccl6 | Chemokine (C-C motif) ligand 6 |
| 1193 | TGGGTTAGAC | 11 | 5 | 2.2 | 0.01948 | Pfdn1 | Prefoldin 1 |
| 1194 | TGTTCACACT | 11 | 5 | 2.2 | 0.01948 |  | Transcribed locus |
| 1195 | TTGTATAATA | 11 | 5 | 2.2 | 0.01948 | Slc25a5 | Solute carrier family 25 (mitochondrial carrier, adenine nucleotide translocator), member 5 |
| 1196 | TTTTAAACTT | 11 | 5 | 2.2 | 0.01948 | D4Ertd22e | DNA segment, Chr 4, ERATO Doi 22, expressed |
| 1197 | CACCACCACA | 39 | 18 | 2.2 | 0.00001 | Rpl27a | Ribosomal protein L27a |
| 1198 | AGCTCACCAA | 13 | 6 | 2.2 | 0.01148 | Efha1 | EF hand domain family A1 |
| 1199 | CCACACTGTC | 13 | 6 | 2.2 | 0.01148 | LOC100041985 | Capping protein (actin filament) muscle Z-line, alpha 1 |
| 1200 | CTTTTTCTGA | 13 | 6 | 2.2 | 0.01148 |  |  |
| 1201 | GCACCTCTTA | 13 | 6 | 2.2 | 0.01148 | Hnrnpul1 | Heterogeneous nuclear ribonucleoprotein U-like 1 |
| 1202 | GTCACACCAC | 13 | 6 | 2.2 | 0.01148 |  | Transcribed locus |
| 1203 | TAGAAACCAA | 13 | 6 | 2.2 | 0.01148 |  |  |
| 1204 | TAGAGTGTGC | 13 | 6 | 2.2 | 0.01148 |  | Transcribed locus |
| 1205 | TATTTTACCG | 13 | 6 | 2.2 | 0.01148 | Arhgap1 | Rho GTPase activating protein 1 |
| 1206 | TGAACCCACT | 13 | 6 | 2.2 | 0.01148 | Cox6a1 | Cytochrome c oxidase, subunit VI a, polypeptide 1 |
| 1207 | TGCCTTACTT | 13 | 6 | 2.2 | 0.01148 | Pdcd6 | Programmed cell death 6 |
| 1208 | TGTACCCAGG | 13 | 6 | 2.2 | 0.01148 | Ganab | Alpha glucosidase 2 alpha neutral subunit |
| 1209 | TTCTAGTTGC | 13 | 6 | 2.2 | 0.01148 | Ptplad1 | Protein tyrosine phosphatase-like A domain containing 1 |
| 1210 | TGTTCATCTT | 43 | 20 | 2.2 | 0.00000 | Col3a1 | Collagen, type III, alpha 1 |
| 1211 | CTGCTATCCG | 58 | 27 | 2.1 | 0.00000 |  | Ribosomal protein L5 |
| 1212 | ACACTTTTGA | 15 | 7 | 2.1 | 0.00711 | Pls3 | Plastin 3 (T-isoform) |
| 1213 | AGCTCTAAAA | 15 | 7 | 2.1 | 0.00711 |  |  |
| 1214 | TCTTTTTAAA | 15 | 7 | 2.1 | 0.00711 | Ifi205 | Interferon activated gene 205 |
| 1215 | CAAACTCTCA | 260 | 122 | 2.1 | 0.00000 | Sparc | Secreted acidic cysteine rich glycoprotein |
| 1216 | GCCACCGTCC | 34 | 16 | 2.1 | 0.00006 | Nedd4 | Neural precursor cell expressed, developmentally down-regulated gene 4 |
| 1217 | TGGCTTTTCT | 34 | 16 | 2.1 | 0.00006 |  | Spectrin beta 2 |
| 1218 | AAGCTCCACG | 17 | 8 | 2.1 | 0.00471 |  |  |
| 1219 | GCTTCTTCAG | 17 | 8 | 2.1 | 0.00471 | Dad1 | Defender against cell death 1 |
| 1220 | TACATTCTAT | 17 | 8 | 2.1 | 0.00471 | Mcl1 | Myeloid cell leukemia sequence 1 |
| 1221 | TTGTTCTGAC | 17 | 8 | 2.1 | 0.00471 | Gpi1 | Glucose phosphate isomerase 1 |
| 1222 | TGGCTCGGTC | 87 | 41 | 2.1 | 0.00000 | Actg1 | Actin, gamma, cytoplasmic 1 |
| 1223 | CCAGAGGCTG | 63 | 30 | 2.1 | 0.00000 | AI467606 | Expressed sequence AI467606 |
| 1224 | CTTCCCTGTT | 21 | 10 | 2.1 | 0.00173 | Ctnna1 | Catenin (cadherin associated protein), alpha 1 |
| 1225 | GGTCAGTCGG | 197 | 94 | 2.1 | 0.00000 |  |  |
| 1226 | TCTCACCACC | 74 | 36 | 2.1 | 0.00000 |  | Elastin |
| 1227 | TGGTTGCTGG | 69 | 34 | 2.0 | 0.00000 | Padi2 | Peptidyl arginine deiminase, type II |
| 1228 | AGATCTATAC | 34 | 17 | 2.0 | 0.00009 | Rpl7 | Ribosomal protein L7 |
| 1229 | GCTCTAGCCA | 26 | 13 | 2.0 | 0.00071 | Col4a2 | Collagen, type IV, alpha 2 |
| 1230 | AATAAAAACT | 20 | 10 | 2.0 | 0.00292 | Ubl5 | Ubiquitin-like 5 |
| 1231 | ATCCTGTGCT | 20 | 10 | 2.0 | 0.00292 |  | CD9 antigen |
| 1232 | GCCAGAAGGC | 20 | 10 | 2.0 | 0.00292 | Mastl | Microtubule associated serine/threonine kinase-like |
| 1233 | CTATTTCAAA | 18 | 9 | 2.0 | 0.00466 |  |  |
| 1234 | GCACCTTCAA | 18 | 9 | 2.0 | 0.00466 | Icam2 | Intercellular adhesion molecule 2 |
| 1235 | AAGACAGTGT | 14 | 7 | 2.0 | 0.01380 |  | Transcribed locus |
| 1236 | ACTGAAGCAA | 14 | 7 | 2.0 | 0.01380 |  | Scavenger receptor class B, member 1 |
| 1237 | ATGTCAGTGT | 14 | 7 | 2.0 | 0.01380 | Tm4sf1 | Transmembrane 4 superfamily member 1 |
| 1238 | CACTTGCAGT | 14 | 7 | 2.0 | 0.01380 | Suv420h1 | Transcribed locus |
| 1239 | CTTAAATCTG | 14 | 7 | 2.0 | 0.01380 | Dnaja1 | DnaJ (Hsp40) homolog, subfamily A, member 1 |
| 1240 | GGAGGTGGAG | 14 | 7 | 2.0 | 0.01380 | Grn | Granulin |
| 1241 | GGGTGCGTCT | 14 | 7 | 2.0 | 0.01380 | Aamp | Angio-associated migratory protein |
| 1242 | GTAGTCAACA | 14 | 7 | 2.0 | 0.01380 |  |  |
| 1243 | GTCCTATAAG | 14 | 7 | 2.0 | 0.01380 | Mlf2 | Myeloid leukemia factor 2 |
| 1244 | TCATTTGGTG | 14 | 7 | 2.0 | 0.01380 | Lox | Lysyl oxidase |
| 1245 | AAATATGTCG | 10 | 5 | 2.0 | 0.03832 |  |  |
| 1246 | AGGACGGAGG | 10 | 5 | 2.0 | 0.03832 | Trafd1 | TRAF type zinc finger domain containing 1 |
| 1247 | ATACTGGTTT | 10 | 5 | 2.0 | 0.03832 |  |  |
| 1248 | CTGTACTGTG | 10 | 5 | 2.0 | 0.03832 | Slmo2 | Slowmo homolog 2 (Drosophila) |
| 1249 | CTTACACGGA | 10 | 5 | 2.0 | 0.03832 |  |  |
| 1250 | CTTGCAATCT | 10 | 5 | 2.0 | 0.03832 | Aadacl1 | Arylacetamide deacetylase-like 1 |
| 1251 | TAATTTTTTT | 10 | 5 | 2.0 | 0.03832 | Pex2 | Peroxisome biogenesis factor 2 |
| 1252 | TACAATTGAT | 10 | 5 | 2.0 | 0.03832 |  | Transcribed locus |
| 1253 | TCAGCCCAGG | 10 | 5 | 2.0 | 0.03832 |  | Neuroepithelial cell transforming gene 1 |
| 1254 | TGGCCCAAAA | 10 | 5 | 2.0 | 0.03832 | Atp6v0e2 | ATPase, H+ transporting, lysosomal V0 subunit E2 |
